# Protein kinases in neurodegenerative diseases: current understandings and implications for drug discovery

**DOI:** 10.1038/s41392-025-02179-x

**Published:** 2025-05-07

**Authors:** Xiaolei Wu, Zhangzhong Yang, Jinjun Zou, Huile Gao, Zhenhua Shao, Chuanzhou Li, Peng Lei

**Affiliations:** 1 Department of Neurology and State Key Laboratory of Biotherapy, National Clinical Research Center for Geriatrics, West China Hospital, Sichuan University, Chengdu, Sichuan China; 2 Key Laboratory of Drug Targeting and Drug Delivery Systems, West China School of Pharmacy, Sichuan University, Chengdu, China; 3 Division of Nephrology and Kidney Research Institute, State Key Laboratory of Biotherapy and Cancer Center, West China Hospital, Sichuan University, Chengdu, Sichuan China; 4 Department of Medical Genetics, School of Basic Medicine, Tongji Medical College, Huazhong University of Science and Technology, Wuhan, China

**Keywords:** Neuroscience, Diseases of the nervous system, Neurodevelopmental disorders

## Abstract

Neurodegenerative diseases (e.g., Alzheimer’s, Parkinson’s, Huntington’s disease, and Amyotrophic Lateral Sclerosis) are major health threats for the aging population and their prevalences continue to rise with the increasing of life expectancy. Although progress has been made, there is still a lack of effective cures to date, and an in-depth understanding of the molecular and cellular mechanisms of these neurodegenerative diseases is imperative for drug development. Protein phosphorylation, regulated by protein kinases and protein phosphatases, participates in most cellular events, whereas aberrant phosphorylation manifests as a main cause of diseases. As evidenced by pharmacological and pathological studies, protein kinases are proven to be promising therapeutic targets for various diseases, such as cancers, central nervous system disorders, and cardiovascular diseases. The mechanisms of protein phosphatases in pathophysiology have been extensively reviewed, but a systematic summary of the role of protein kinases in the nervous system is lacking. Here, we focus on the involvement of protein kinases in neurodegenerative diseases, by summarizing the current knowledge on the major kinases and related regulatory signal transduction pathways implicated in diseases. We further discuss the role and complexity of kinase–kinase networks in the pathogenesis of neurodegenerative diseases, illustrate the advances of clinical applications of protein kinase inhibitors or novel kinase-targeted therapeutic strategies (such as antisense oligonucleotides and gene therapy) for effective prevention and early intervention.

## Introduction

**Fig. 1:**
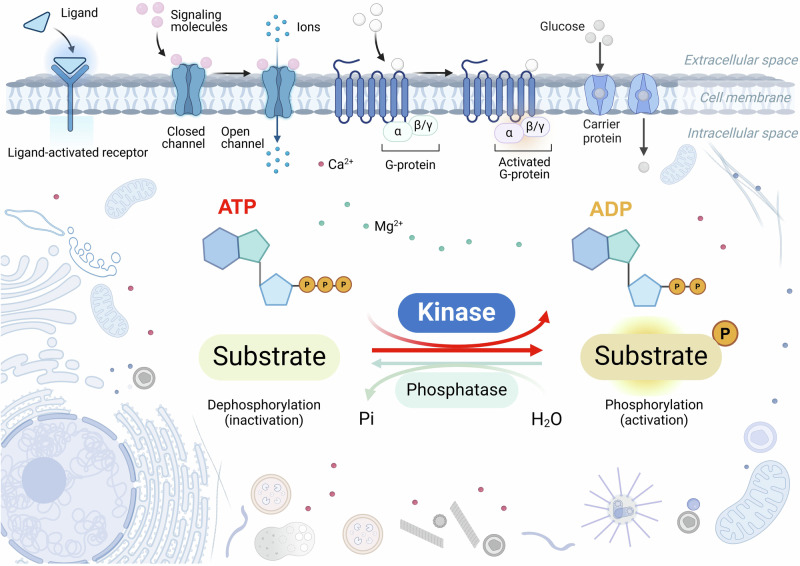
Schematic diagram of the general working mechanism of kinases. Kinases catalyze the transfer of the γ-phosphate group of ATP to the specific substrate, making the specific amino acid of the substrate phosphorylated. This figure was created with BioRender.com

Kinases catalyze the transfer of the γ-phosphate group of ATP to the specific substrate,^1^ making the specific amino acid of the substrate phosphorylated (Fig. 1). Considering the type of substrates, kinases can be divided into protein kinases, lipid kinases, carbohydrate kinases, and other kinases (including Riboflavin kinase and Thymidine kinase, etc.), the most important group of which is protein kinase.^2^ The first protein kinase that was biochemically characterized in 1955 is phosphorylase kinase, which catalyzes the ATP-dependent phosphorylation of the specific phosphorylase.^1^ Since then, 518 human protein kinases to date (including 478 human eukaryotic protein kinases and 40 atypical protein kinases) and more than 900 genes encoding proteins with kinase activity have been identified.^3,4^ These protein kinases are classified into Ser/Thr kinases (385 members), tyrosine kinases (TK, 90 members), and tyrosine kinase-like kinases (TKL, 43 members) based on the targeted phosphate group of substrate residue.^3^ Protein kinases play indispensable roles in human diseases, especially in cancer and neurodegenerative diseases, and have been widely considered as drug targets for precision therapy.^5^ Small-molecule kinase inhibitors such as imatinib (inhibitor for TK, including BCR-Abl) and kinase-directed biological molecules such as margetuximab (a monoclonal antibody drug targeting human epidermal growth factor receptor 2 (HER2)-TK) have already been approved clinically for cancer therapy worldwide.^1^ However, despite these advancements, no kinase-related therapies targeting neurodegeneration have yet been approved.

Neurodegenerative diseases, mainly affecting the brain, are a general term for a series of diseases caused by the progressive loss of structure or function of neurons, mainly including Alzheimer’s disease (AD), Parkinson’s disease (PD), Huntington’s disease (HD), and Amyotrophic Lateral Sclerosis (ALS). A shared characteristic of these diseases is the deposition of misfolded protein aggregates owing to the increased resistance of degradation of specific mutant proteins or the excessive accumulation of wild-type proteins. Aging is a major risk factor shared in neurodegenerative diseases. During brain aging, the metabolic regulation of neurons, neuronal development, and the immune microenvironment change, leading to a dysregulated molecular network around neurons, thereby promoting cognitive dysfunction.^6^ As the population ages, the rate of neurodegenerative diseases is increasing. For example, more than 55 million people currently live with dementia, and it is estimated that by 2050, ~150 million people globally will be affected by dementia, which will bring an economic burden of nearly $10 trillion^7^ and immeasurable losses to patients and their families.

However, these neurodegenerative diseases are currently incurable, and the treatments can only relieve symptoms and delay the progress of the diseases. Strategies under development for neurodegenerative diseases mainly include small molecules, antisense oligonucleotides (ASOs), gene therapy, and cell therapy. Immunotherapies targeting β-amyloid (Aβ) (Aducanumab,^8^ Lecanemab,^9^ Donanemab,^10^ all of which have been approved), tau (BIIB080,^11^ NI0752,^12^ JNJ-63733657,^12,13^ ACI-35^13^), and α-synuclein (α-syn) (Cinpanemab,^14^ Prasinezumab,^15^ Lu AF82422^16^) reduce internalization and diffusion of extracellular protein aggregates into neighboring cells in AD and PD.^17^ Drugs that maximize the quality of life for ALS patients have been approved, including, Relyvrio,^18^ Riluzole,^19^ and Eladarone.^19^ In addition to drug treatment, ASO therapies targeting several ALS causative genes (*SOD1*, *C9orf72*, *ATXN-2*, *FUS*), including ISIS333611,^20^ Tofersen (approved),^21^ BIIB078,^22^ and BIIB105^23^ are entered in clinical studies, and gene therapy drugs using adeno-associated virus as a vector are also under development. Nevertheless, there is still an unmet need for drugs or new potential strategies that can reverse neurodegeneration.

Protein phosphorylation is a crucial type of post-translational modification in neurodegeneration that has been most extensively investigated. The process of adding phosphate groups to the substrate, is catalyzed by kinases, while phosphatases are enzymes that remove phosphate groups. The addition or removal of phosphate groups results in the gaining or losing function of the substrate, thereby positively or negatively regulating the subsequent pathophysiological processes. In pathological processes, such as tangle formation in AD, residues in the proline-rich and microtubule-binding regions of tau protein are highly susceptible to phosphorylation modification. Phosphorylation of tau at Thr231 and Ser262 results in decreased affinity for microtubules.^24^ Abnormal hyperphosphorylation of tau increases its self-aggregation, leading to its mislocalization in neurons and impairment of synaptic functions.^25,26^ However, during physiological processes, such as axon formation, phosphorylations of tau at Ser199/202 and Thr205 sites are essential for axon formation, as evidenced by that about 80% of tau is phosphorylated at these sites in the cell body and proximal axons, while about 20% of tau is phosphorylated in the growth cones to regulate axonogenesis.^27^ In PD, hyperphosphorylation of α-syn at Ser129 leads to its misfolding and aggregation, forming pathological Lewy-bodies and Lewy-neurites.^28^ On the other hand, the dynamic changes of phosphorylation and dephosphorylation at Ser129 of α-syn at physiological levels are essential for fine-tuning of neuronal synaptic transmission.^28^ Furthermore, hyperphosphorylation of 43 kDa transactive response DNA-binding protein (TDP-43) protein at Ser409/410 leads to its mislocalization and aggregation in the neuronal cytoplasm in ALS.^29^ These observations together have highlighted the great potential to target abnormal protein phosphorylation to treat neurodegenerative diseases.

Protein kinases, as protein phosphorylation writer, are thereby crucial for neuronal homeostasis in neurodegenerative diseases as supported by numerous evidence. For example, the phosphatidylinositol-4,5-bisphosphate 3-kinase (PI3K)/protein kinase B (PKB/AKT)/mechanistic target of rapamycin (mTOR) pathway and metabolic central regulator-AMP activated kinases (AMPKs) are involved in neural development by regulating cell growth, proliferation, survival, and metabolism.^30,31^ Dysregulation of the apoptotic signal-regulating kinase 1 (ASK1)/p38 mitogen-activated protein kinase (MAPK) pathway and the abnormal activation of key signaling molecule-Ca^2+^/calmodulin (CaM)-dependent protein kinase II (CAMKII) in synaptic plasticity contributes to AD progression by impairing the long-term potentiation, triggering inflammation and cell apoptosis.^32^ Phosphorylation of α-syn at the Ser129 site is regulated by multiple kinases such as casein kinase II (CKII) and polo-like kinase (PLK),^33,34^ leading to abnormal aggregation of α-syn in Lewy-bodies of PD patients. Furthermore, an imbalance in the receptor-interacting Ser/Thr-protein kinase-1 (RIPK1),^35^ receptor of activated protein kinase C1,^36^ and leucine-rich repeat kinase 2 (LRRK2)^37^ kinase signaling cascades exacerbates the course of both PD and ALS.

Here, we summarize the extensive evidence linking protein kinases with neurodegenerative diseases, and the current progress of kinase inhibitors in clinical trials targeting neurodegenerative diseases. We also discussed the challenges and future directions of kinase-targeted therapeutic strategies for clinical applications. Taken together, understanding the mechanisms of how protein kinases participate in neurodegeneration still holds great promise and substantial opportunities for future kinase-directed drug development.

## Definition and classification of protein kinases

**Fig. 2:**
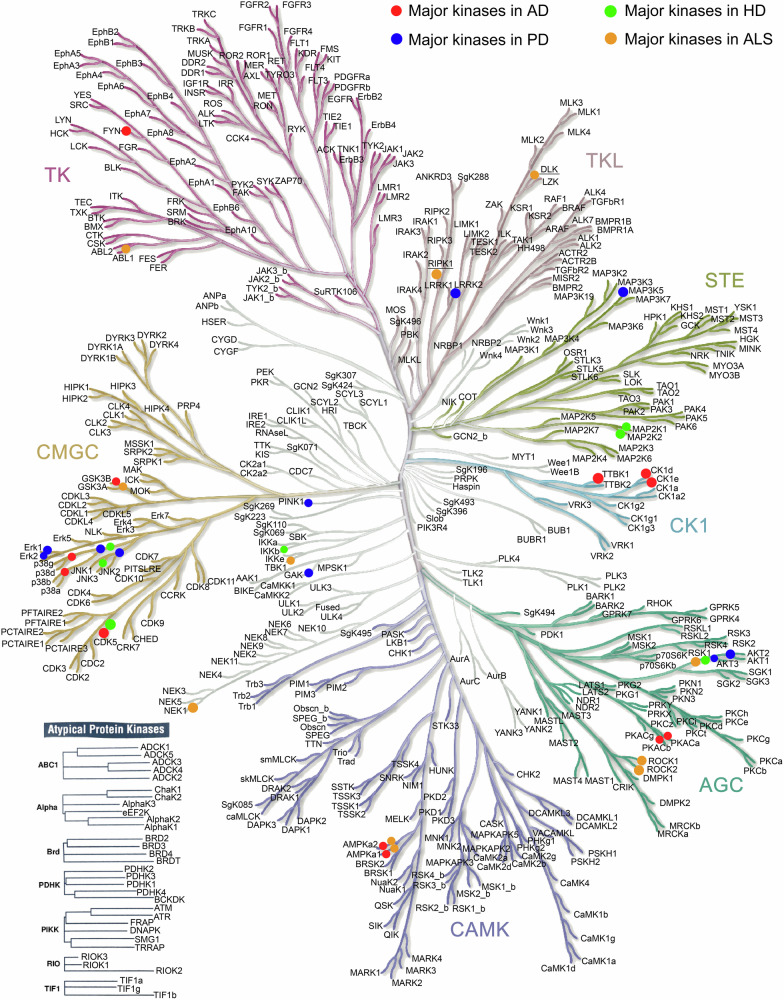
Kinase classification map. Based on the similarity of kinase domain sequences, 518 human protein kinases were divided into nine groups: tyrosine kinase group (TK group), tyrosine kinase-like group (TKL group), homologs of yeast Sterile 7, Sterile 11, Sterile 20 kinases group (STE group), containing PKA, PKG, PKC families group (AGC group), calcium/calmodulin-dependent protein kinase group (CAMK group), containing CDK, MAPK, GSK3, CLK families group (CMGC group), casein kinase 1 group (CK1 group), other group, atypical protein kinase group. Proteins of pathological significance for neurodegenerative diseases were highlighted in the figure using different colors as indicated in the figure. Figures generated using KinMap (http://kinhub.org/kinmap/index.html), and illustrations reproduced courtesy of Cell Signaling Technology, Inc. (www.cellsignal.com)

There are 478 typical (containing a eukaryotic protein kinase domain) kinases and 40 atypical kinases (exhibiting kinase activity without the eukaryotic protein kinase domain) identified to date. Based on the sequence similarity of protein kinase domains, the typical kinases can be further classified into eight groups,^38,39^ including the tyrosine kinases (TK) group, tyrosine kinase-like kinases (TKL) group, containing cyclin-dependent kinase (CDK), MAPK, glycogen synthase kinase (GSK), cell division cycle (CDC)-like kinase (CLK) families) (CMGC) group, homologs of yeast Sterile 7, Sterile 11, Sterile 20 kinases (STE) group, containing cAMP-dependent protein kinase (PKA), cGMP-dependent protein kinase (PKG), protein kinase C (PKC) families (AGC) group, calmodulin-dependent protein kinases (CAMK) group, casein kinase 1 (CK1) group (a small group of Ser/Thr protein kinases), and the other group. Additionally, 32 of 40 atypical protein kinases have also been identified as atypical protein kinases group (containing activity of bc1 complex kinase, alpha-protein kinase, bromodomain protein kinase, pyruvate dehydrogenase kinase, phosphoinositide-3-kinase-related kinase, right open kinase, transcription intermediary factor 1 kinase, etc), where the remaining atypical protein kinases have been further classified as protein kinase-like since they share the same structural fold as eukaryotic protein kinase.^40^ The kinase classification^38^ is illustrated in Fig. 2 and discussed in detail below.

### TK group

Tyrosine kinases catalyze the transfer of phosphate groups from ATP to the tyrosine residues of substrates. In the TK group, 58 kinases are receptor tyrosine kinases (RTKs) that can be divided into 20 families, composed of transmembrane receptors carrying tyrosine kinase domains in their intracellular segments; the other 32 kinases are non-receptor tyrosine kinases (non-RTKs) that can be divided into 10 families and they are consistently located in the cytoplasm.^41,42^

**Fig. 3:**
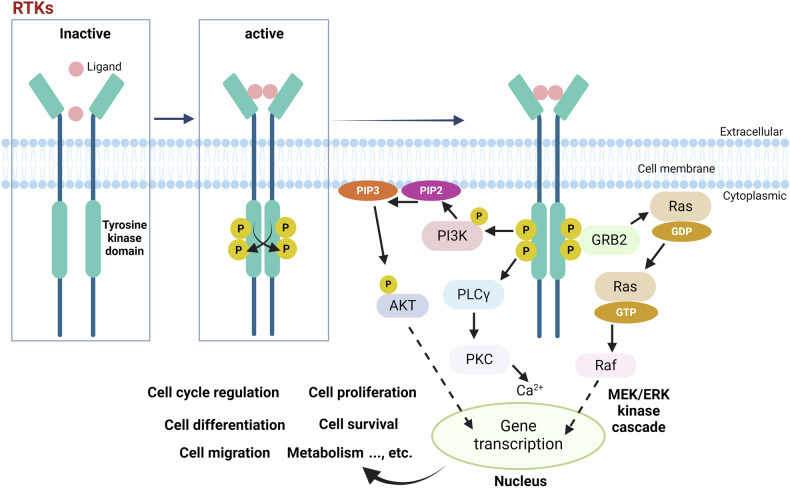
Typical RTKs-activated signaling cascades. Activation of the receptors (including growth factor receptors such as platelet-derived growth factor receptor (PDGFR), epidermal growth factor receptor (EGFR), vascular endothelial growth factor (VEGFR), and fibroblast growth factor receptor (FGFR), as well as tyrosine kinase receptor B (TrkB) and insulin-like growth factor 1 (IGF-1R)) by the corresponding cytokines, growth factors, and hormones induces autophosphorylation of their receptor tyrosine residues within the cell, thereby further amplifying the kinase activity, exposing the tyrosine phosphorylation docking sites, allowing them to be recognized by cytoplasmic proteins with Src homology 2 domain (SH2) or phosphotyrosine-binding (PTB) domains. Activated RTKs are able to recruit various signaling molecules and initiate downstream pathways. One major pathway involves the activation of phosphatidylinositol-3 kinase (PI3K), which converts PIP2 to PIP3, thereby activating AKT. Another pathway involves phospholipase C gamma (PLCγ), which, upon phosphorylation, stimulates protein kinase C (PKC) activity and mobilizes intracellular calcium (Ca^2+^). Concurrently, the RTK signaling cascade engages the Ras-Raf-MEK-ERK pathway via GRB2. Collectively, these pathways regulate diverse cellular processes, including cell cycle progression, proliferation, differentiation, survival, migration, metabolism, and other key functions. This figure was created with BioRender.com

Growth factor receptors such as platelet-derived growth factor receptor (PDGFR), epidermal growth factor receptor (EGFR), vascular endothelial growth factor (VEGFR), and fibroblast growth factor receptor (FGFR), along with tyrosine kinase receptor B (TrkB) and insulin-like growth factor 1 (IGF-1R), all fall within the category of RTKs. Activation of these receptors by corresponding cytokines, growth factors, and hormones induces auto-phosphorylation of intracellular auto-receptor tyrosine residues, leading to further amplification of the kinase activity and exposure of the docking sites for tyrosine phosphorylation which allows its recognition by cytoplasmic proteins containing Src homology 2 domain (SH2) or phosphotyrosine-binding (PTB) domains, and eventually triggering the activation of various downstream signaling cascades^43^ (Fig. 3). PI3K/AKT and Ras/Raf extracellular regulated protein kinases 1/2 (ERK1/2) pathways, typical signaling cascades activated by RTKs, are responsible for the regulation of cell cycle, cell survival, and cell proliferation. For instance, brain-derived neurotrophic factor (BDNF) improves the neuronal survival, plasticity, and function by activating the TrkB-PI3K/AKT signaling,^44^ and activation of IGF1-MEK/ERK and IGF1-PI3K/AKT signal transduction cascades modulates neurogenesis in the brain.^45,46^

**Fig. 4:**
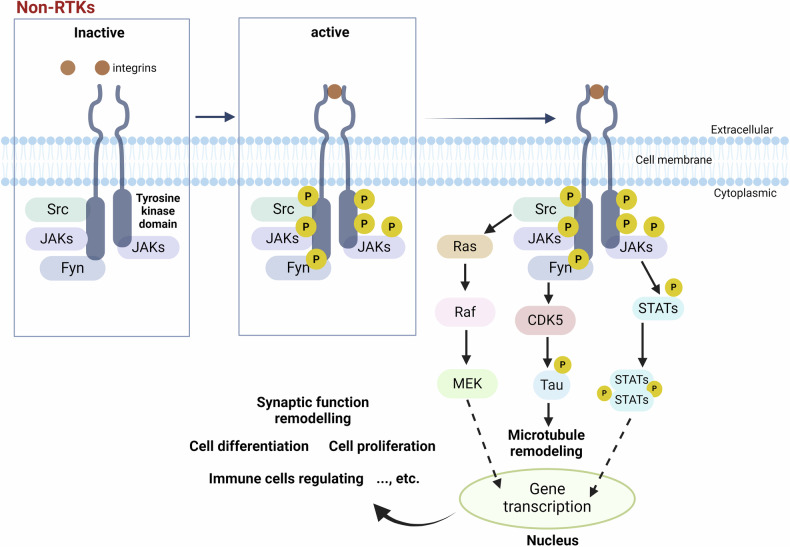
Typical non-RTKs activated signaling cascades. In addition to the conserved kinase domain, non-RTKs also have a variable number of protein domains (e.g., SH2 or SH3 domains responsible for binding to other signaling molecules). Typical non-RTKs consist of the Src family (including Src, Fyn, Lyn, Lck), the Abelson tyrosine kinase (Abl) family (including Abl1, Abl2), and the Janus kinase (JAK) family (including JAK1, JAK2, JAK3, TYK2). The Src-mediated Ras-Raf-MEK pathway leads to transcriptional regulation in the nucleus, impacting cellular functions. Simultaneously, Fyn activates cyclin-dependent kinase 5 (CDK5), which modifies tau protein to facilitate microtubule remodeling. The activation of the JAK-STAT pathway allows STATs to translocate to the nucleus and directly regulate gene transcription. The regulation of non-RTK signal transduction pathways is closely related to synaptic function remodeling, neuronal excitability regulation, immune regulation, cell proliferation, etc. This figure was created with BioRender.com

Typical non-RTKs consist of the Src family (including Src, Fyn, Lyn, Lck), the Abelson tyrosine kinase (Abl) family (including Abl1, Abl2), and the Janus kinase (JAK) family (including JAK1, JAK2, JAK3, TYK2). Structurally, non-RTKs have a variable number of protein domains (e.g., SH2 or SH3 domains responsible for binding to other signaling molecules) in addition to the conserved kinase domains.^41,42^ Representative diagram of the non-RTK signal transduction pathway is depicted in Fig. 4. Src and Fyn are ubiquitously expressed in various tissues, while Lyn and Lck expressions are more tissue-specific in the nerves, liver, adipose, and lymphoid tissue. Functionally, Src family kinases often act as signaling mediators, for example, Src phosphorylates and upregulates N-methyl-D-aspartate receptors (NMDAR) functioning at postsynapses, resulting in an aberrant influx of Ca^2+^ that eventually leads to neuronal death in the brain.^47–50^ Fyn regulates excitatory or inhibitory neurotransmission by interacting with various effector proteins such as CDK5, tau, Dab1, mGluR1, and GluN2B, and Fyn-related modulations are tightly associated with learning and memory processes (reviewed elsewhere^51^). Fyn is highly similar to other Src members, and the typical motifs of Fyn consist of SH4, SH3, SH2, and SH1 domains, with the catalytic SH1 domain showing TK activity highly conserved among Src family members.^51,52^ The phosphorylation status of Tyr420 and Tyr531 in Fyn, by tyrosine kinases and phosphatase, are critical for the precise regulation of its kinase activity. Both Csk-mediated phosphorylation of Fyn at Tyr531 and striatal enriched phosphatase (STEP) mediated dephosphorylation of Fyn at Tyr420 lead to Fyn inactivation.^53,54^ Conversely, the dephosphorylated Tyr531 epitope allows the opening of the inactive conformation, enabling the exposure of SH2 and SH3 domains for protein interaction, followed by full activation of the catalytic loop in the SH1 domain.

Abl localizes in neuronal axons of the central nervous system (CNS) and regulates the axonal growth. In *Drosophila melanogaster*, an impaired signaling network mediated by Abl mutation interrupts the dynamics of actin cytoskeleton in the neuronal growth cones.^55^ c-Abl has been shown to phosphorylate tau protein at Tyr394, and both Fyn and c-Abl are critical regulators in the neurodegenerations involving tau lesions.^56^ The JAK phosphorylates its substrate, signal transducer and activator of transcription (STAT), causing STAT to dimerize, which then translocates into the nucleus and triggers the expression of target genes, thereby promoting cytokine-mediated cellular activation. The failure of this signaling pathway may disrupt the normal immune responses and induce pathological effects in diseases.^57–60^ Different from JAK3 which is expressed exclusively in the bone marrow and lymphatic system as well as endothelial cells and vascular smooth muscle cells, other JAK family members are expressed in almost all tissues.

### TKL group

TKL generally lack the TK-specific motifs of the TK group, but they share similar amino acid sequences with TKs, such as the catalytic motif (His-Arg-Asp) in the kinase domain, and act as a Ser/Thr kinase in biochemical processes.^61^ LRRK2 belongs to the TKL group with GTPase and Ser/Thr kinase activities.^62^ Multi-domains in LRRK2 contain an armadillo repeat motif (ARM), an ankyrin repeat (ANK), a leucine-rich repeat (LRR), a Ras-of-complex (ROC)-C-terminal of Roc (COR) domain, a Ser/Thr kinase (KIN) domain and a WD40 domain. The ROC-COR and KIN domain are responsible for the GTPase activity and kinase activity, respectively. Mutations at specific sites within these domains may alter cellular processes such as vesicle trafficking, cytoskeletal dynamics, lysosomal function, and microglial responses.^37^ LRRK2 forms a monomer or dimer under physiological conditions, and generates a filamentous structure through microtubule-dependent aggregation in a pathological state. Recently, the cryo-EM structure of full-length human LRRK2 shows that the monomer adopts an elongated conformation similar to the letter ‘J’.^63^ The interaction of LRRK2 and Dlp1/ dynamin-related protein 1 regulates mitochondrial dynamics through the kinase activity of LRRK2 in neurons,^64^ and its activation promotes mitochondrial fragmentation in microglia.^65,66^

RIPK1, a key mediator of apoptotic and necrotic cell death and inflammatory pathways, belongs to the TLK group. Structurally, RIPK1 consists of an N-terminal kinase domain, an intermediate domain, and a C-terminal death domain (DD). The intermediate domain contains a receptor-interacting protein homotypic interaction motif (RHIM), which mediates the formation of amyloid proteins, and is involved in the interaction of RIPK1 with other RHIM-containing proteins such as RIPK3, TIR-domain-containing adaptor-inducing IFNβ, and Z-DNA binding protein 1.^67^ The C-terminal DD interacts with other proteins containing C-terminal DD (such as tumor necrosis factor (TNF)-α receptor 1 and FAS-associated death domain protein) to mediate their homodimerization, thereby promoting the activation of the N-terminal kinase domain.^68^ In the TNF signaling-mediated apoptosis and necroptosis pathway, RIPK1 assembles with TNFR1-associated death domain protein, TNFR-associated factor 2, and multiple E3 ubiquitin ligases (cellular inhibitor of apoptosis protein 1/2, linear ubiquitin chain assembly complex) into a large receptor-bound signaling complex I, mediating the first step of TNF signaling.^69^ When downstream nuclear factor-kappaB (NF-κB) activation is inhibited, a cytoplasmic complex called complex IIa can be formed, which mediates caspase-8 activation, RIPK1 cleavage, and RIPK1-independent apoptosis. While caspase-8 activation is blocked, RIPK1 C-terminal DD dimerization leads to RIPK1 activation and the formation of complex IIb (including FAS-associated death domain protein, caspase-8, RIPK1, RIPK3, and mixed lineage kinase domain-like, of which RIPK1 activity is required for the formation of complex IIb).^70^ Complex II is a downstream mediator after the first step of TNF signaling. The transmission of its downstream signal is regulated by the activity of caspase-8 and RIPK3, leading to apoptosis, necroptosis, and increased expression of inflammatory genes.^70^ Deletion and kinase-inactivating knock-in mutations of the *RIPK3* (including D138N, K45A, K584R, D161N) exhibit resistance to inflammatory and neurodegenerative processes.^68,71–74^

Dual leucine zipper kinase (DLK) is a Ser/Thr protein kinase in the TLK group. The number of amino acids that make up mouse and human DLK is 888 and 859, respectively. DLK has four characteristic domains: kinase domain, leucine zipper domain, glycine-serine-proline rich domain, and glycine-proline rich domain.^75^ The human DLK kinase domain consists of 127-375 amino acid residues, and its activity is regulated by the dimerization of DLK mediated by the leucine zipper chain domain.^76^ DLK binds to the c-Jun N-terminal kinase (JNK) interacting protein (JIP) through its N-terminal region, activating transcription factors downstream of the mitogen-activated protein kinase kinase 7/JNK signaling pathway (including STAT3, transcription activator 2, and myocyte-specific enhancer factor 2A, etc.), causing axon regeneration to respond to cell death signals caused by axon damage.^77,78^ DLK maintains the establishment of axonal bundles originating from pyramidal neurons in the cerebral neocortex, and mice deficient in DLK exhibit defects in axonal growth and neuronal migration.^79^

Interleukin-1 receptor associated kinase-M, specifically expressed in microglia,^80^ downregulates the toll-like receptor 4-myeloid differentiation primary response gene 88 signaling, which leads to the differentiation of microglia to a neuroprotective and anti-inflammatory M-phenotype, ultimately preventing the pathogenesis of experimental autoimmune encephalitis.^81,82^ Furthermore, two transmembrane Ser/Thr kinase receptors, Transforming growth factor-β receptor 1 and 2, transmit transforming growth factor-β signaling to intracellular mediators and contribute to neurogenesis, dentin regeneration, and carcinogenesis.^83–86^

### CMGC group

Major kinases in the CMGC group include CDK, MAPK, glycogen synthase kinase 3 (GSK3), and CLK. Among them, CDKs and MAPKs are two of the largest and most well-studied CMGC kinases.^87^

**Fig. 5:**
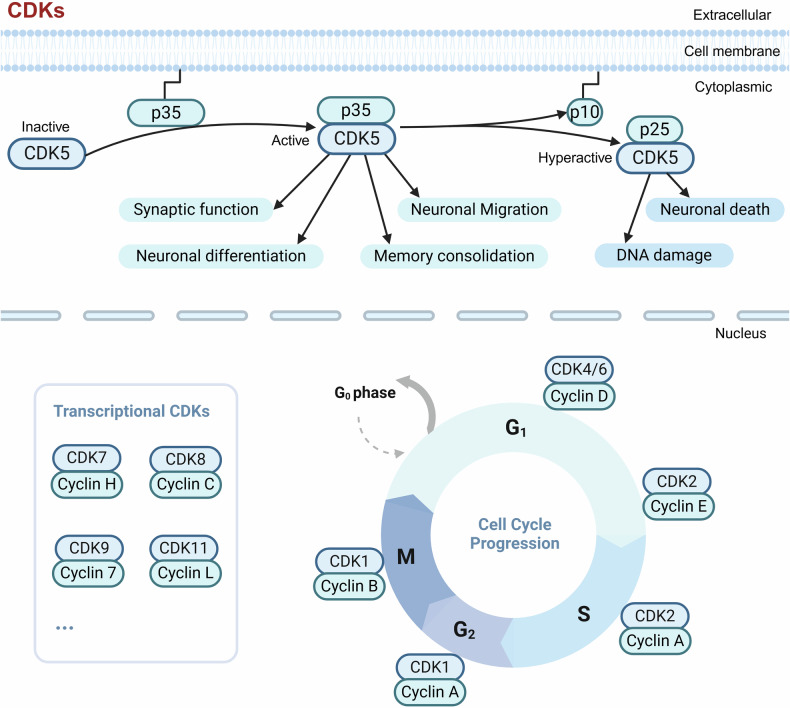
CDK family and functions. In mammals, the CDK family can be divided into two categories according to their functions: cell cycle-related CDKs (such as CDK1, CDK4, and CDK5) and transcription-related CDKs (CDK7, CDK8, CDK9, CDK11, and CDK20). In the first category, CDK1, CDK2, and CDK4/6 are located in the cell nucleus and combine with CycA/E, CycA/B, and CycD, respectively, to regulate the transformation of different cell cycle stages; while CDK5 is located in the cytoplasm in cells, is mainly active in post-mitotic neurons, and participates in neuronal differentiation, migration, synaptic function, and memory consolidation. Unlike classical CDKs, CDK5 is not activated by cyclins. Instead, it is primarily activated by its neuron-specific cofactor, p35, a regulatory protein that binds to CDK5 and induces a conformational change, enabling its catalytic activity, while cleavage of p35 to p25 under pathological conditions such as oxidative stress, calcium dysregulation, or neurotoxic insults, results in the overactivation of CDK5, driving neurotoxic processes. This figure was created with BioRender.com

CDKs are originally identified to regulate the cell cycle. In mammals, the CDK family can be divided into 2 categories, functionally as cell cycle-related CDKs (e.g., CDK1, CDK4, and CDK5), and transcriptional CDKs (CDK7, CDK8, CDK9, CDK11, and CDK20). Within the first category, CDK1, CDK2, and CDK4/6 are located in the nucleus and binds with CycA/E, CycA/B, and CycD, respectively, to regulate the transformation of cell cycle stages (Fig. 5). However, CDK5 located in the cytoplasm, is mainly active in post-mitotic neurons,^88^ and participates in neuronal differentiation, migration, synaptic function, and memory consolidation. CDK5 affects synaptic plasticity and memory formation by directly phosphorylating relevant substrates and interacting proteins. For instance, postsynaptic density protein-95 (PSD95), NMDAR, dopamine and adenosine 3’5’-monophosphate-regulated phospho-protein 32 kDa, and dopamine D2 receptors are all substrates of CDK5 in the postsynaptic compartment.^89–92^ CDK5 can also regulate protein phosphatases PP1, TrkB (BDNF receptor), and PKA (reviewed elsewhere.^93^). Furthermore, CDK5 regulates the expression of receptor tyrosine-protein kinase erbB-3 and postsynaptic acetylcholine receptor by phosphorylating STAT3 at Ser727, thereby negatively regulating the formation of neuromuscular synapses.^94,95^ Under physiological conditions, CDK5 is also shown to maintain survival signals by regulating PI3K/AKT activity, and CDK5/p35 blocks neuronal apoptosis by inducing Bcl-2 expression through ERK activiation.^96^

All transcriptional CDKs, including CDK7, CDK8, CDK9, CDK11, and CDK20, are located in the nucleus. Among them, CDK7 and CDK9 bind to CycH and Cyc7 respectively to directly phosphorylate the C-terminal domain of RNA polymerase II, thereby regulating the transcription process involving CDK8-mediated complex. CDK11 binds to CycL to control transcription by modulating the phosphorylation of hormone receptors and associated regulators or splicing factors.^97^ In a recent study, CDK20 has been reported to regulate the Wnt and Keap1-Nrf2 signaling pathways to facilitate cell proliferation.^98^

MAPKs regulate diverse cellular processes and are broadly involved in cell fate determinations across all eukaryotic phyla. MAPK family members, including ERK, JNK, and p38 MAPK, regulate cell proliferation, differentiation, and apoptosis.^99^ The JNK signaling pathway regulates axonal regeneration, nervous system development, and neuronal degeneration after acute injury or in chronic neurodegenerative diseases.^100^ Specifically, in the nervous system, negative regulation of MAPK by enhanced activity of MAP kinase phosphatase 1 (MKP-1, also known as dual-specificity phosphatase 1) is neuroprotective,^101^ and inhibition of p38α/β-MAPK activity reduces the number of degenerated neurons in the brain with improved cognitive function.^102^

GSK3 comprises two isoforms α and β, which share about 85% amino acid sequence homology in humans, and these two isoforms adopt similar secondary structures.^103^ GSK3α is only significantly expressed in the cortex, hippocampus, striatum, and cerebellum,^104^ whereas GSK3β is uniformly expressed in all brain regions. In physiological processes, GSK3α is localized in the cytoplasm, while truncated GSK3α lacking the N-terminal region accumulates in the nucleus. In contrast, GSK3β is more likely to be localized in the nucleus, especially in the context of cell proliferation and apoptosis.^105^ Functionally, GSK3α is generally associated with lifespan, mental state, behavior, and lipid metabolism, while GSK3β plays a crucial role in promoting neural development, the formation of neuronal polarity, and the maintenance of brain structure and function.^105^ Hence, the expression of GSK3α/GSK3β is strictly regulated in the spatiotemporal sequence. As a protein kinase, GSK3 can phosphorylate almost all downstream proteins with the S/T-X-X-X-S/T(P) motif, and is also dynamically regulated by multiple kinases including AKT, PKA, and PKC.^106^ Studies have confirmed that excessive activity of GSK3 is strongly correlated with neurodegenerative diseases such as AD, as described in detail below.

CLK family, relatively less studied, includes dual-specificity Tyr-regulated kinases (Dyrks) and Ser-Arg protein kinases,^87^ of which Dyrk1A functions in both of the cytoplasm and nucleus, interacts with histone acetyltransferase p300/CBP, and contributes to mental retardation and microcephaly.^107^ A global proteomic analysis of the human CMGC kinome complex provides extensive insights into resources and approaches for the analysis of CMGC kinases and human diseases,^87^ with information detail also available in the IntAct database (accession number: IM-17935, http://www.ebi.ac.uk/intact/).

### STE group

**Fig. 6:**
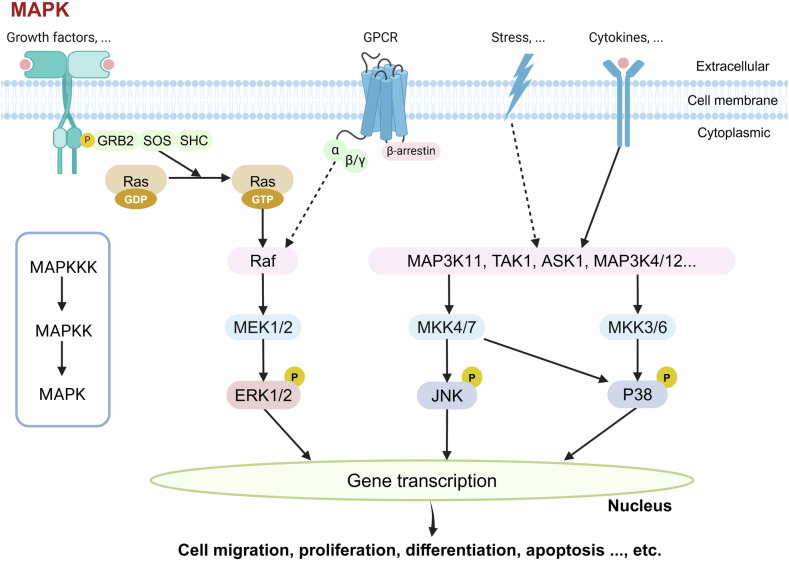
MAP3K-MAP2K-MAPK signaling pathway. In a typical MAPK cascade, MAP3K activates MAP2K (also known as MKK, MEK) by phosphorylating two conserved Ser/Thr residues in the activation loop, and the MAP2Ks directly phosphorylate MAPKs. The activation of MAPK cascades is initiated by various extracellular stimuli, including growth factors, G-protein-coupled receptor (GPCR) signaling, stress, and cytokines. Activated Ras recruits and activates Raf, which phosphorylates and activates downstream MEK1/2. MEK1/2 then phosphorylates ERK1/2, which translocates to the nucleus and regulates gene transcription. Stress signals (e.g., reactive oxygen species or osmotic stress) and cytokines activate distinct MAPKKKs, leading to the phosphorylation of different MAPKKs. MKK4/7 phosphorylates and activates JNK/p38 MAPK, while MKK3/6 phosphorylates p38 MAPK. The hierarchical organization of the MAPK pathways ensures signal specificity, playing a critical role in cell apoptosis, cell survival, and other cellular events. This figure was created with BioRender.com

STE kinase group consists of three main families, Sterile 7 (Ste7, also known as MAP2K), Sterile 11 (Ste11, also known as MAP3K), and Sterile 20 (Ste20, also known as MAP4K). After being activated sequentially, they activate the MAPK family (Fig. 6). The MAP2Ks directly phosphorylate MAPKs. In a typical MAPK cascade, MAP3K activates MAP2K by phosphorylating two conserved Ser/Thr residues in the activation loop, while MAP4K acts on MAP3K.^108^

In neurons, MAP4Ks serve as critical regulators of the DLK/JNK signaling by phosphorylating DLK, an axonal stress-responsive MAP3K, followed by translocation of JNK-dependent c-Jun signaling complex to the nucleus in response to stress.^100^ However, JNK is not the primary target for MAP4Ks in immune cells, and the latter regulates immune responses, related signaling, and inflammation activation through other targets.^109^ Indeed, activation of MAP3K/DLK leads to rapid cell death, whereas activation of MAP3K/leucine-zipper-bearing kinase (LZK) causes slow degeneration in cerebellar Purkinje cells in mouse models with conditional knockout or overexpression of *DLK* or *LZK*, and these two MAP3Ks independently induce JNK activation and caspase-mediated apoptosis. Therefore, precise control of DLK and LZK activation is essential for neuronal survival.^110^ Compounds targeting the MAP4K family exert neuroprotective effects, as suggested by that an exceptionally potent, blood-brain barrier (BBB)-penetrant and metabolically stable compound, prostetin/12k, has been screened out and demonstrates the potential to treat ALS.^111^

### AGC group

In the human genome, more than 63 protein kinases share the AGC group features, including 20 Ser/Thr protein kinases such as PKA, 3-phosphoinositide-dependent protein kinase 1 (PDK1), AKT, and Rho-associated coiled-coil containing kinase (ROCK), which can be divided into 14 families. Two other families, aurora kinase and PLK are most closely related to the AGC group.^112,113^

PKA is composed of four subunits, two regulatory subunits (types I and II) that bind to two catalytic subunits (α and β) to modulate a variety of cAMP-dependent cellular responses, such as pro-survival gene transcription, neuronal differentiation, and synaptic plasticity.^114,115^ The PKA catalytic subunit domain consists of a small N-terminal lobe containing a 5-strand β-sheet and an αC helix, and a large C-terminal lobe mainly composed of α-helix (AGC kinases usually have a second helix αB adjacent to the αC helix).^116,117^ Between the two lobes, a connected deep pocket serves as the ATP binding site,^118^ which is one of the major targets for drug development.^113^ These structural conformations represent a common model for understanding the structures of the entire superfamily. Moreover, AGC kinases have two regulatory phosphorylation sites (hydrophobic motif and turn motif/zipper phosphorylation site, respectively) in addition to the activation loop that is shared by the diverse kinase groups.^113^

As a superfamily widely distributed, kinases in the AGC group broadly regulate physiological processes.^119–122^ Knockin of *PDK1*^K465E/K465^ in mouse neurons causes inadequate phosphorylation of Thr308 of AKT (PDK1 substrate), incomplete phosphorylation and inactivation of proline-rich AKT substrate of 40 kDa and tuberous sclerosis complex 2, and reduced activation of the mechanistic target of rapamycin complex 1 (mTORC1), followed by declined protein synthesis of brain-specific kinase and insufficient neuronal differentiation, giving rise to reduced brain size in mouse.^123^ Mutations in the docking site of AKT-independent PDK1 substrate cause microcephaly and abnormal brain morphogenesis in the developing mouse brain, leading to cognitive impairment and disruptive behavior in adult mice.^124^ Furthermore, in cultured primary rat hippocampal neurons, increased ROCK2 induces dendritic spine loss via the serine and threonine kinase LIM domain kinase 1 whereas administration of SR7826, an inhibitor of LIM domain kinase 1, rescues ROCK2-mediated dendritic spines loss and morphological distortion.^125^

### CAMK group

CaMKs are critical Ca^2+^ sensors that convert glutamatergic activation into synaptic plasticity during the formation of learning and memory.^126^ CaMKII is one of the Ca^2+^/CaM-regulated kinases and is evolutionarily closer to the phosphorylase kinase, whose intrinsic regulatory δ-subunit is recognized to be CaM.^127,128^ Originally, CaMKI-IV were named according to the elution order of brain extract separated with a fractionating column.^129,130^ Later studies indicated that this nomenclature was not rational, i.e., CaMKI/IV and CaMKII were members of related sister groups, but CaMKIII (known as eukaryotic elongation factor 2 kinase, eEF2) is now classified as atypical kinase instead of CaMKs.^3,130^ Thus, although the role of CaM regulation seems to be the shared feature of the CaMK family, not all CaM-regulated kinases are CaMKs. For example, death-associated protein kinase (DAPK) 1 and 2 (DAPK2, also known as DRP-1) can be activated by CaM, but DAPK3 (also known as zipper-interacting protein kinase, ZIPK) cannot respond to CaM due to a lack of CaM binding site.^130,131^ In addition, the CaMK subfamily that lacks CaM regulation also includes AMPKs and 90 kDa ribosomal S6 kinases.^130^ As a key regulator of cellular energy homeostasis, AMPK regulates a diverse range of physiopathological processes, especially in the homeostasis of mitochondrial function and autophagy.^132–134^ ATP/Ca^2+^ regulates liver kinase B1 phosphorylates AMPKα subunit at Thr172 and activates the main metabolic AMPK signaling, thereby exhibiting a protective effect on neural energy metabolism and autophagic degradation.^135,136^

As a result of the enriched presence of CaM in the synapses, the influx of Ca^2+^ through NMDA receptors leads to the formation of Ca^2+^/CaM complexes that activate CaMKs, causing induction of persistent synaptic plasticity via calcium signaling.^126,137,138^ CaMKII is profoundly abundant in the brain and has a remarkable biochemical profile as a multifunctional kinase.^126,139^ Among the 12 subunits of CaMKII, αCaMKII, and βCaMKII are the most abundant subunits in the brain, and the former is exclusively expressed in glutamatergic neurons, while the latter is present in both excitatory and inhibitory neurons.^140^ Upon Ca^2+^/CaM binding, attachment of αCaMKII to F-actin is attenuated, which dissociates CaMKII from F-actin and modulates actin polymerization to form synaptic morphologies.^126,141^ The αCaMKII-dependent CaMKII function is illustrated by that autophosphorylated αCaMKII at Thr286 prolongs the activity of CaMKII at synapses after Ca^2+^ stimulation, leading to the transport of glutamate receptors to the postsynaptic density and subsequent enhanced synaptic transmission.^138,142^ Therefore, Ca^2+^/CaMKII contributes to synaptic transmission and is required for long-term potentiation (LTP) maintenance.^143^ Furthermore, as a Ca^2+^/CaM-dependent Ser/Thr kinase, DAPK1 overexpression and phosphorylation at Thr231, Ser262, and Ser396 sites are implicated in various neurological disorders, such as AD,^144,145^ PD,^146^ and stroke.^147^

### CK1 group

CK1 is so named because of its ability to phosphorylate milk protein casein in vitro, and the CK1 group includes the CK1 isoform, vaccinia-related kinase, and tau tubulin kinase 1/2 members.^3,148,149^ To date, seven mammalian CK1 isoforms have been grouped, including α, α-like, γ1, γ2, γ3, δ, and ε, due to the high homology among their N-terminal kinase domains.^150^ The α, α-like, δ, and ε isoforms have higher sequence similarity in the kinase domain than the γ isoform.^151,152^ Furthermore, CK1 isoform distribution is specific in organs and cells.^153,154^ For example, both CK1δ and CK1ε are mainly expressed in the brain.^155^ Activation of these two isoforms is regulated by the inhibitory autophosphorylation in the C-terminal region,^156^ and is associated with brain activities such as circadian rhythm,^157^ dopamine signaling,^158^ and neurotransmission.^159^ CK1 phosphorylates β-catenin at Ser45 and primes subsequent sequential phosphorylation at Thr41, Ser37, and Ser33 by GSK3.^160–162^ CK1 isoforms regulate the Wnt pathway via antagonistic roles in the signaling cascade,^150^ as well as p53 signaling,^163^ Hippo signaling,^164,165^ and Hedgehog signaling.^166,167^ Reduced phosphorylation of LRRK2 mediated by CK1 triggers the degradation of LRRK2, thereby disrupting the LRRK2 homeostasis.^168^ Both tau tubulin kinase (TTBK) 1 and TTBK2 belong to the CK1 superfamily, and can phosphorylate microtubule-associated proteins at 10 different residues to regulate neuronal function.^3,169,170^

### Other groups

Within this group, nearly all members are Ser/Thr kinases with distinct sequence homology, and almost all are involved in cell division. This group consists of 30 families (such as Aurora, PLK, cell division cycle 7, never in mitosis gene A (NIMA)-related kinase (NEK), CaM-dependent protein kinase kinase (CAMKK), IkappaB kinase (IKK), TBC1-domain containing kinase) and 2 subfamilies (including general control nonrepressible 2 and pancreatic eIF2alpha kinase), and many of which involved in neuronal processes will be discussed below.

The NEK family consists of 11 kinases named NEK1 to NEK11. Among them, NEK2/6/7/9 promotes the establishment of a microtubule-based mitotic spindle, while NEK1/10/11 is related to DNA damage response.^171^ All 11 human NEKs contain a His-Arg-Asp (HRD) motif within the catalytic domain as well as sites for activation modification at Ser or Thr residues within the activation loop. Compared with the conserved catalytic domain, the C-terminal regions of the 11 NEK species differ greatly in length, sequence, and domain organization.^172^ The diversities in these domains or motifs may explain the selectivity of NEKs during the cell cycle progression and differentiation processes.

The IKK family includes typical and atypical IKK kinases. The typical IKK includes IKKα, IKKβ, and IKKγ (also known as NEMO), and atypical IKK includes IKKε and TANK-binding kinase 1 (TBK1). The typical IKKα-IKKβ-IKKγ complex serves as the signal integration center for NF-κBactivation and consists of two Ser/Thr kinases (IKKα and IKKβ) and the regulatory subunit IKKγ. There is about 50% high sequence homology between IKKα and IKKβ, both of which contain an N-terminal kinase domain, dimerization domain, and C-terminal IKKγ binding domain (NBD). The kinase activity of IKKα mainly depends on the phosphorylation of Lys44/Ser176/Ser180, while IKKβ activation mainly depends on the phosphorylation of Ser177/181.^173^ The complex catalyzes the phosphorylation of inhibitor-κB and p65 proteins and other substrates, and mediates downstream immune and inflammatory responses, cell proliferation and differentiation, autophagy and apoptosis physiological processes.^174^ Dysregulation of these physiological functions is associated with a variety of diseases (such as cancer, neurodegenerative diseases, and heart disease).

The structure of atypical IKK, TBK1, includes an N-terminal kinase domain (KD), ubiquitin-like domain (ULD), α-helical scaffold dimerization domain (SDD) and C-terminal adapter binding domain (CTD). The phosphorylation site of TBK1 is Ser172 on the KD activation loop,^175,176^ and TBK1 controls selective autophagy of damaged mitochondria by phosphorylating sequestosome 1 (p62) and optineurin (OPTN) autophagy receptors, while mutations in *TBK1* lead to impairment of the selective autophagy pathway in protein aggregation.^177^ In the innate immune system, activation of TBK1 promotes the release of type I interferon (IFN) through the nuclear translocation of phosphorylated IFN regulatory factors 3 and 7.^178^ TBK1 is also involved in the mitosis of cancer cells to regulate cell survival by mediating PLK1 phosphorylation.^179^

CAMKK is the upstream kinase of CAMK and is responsible for CAMK activation. CAMKK belongs to the other group since its sequence differs from the CAMK family, however, both CAMKKα and CAMKKβ are highly homologous and compatible. They catalyze the phosphorylation of Thr177 of CAMKI and Thr196 of CAMKIV, activating CAMKI and CAMKII to regulate cell energy metabolism, proliferation, differentiation, and survival.^180^

## Kinases in neurodegenerative diseases

### AD

Dementia is an age-related, progressive, and irreversible neurodegenerative disorder, and is characterized by cognitive and memory impairment, compromised executive function, and difficulties in performing daily activities.^181,182^ AD is the most common form of dementia in the elderly, with more than 50 million people suffering from AD or AD-related dementia worldwide.^181^ To date, the U.S. Food and Drug Administration (FDA) has approved a variety of prescription medications for the relief of AD symptoms, including acetylcholinesterase (AchE) inhibitors (donepezil, galantamine, rivastigmine),^183^ NMDAR antagonists (memantine),^184^ and anti-Aβ antibodies (Aduhelm, Leqembi) that were recently approved through accelerated approval in 2021^185^ and 2023.^9^ In addition, Donanemab, developed by Eli Lilly, is also a monoclonal antibody that binds to Aβ subtype N3pG.^186^ and is approved by the FDA in 2024. Although these drugs may relieve the cognitive and behavioral symptoms in AD patients, they do not cure the disease. Therefore, an in-depth investigation of AD pathogenesis will be of great significance for targeted drug development.

Pathological features of AD include neurofibrillary tangles (NFTs, composed by tau aggregation), extracellular senile plaques (SPs, composed by Aβ aggregation), gliosis, and dystrophic neurites, accompanied by cerebrovascular amyloidosis, neuronal loss, metal dysregulation, and synaptic alterations.^187–194^ Aβ is generated by the amyloidogenic processing of amyloid precursor protein (APP).^195^ The cleavage of APP involves three proteolytic secretases including α-secretase (ADAM9, ADAM10, ADAM17), β-secretase (BACE1/2), and γ-secretase (composed of at least four core components including presenilins1 and 2, nicastrin, anterior pharynx defective 1, and presenilin enhancer 2).^195^ In the amyloidogenic pathway, APP is initially cleaved by BACE1 to release the sAPPβ ectodomain, and the 99 amino acids C-terminus of APP (C99) is further cleaved at various sites by γ-secretase,^195^ resulting in Aβ peptides in different length (including Aβ37-43), which tend to accumulate in the AD brain. Aβ40 and Aβ42 are the two major Aβ species, but neurotoxic Aβ42 is the main component of amyloid plaques.^196^ In addition to Aβ plaque neuropathology, several other neurochemical abnormalities, including elemental signatures of iron, copper, zinc, and selenium, have been widely validated in AD brains.^197^

Tau undergoes a series of post-translational modifications including hyperphosphorylation, acetylation, carboxy-terminal truncation, O-GlcNAcylation, and N-glycosylation before NFTs are formed.^198,199^ In AD, early hyperphosphorylation of tau disrupts the association between tau and the microtubules, prompts the mislocalization of tau from axons to the somatodendritic compartment, resulting in increasing levels of tau in the somatic domain where p-tau422 epitope turns positive,^198,200^ whereas other epitopes such as p-tau396 only become more prominent later in the disease.^201^ Various kinases such as CDK5 and CDK5 activator 1, truncated form of a CDK regulator p25,^202^ GSK3β, and phosphorylated JNK are upregulated in brain tissue from AD patients^203,204^; hyperactive GSK3β induces inflammation through NF-κB, impairs axonal transport, and promotes apoptosis,^198^ and these findings together suggest that increased activity of kinases may be responsible for tau pathology.

#### Major kinases in AD

**Fig. 7:**
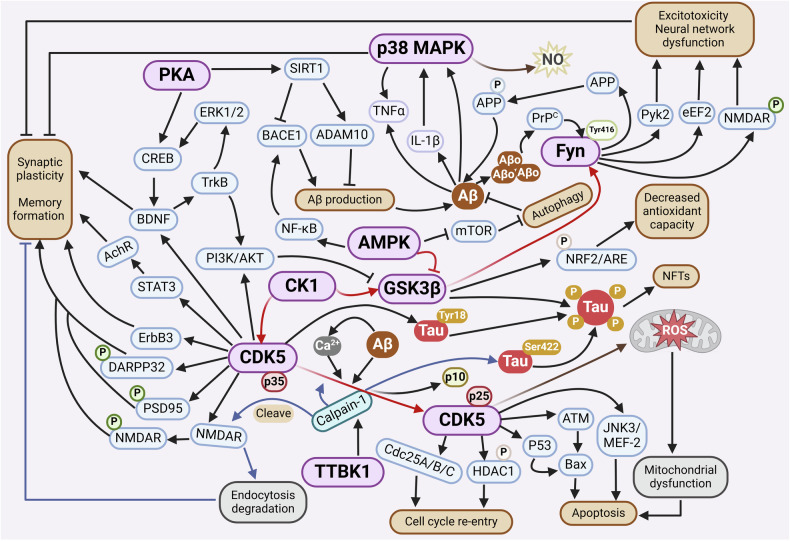
Schematic description of kinase signaling pathways in Alzheimer’s disease. GSK3β, as one of the main kinases involved in tau phosphorylation, adds a phosphate group to the Thr231 site on tau. This process triggers tau oligomerization, NFTs formation, and participates in the regulation of the Nrf2-ARE pathway by phosphorylating Nrf2 Ser334-338 residues, thereby reducing the antioxidant capacity. The CDK5/p35 complex plays a critical role in maintaining synaptic function by modulating STAT3, synaptic components including PSD95 and DARPP32, ErbB3, BDNF/TrkB, or other regulators, while in AD, the CDK5/p35 is cleaved by Ca^2+^, Aβ, and calpain-1, giving rise to the abnormally hyperactive CDK5/p25 variant, promoting the pathway of cellular apoptosis, reentry into the cell cycle, and mitochondrial dysfunction. The phosphorylation of p38 MAPK exacerbates oxidative stress, decreases synaptic plasticity, and increases the release of inflammatory factors. Meanwhile, overactivated Fyn contributes to the phosphorylation of APP at Tyr682, leading to increased generation of intracellular Aβ. Fyn phosphorylation at Tyr416 causes cellular toxicity and imbalances in neural network function by modulating NMDAR, Pyk2, and eEF2. TTBK1 activates CDK5 and triggers downstream signaling, promoting NMDAR internalization and imbalanced degradation of the neural network. On the other hand, it triggers the phosphorylation of tau protein at Ser422 via calpain-1, exacerbating tau aggregation. In the progression of AD, the reduced activity of AMPK increases the phosphorylation level of mTOR. This, in turn, hinders autophagy processes while concurrently enhancing Aβ generation. The downregulation of PKA expression in AD pathology leads to decreased activation of both SIRT1 and CREB, increasing Aβ production and synaptic plasticity vulnerability. CK1 abnormalities not only regulate the transmission of their inherent signals but also wield regulatory influence over the downstream signaling pathways of crucial kinases such as GSK3β and CDK5. This intricate interplay between kinases forms an interconnected regulatory network that functions in AD. This figure was created with BioRender.com

We here summarized the major kinases that are involved in AD and the kinase regulatory network (Figs. 2 and 7). These kinases include GSK3β, CDK5, CK1, PKA, p38 MAPK, Fyn, TTBK1, and AMPK. The details of each kinase involved in AD pathogenesis will be discussed below.

##### GSK3β

As a ubiquitously expressed Ser/Thr kinase, GSK3β activity in the peripheral blood of AD patients is positively correlated with the degree of dementia.^205^ GSK3β mainly affects the pathology of AD, including Aβ formation, tau pathology, neuronal survival and apoptosis, oxidative stress, and neuroinflammation. During Aβ production, GSK3β promotes the phosphorylation of APP and aggravates the β-cleavage of APP,^206,207^ and also inhibits APP autophagic degradation by reducing lysosomal biogenesis, thereby increasing Aβ levels in AD animal and cell models.^105^ In NFT formation, GSK3β acts as one of the major tau kinases by specifically phosphorylating tau at Thr231, which accelerates tau dissociation from the microtubules, promoting tau oligomerization and NFT formation.^208^ GSK3β interacts with other kinases such as CDK5 to amplify the hyperphosphorylation of tau. Overactivation of GSK3β inhibits the activity of protein phosphatase 2A, resulting in the blockage of tau dephosphorylation and subsequent synaptic dysfunction.^209,210^ The impairment of the Wnt/β-catenin signaling pathway further aggravates the elevated GSK3β activity and promotes tau hyperphosphorylation, leading to abnormal neuronal structure and dysfunction in *PS1* knockout fibroblasts from and mutant *PS1* transgenic mice.^211–213^

GSK3β also inhibits BDNF-induced TrkB receptor endocytosis by directly phosphorylating mixed-lineage kinase 3 or phosphorylating dynamin1, impairing the activation of AKT signaling downstream of BDNF-TrkB, thereby reducing neuronal survival and promoting neuronal apoptosis both in vitro and in vivo.^214,215^ Additionally, reduced PI3K/AKT activation in AD patient brains^216^ increases GSK3β activity, which further phosphorylates Nrf2 at Ser sites within residues 334-338, leading to enhanced degradation of Nrf2, accumulated oxidative stress, and cognitive deterioration.^217^ GSK3β is also involved in AD-related neuroinflammation by mediating the phosphorylation of CCAAT/enhancer-binding protein δ to upregulate MCP-1, MMP3, and MMP1 expression in astrocytes, promoting microglia reactivity.^218^ Consistently, GSK3β transgenic mice exhibit hyperphosphorylated tau, increased Aβ accumulation, reactive gliosis, neuronal death, enhanced oxidative stress, and cognitive deficits, all of which can be reversed by GSK3β inhibitors.^217,219,220^ These results highlight GSK3β as a promising therapeutic target against AD.

##### CDK5

In AD, abnormally elevated CDK5 activity enhances the phosphorylation of its substrates such as APP, tau, and neurofilaments.^93^ Mechanistically, CDK5 is activated by its neuron-specific and membrane-localized activators p35 and p39, which are cleaved by calpain into p25 and p29, respectively. Upon increased Ca^2+^ concentrations, CDK5/p25 binding is more stable, leading to the hyperphosphorylation of multiple substrates of CDK5, thus promoting AD progression.^93,221^ Increased phosphorylation of APP (p-Thr668) by CDK5 and excessive Aβ accumulation were observed in p25 transgenic mice^222^; whereas Aβ aggregation in turn contributes to abnormal CDK5 activity, forming a toxic feedback loop that aggravates AD progression.^223–225^

There is abundant evidence that CDK5 upregulates GSK3β activity and promotes phosphorylation of tau and other substrates, and c-Abl tyrosine kinase is responsible for CDK5 activation in this process.^226,227^ CDK5/p25 enhances p53 activity by phosphorylating p53, and promoting p53-Bax induced neuronal apoptosis,^93,228^ a process that also involves CDK5/p25-regulated JNK3 pathway and regulation of MEF-2, a direct target of CDK5 in the nucleus.^229–231^ Dysfunction of CDK5 also causes mitochondrial dysfunction and ROS accumulation, promoting neuronal apoptosis.^232,233^

On the other hand, CDK5/p25 phosphorylates phosphatases such as cell division cycle 25A (cdc25A), cdc25B, and cdc25C, resulting in the upregulation of CDK1/2/4 and subsequent re-entry of the cell cycle, which ultimately leads to cell cycle-related neuronal death and degeneration.^234,235^ It was identified that the ubiquitination and degradation of p35 are regulated by hexokinase 2. The abnormal decrease of hexokinase 2 is conducive to the cleavage of p35 into p25, which leads to the overactivation of CDK5 and interferes with the degradation of β-catenin induced by GSK3β, thus inhibiting the activation of cell cycle machinery.^236^ In addition, CDK5/p25 interacts with and phosphorylates BM88 (also known as cell-cycle exit and neuronal differentiation 1), thereby promoting the degradation of BM88 and upregulating dynamin-related protein 1, leading to mitochondrial dysfunction and neuronal death in the brains of 5xFAD mice.^237^ Abnormal CDK5 also was reported to mediate Bcl2-associated athanogene-3 loss, leading to neuronal synaptic dysfunction in AD pathology.^238^

##### CK1

Overexpression of CK1ε in N2a cells stably expressing APP (N2A-APP695 cells) results in increased production of Aβ, and CK1 inhibitors block β-secretase cleavage of APP without affecting the Notch cleavage.^153^ Furthermore, in response to inflammation, CK1 is delivered from astrocytes to neurons through extracellular vesicles, which promotes translation and amyloidogenic processing of APP.^239^ Inhibition of the interaction between CK1δ and APP695 mitigates the pathogenic metabolism of APP.^240^ Expression of CK1δ is increased in the AD brain and correlated with tau pathology.^241^ Overexpressed CK1δ in N2a cells promoted the cytoplasmic aggregation of TDP-43 by phosphorylating Ser379, Ser403/404 and Ser409/410 residues of TDP-43, enhancing the instability and inhibiting the inclusion of exon 10 in tau mRNA.^242^ In addition, analysis of tau441 phosphorylation mutants showed that Ser68/Thr71, Ser214, and Ser289 of tau are specific substrates for CK1δ in vitro.^243^

Besides being directly involved in AD pathology, CK1 also acts as a priming kinase of other key AD-related kinases such as GSK3β.^244^ Pre-phosphorylation and activation of GSK3β by CK1 leads to elevated Aβ and tau hyperphosphorylation thereby aggravating AD pathology. CK1 also acts upstream of CDK5 and regulates its activity and downstream signals.^245,246^ Consequently, abnormal CK1 expression affects AD pathogenesis via direct CK1-associated signalings as well as other critical kinases in AD. Since current CK1 kinase inhibitors mainly take effect by targeting the ATP-binding sites, which are highly conserved among CK1 isoforms, these inhibitors are of less specificity and targeted drug design remains challenging for AD therapy.

##### PKA

In AD, calcium dysregulation leads to increased degradation of PKA subunits through excessive activation of calpain, which reduces PKA activity and subsequently downregulates the phosphorylation of transcription factor cAMP response element binding protein (CREB) and BDNF expression.^247–249^ Moreover, Aβ42-treatment interferes with BDNF-induced activation of other pro-survival Ser/Thr kinases such as PI3K/Akt and ERK.^250^

Additionally, overexpression of BACE1 interacts with cAMP at the transmembrane domain of BACE1, and downregulates cAMP levels, PKA activation, and CREB phosphorylation, thereby leading to cognitive impairment due to compromised cAMP/PKA/CREB signaling pathway in AD.^251^ Sirtuin 1 (SIRT1) exerts its deacetylase activity after phosphorylated by activated cAMP/PKA signaling,^252^ and upregulates ADAM10 and downregulates BACE1 to prevent Aβ production in APP-overexpressing cells and animal models.^253^ Taken together, increasing PKA activity to regulate downstream CREB and SIRT1 signaling may be of benefit for AD.

##### p38 MAPK

Phosphorylation of p38 MAPK is mediated by the MAP3K-MAP2K signaling cascade.^254^ Composed of α, β, γ, δ isoforms, p38 MAPKs are activated after dual phosphorylation of Tyr182 and Thr180 residues,^254,255^ resulting in adaptive responses through phosphorylation of p38-dependent kinases or transcription factors. In postmortem brain tissue of AD patients, intense phosphorylation of p38 MAPK was associated with Aβ-dependent inflammatory response and NFT formation, and the activation was shown to occur at an early stage in AD.^254,256^ In both glial cells and neurons, p38 MAPK-mediated signal transduction contributes to AD progression. For example, stimulation of microglia by Aβ fragments activates p38 MAPK signaling to promote the secretion of pro-inflammatory cytokines interleukin-1β and TNF-α, leading to chronic inflammation.^257,258^ Subsequently, interleukin-1β activates the p38 MAPK signaling pathway in neurons and astrocytes, resulting in the production of pro-inflammatory mediators such as nitric oxide (NO) and TNF-α in astrocytes.^259,260^ In neurons, activated p38 MAPK phosphorylates Nrf2, facilitates the interaction between Nrf2 and Keap1, and inhibits the nuclear translocation of Nrf2, leading to a decrease in ARE-dependent transcription of antioxidant enzymes.^217,261^

Furthermore, p38 MAPK activation in neurons alters synaptic plasticity and promotes tau phosphorylation.^262–264^ Several studies have shown that deficiency of p38α-MAPK in all myeloid cells prevents AD progression by increasing microglial clearance of Aβ and reducing pathological hyperphosphorylated tau.^265–267^ However, unlike p38α-MAPK, p38γ-MAPK is specifically localized to the post-synaptic density in neurons and mediates the phosphorylation of postsynaptic tau protein at Thr205.^268,269^ Phosphorylation of the Thr205 site promotes the removal of tau from the PSD95/NMDAR complex.^269,270^ The dissolution of the interaction between PSD95 and tau further uncouples tau from downstream factors such as Fyn and ERK, thereby inhibiting toxic signals downstream of NMDAR, such as Aβ excitotoxicity.^269,271^ These results suggest that p38 MAPK activation is significantly related to defective Aβ clearance, Aβ-induced neuroexcitotoxicity, and tau phosphorylation. However, specific subtypes may need to be considered when targeting p38 MAPK to alleviate AD pathology.

##### Fyn

Fyn is a member of the non-receptor tyrosine Src family. In AD pathogenesis, overactivation of Fyn promotes the phosphorylation of APP at Tyr682 and tau at Tyr18, leading to increased production of extracellular Aβ and intracellular NFTs formed by tau, respectively.^272^ Extracellular Aβ oligomer binding to cellular prion protein stimulates Fyn phosphorylation at Tyr420 and activates Fyn, triggering pathological signaling cascades and excitotoxicity caused by phosphorylation of NR2B/NMDAR and persistent activation of both eEF2 and protein tyrosine kinase 2β downstream of Fyn.^273,274^ In addition, hyperactivation of Fyn not only participates in tau pathology through direct interaction and phosphorylation, but also indirectly through the activation of p35/CDK5.^275,276^

Fyn is recruited to the postsynaptic NMDAR complex in a tau-dependent manner and mediates Aβ excitotoxicity in postsynaptic toxic signaling.^269,271,277^ In this recruitment pathway, tau interacts with Fyn through the proline-X-X-proline motif in its proline-rich region.^271,278^ Subsequently, the tau/Fyn complex interacts with the key scaffolding protein PSD95 to mediate excitotoxicity induced by Aβ or excess glutamate levels.^271,279^ On the contrary, phosphorylation of NR2B by Fyn at the Y1472 site can enhance the binding of PSD95 to NMDAR, thereby increasing the level of tau in the NMDAR complex.^269,280^ Therefore, Aβ-Fyn-tau closely contributes to AD pathogenesis, and Fyn inhibition (using the Src family kinase inhibitor, AZD0530) has been demonstrated to rescue spatial memory deficits and synaptic density loss in APP/PS1 mice, and rescue abnormalities in tau phosphorylation and deposition in 3xTg-AD mice,^271,277,281,282^ suggestive of Fyn as an attractive target for the treatment of AD.

##### TTBK1

TTBK1, a CNS-specific kinase that mediates tau phosphorylation and aggregation,^170^ is significantly upregulated in the frontal cortex of AD patients,^283^ and genetic variation in the *TTBK1* gene (single nucleotide polymorphism of rs2651206) is associated with late-onset AD in two cohorts of patients in China and Spain.^284,285^ TTBK1 directly phosphorylates tau at Ser422, an epitope present before NFT formation.^286^ Consistently, brain-permeable TTBK1 inhibitors are capable of reducing tau phosphorylation and alleviating AD pathology in both the mouse isoflurane-induced hypothermia model and rat developmental model.^287^

In addition, elevated levels of CDK5 co-activators p35 and p25, as well as increased calpain-1 activity and p35/CDK5 activity were found in TTBK1 transgenic mice,^149^ suggesting that TTBK1 activates CDK5 to trigger downstream signaling cascades. Both p35/CDK5 and calpain-1 (via proteinase activity^288^) closely regulate the turnover of NMDAR subunit NR2B on the membrane surface, and affect hippocampal spatial learning and LTP.^289,290^ Therefore, enhanced TTBK1 activity is assumed to upregulate p35/CDK5 and calpain-1 activity, which causes cognitive impairment via endocytosis and degradation of NMDAR. Moreover, p25 generated from the cleavage of p35 by calpain-1 acts as a more potential activator of CDK5 and subsequently forms the p25/CDK5 complex, resulting in augmented activity compared to the p35/CDK5 complex.^149,291^ All these events further disrupt the homeostasis of NMDAR and the balance of neural networks.

##### AMPK

AMPK belongs to the CAMK family, but lacks CaM regulation because it does not directly interact with AMPK due to the lack of CaM binding site. AMPK alleviates tau phosphorylation by reducing the activity of GSK3β, and activation of the AMPK signaling pathway induces SIRT1 activity to deacetylate tau followed by degradation of misfolded tau.^292,293^ However, in AD, disturbance of energy metabolism moderates AMPK activity in the brain, which on one hand increases mTOR phosphorylation and inhibits autophagy^294–297^; on the other hand reduces SIRT1 activation, resulting in augmented Aβ production and tau phosphorylation.^293,298^ Aβ42 oligomers can trigger synaptic loss through CAMKK2/AMPK-dependent modulation of mitochondrial fission and mitophagy in APP^Swe^/^Swe^-knockin human embryonic stem cell lines.^299^ Thus, AMPK, as a master regulator of energy-related signaling pathways, extensively participates in neurodegeneration.

### PD

PD, a chronic and progressive movement disorder, is the second most common neurodegenerative disease.^300,301^ The number of worldwide PD patients increased from ~2.5 million in 1990 to 6.1 million in 2016, and the incidence is predicted to rise to 13 million by 2040.^302,303^ The primary clinical symptom of PD is motor impairment, in addition to non-motor deficits including cognitive decline,^304,305^ depression, and pain, which greatly affect the quality of life of patients.^306^ The complexity of PD pathophysiology involves several functional abnormalities, such as mitochondrial, lysosomal, and synaptic dysfunctions, all of which may synergistically cause selective loss of dopaminergic neurons in the substantia nigra.^307,308^

The accumulation of α-syn in Lewy bodies and Lewy neurites, characterized by crowded organelles and lipid membranes, is the pathological hallmark of PD.^309^ Currently, inhibition of misfolded α-syn diffusion or promoting its clearance has been validated to mitigate the progression of PD.^310^ α-syn propagates from neuron to neuron and induces normal α-syn aggregation,^311^ a process that is closely related to the post-translational modifications (PTMs) of α-syn protein. Phosphorylation of α-syn occurs at 39, 87, 125, 129, 133, and 136 sites, and involves kinases such as CK, PLK, and G protein-coupled receptor kinase (GRK) families.^312,313^ Among all the phosphorylation sites, the pathological phosphorylation at Ser129 (pSer129) accounts for ~90% of the deposition in Lewy bodies.^314–317^ In vitro, α-syn phosphorylation at Ser129 leads to a higher tendency to form fibrils.^318^ Likewise, pSer129 α-syn induced by GRK5 facilitates α-syn aggregation in cells co-transfected with α-syn and GRK5.^319^

Selective loss of neurons in the SNpc is a typical pathological feature of PD comparable to the neuronal loss of the dorsal tier during normal aging.^320,321^ Although PD pathology involves the entire brain, specific types of neurons, especially the dopaminergic neurons of SNpc, are the most vulnerable to damage in PD. Given that the axons of SNpc dopaminergic neurons forming a huge branch network contain a higher density of mitochondria and therefore display a higher rate of oxidative phosphorylation than other neurons, these neurons will inevitably be more affected if mitochondrial dysfunction occurs.^322,323^ It was also found that iron and dopamine levels in the SNpc are significantly higher than in the adjacent ventral tegmental region, which is less susceptible to neuronal loss in PD.

The current first-line treatment for PD is L-3.4-dihydroxyphenylalanine (L-DOPA) supplementation, which is only a symptomatic treatment. Since the loss of dopaminergic neurons continues and the survival neurons may be exhausted by the treatment, L-DOPA works within 20 to 30 min of dosing, with maximum effects reaching about 1.5 h. Therefore, therapies for PD are expected to prevent ongoing neuronal death, and understanding the kinases involved in the pathomechanisms of PD is of particular significance.

#### Major kinases in PD

Approximately 3–5% of PD is attributed to monogenic PD. Mutations of *SNCA* (encoding α-syn), *LRRK2* (encoding LRRK2), *PRKN* (encoding Parkin), *PINK1* (encoding PINK1), and *GBA* genes have been identified as causal mutations of PD. For example, mutations in the *SNCA* cause familial PD in an autosomal dominant manner and increase the risk of sporadic PD.^324,325^ PD patients with *SNCA* mutations exhibit earlier onset age, more rapid progression of motor symptoms, and significant cognitive impairment.^326^

**Fig. 8:**
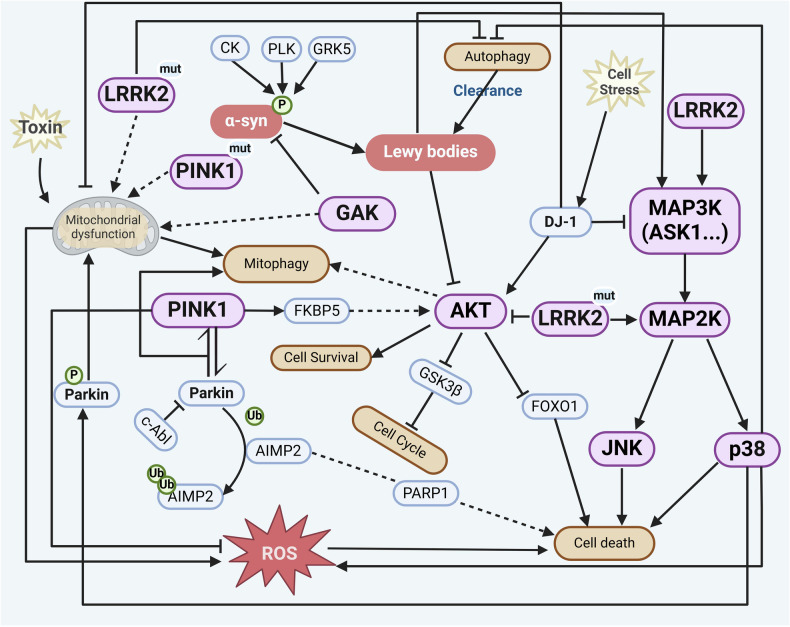
Schematic description of kinase signaling pathways in Parkinson’s disease. PINK1 and LRRK2 are involved in mitochondrial and lysosomal function, and their mutations lead to mitochondrial dysfunction and autophagy defects. These mutations induce typical α-syn aggregation in the form of Lewy bodies and neurites, as well as neuronal loss. The accumulation of PINK1 in the mitochondrial membrane and subsequent recruitment of Parkin can trigger the initiation of mitophagy. *LRRK2* mutations failed to phosphorylate AKT and promote cell survival via inhibition of FOXO1, whereas phosphorylated JNK and p38 MAPK, promoting cell death. Activated ASK1 can also phosphorylate MAPKK and subsequent MAPK to form a cascade amplification of signaling, promoting cell death by regulating the activation of JNK and p38. Phosphorylation of α-syn occurs at several sites involving kinases such as CK, PLK, c-Abl, and GRK, contributing to its aggregation and the formation of Lewy bodies, which alters the activity of numerous kinases and triggers neuroinflammation and increased ROS. This figure was created with BioRender.com

Kinases regulate α-syn during disease progression. While other causal genes encode kinases, such as *LRRK2* and *PINK1*, and their genetic mutations directly increase susceptibility to PD. In addition, other kinase signaling pathways including MAPK and AKT are also affected in PD. Activation of JNK or p38 MAPK in PD promotes the apoptosis of neurons,^327^ while activation of ERK1/2 MAPK and PI3K/AKT pathways supports cell survival.^328^ Here, we summarize the major PD risk genes highly associated with kinases and elucidate their multifaceted contributions to PD-related disturbance of kinase signaling pathways (Figs. 2 and 8).

##### LRRK2

The *LRRK2* gene, encoding the LRRK2, is one of the most commonly mutated genes in familial PD, and its mutations are also found in sporadic PD.^37^ LRRK2 kinase activity in dopaminergic neurons and microglia in the SNpc is increased in patients with idiopathic PD (iPD), suggesting the involvement of LRRK2 in iPD.^329^ Patients with *LRRK2* mutations are very similar to iPD in clinical features and treatment responses.^330,331^ G2019S, the most common *LRRK2* mutation in PD, accounts for 4% of familial PD cases and 1% of sporadic PD cases worldwide.^37^ The mutation induces typical α-syn aggregation in Lewy bodies and neurites, as well as neuronal loss in specific brain regions.

LRRK2 belongs to the TKL group and is a member of the leucine-rich repeat kinase family, with a Ser/Thr kinase domain, a GTPase functional enzyme domain, and protein-protein interaction domains such as armadillo, ankyrin, and WD40. LRRK2 is mainly present in the cytoplasm, but also in the intracellular membrane and microtubules under certain conditions.^332^ It primarily serves as a kinase for Rab GTPases, which are the main regulators of membrane transport, autophagy, and lysosomal degradation.^333^ Impairments of endolysosomal function and autophagy have been observed in PD involving *LRRK2* mutation.^334–336^ As LRRK2 regulates the balance between membrane repair and organelle replacement to maintain endolysosomal homeostasis, pathogenic mutations lead to membrane damage followed by disruption of endolysosomal homeostasis.^337^ In addition, wild-type LRRK2 can be degraded by chaperon-mediated autophagy in lysosomes, and mutations such as G2019S block such degradation pathway.^335^

The mechanisms relating to *LRRK2* mutations in PD pathogenesis are generally associated with the kinase activity of LRRK2, inhibition of which has displayed therapeutic effects in models of PD in vitro and in vivo.^338–340^ For instance, a novel LRRK2 kinase inhibitor, FL090, ameliorates lysosomal dysfunction and loss of dopaminergic neurons in the SNpc by upregulating microtubule-associated protein 1B in both genetic and pharmacological PD animal models.^341^ In addition, reducing LRRK2 expression with anti-sense oligonucleotide has recently shown promising results in ameliorating α-syn inclusion formation in preclinical trials.^342^ Therefore, a variety of LRRK2 kinase inhibitors, including WXWH0226 (https://synapse.patsnap.com/), ARV-102 (https://www.arvinas.com/), DNL201,^343^ and DNL151,^344^ are now being tested in clinical trials for PD.^345^

##### PINK1 and Parkin

Quality control such as the structure and function of mitochondria is achieved by homeostasis of degradation-related enzymes, mitophagy, mitochondria-derived vesicles, and other manners.^346^ Mitochondrial dysfunction is considered a key event in both familial and sporadic PD,^347^ and has been widely observed in autopsy of patients with PD.^348,349^ Reduced mitophagy is one of the forms of mitochondrial defects in PD patients and serves as a possible mechanism of pathogenesis, largely due to the loss-of-function of the key mitophagy regulators PINK1 and Parkin.^350^ The cytoplasmic protein Parkin, acting with the ubiquitin kinase PINK1 (a Ser/Thr protein kinase), mediates the mitochondria-related autophagy process, namely mitophagy.^351,352^ When mitochondrial damage is perceived, PINK1/Parkin is the first to activate the mitochondrial quality control pathways.^353^ In brief, PINK1 phosphorylates ubiquitin on Ser65, and stabilizes the active conformation of Parkin, allowing the charged E2 ligases to bind, therefore the E3 ubiquitin ligase activity of Parkin is enhanced.^354^ The accumulation of PINK1 on the mitochondrial membrane and subsequent recruitment of Parkin triggers the initiation of mitophagy.^355^

*PINK1* and *PRKN* mutations are the main causes of autosomal recessive PD, and dysfunctions of these proteins also contribute to early-onset PD.^356^ As for clinical manifestations, although the onset is relatively early, it shows slow progression, and cognition is rarely affected in patients with *PINK1* or *PRKN* mutations.^357,358^ The G309D and L347P mutations of *PINK1* result in a significantly reduced PINK1 activity,^359,360^ leading to the loss of its anti-apoptotic function by suppressing the release of cytochrome c from mitochondria.^361^ The accumulation of pathogenic substrates, such as aminoacyl-tRNA synthetase complex interacting multifunctional protein-2 (AIMP2), occurs as a consequence of *PRKN* mutations and Parkin inactivation.^362^ AIMP2 present in Lewy bodies causes DNA damage and cell death by activating poly (ADP-ribose) polymerase-1.^363^ The non-RTK c-Abl phosphorylates Parkin at Tyr143, resulting in decreased Parkin activity. 1-methyl-4-phenyl-1,2,3,6-tetrahydropyridine (MPTP)-induced c-Abl activation leads to the Parkin inactivation, which in turn causes the accumulation of AIMP2 and neuronal death.^364^ Furthermore, c-Abl was activated and Parkin was phosphorylated at Tyr143 in postmortem human brain tissue from PD patients.^364^ Therefore, c-Abl may serve as a pathogenic kinase in PD by inhibiting the Parkin activity.

##### GAK

Genome-wide association analysis of patients with familial PD revealed an association between single nucleotide polymorphisms at the *GAK* locus and PD susceptibility,^365^ which was supported by followed-up meta-analyses.^366–369^ Cyclin G-associated kinase (GAK) encoded by the *GAK* gene is a Ser/Thr kinase comprised of a kinase domain in the N-terminus and a clathrin binding domain.^370^ Neuron-specific knockout of *GAK* leads to cell loss in neonatal mice due to deficient proliferation of neural progenitors,^371^ and GAK inhibition is associated with neurodevelopmental disorders.^372^

GAK activity affects *PRKN*-independent mitophagy by altering the mitochondrial network and lysosome morphology, shedding light on the regulation of mitophagy.^373^ Downregulation of auxilin, the homolog of GAK in *Drosophila*, leads to impaired climbing ability, shortened lifespan, and dopaminergic neuron loss.^374^ In vitro experiments demonstrated that knockdown of GAK in cells overexpressing α-syn increased α-syn level and resulted in cytotoxicity,^375^ suggesting that GAK may play a protective role in PD.

##### AKT

Mutations in several PD-associated genes cause abnormalities in the AKT signaling pathway which maintains fundamental functions in dopaminergic neurons.^376^ In an in vitro model of PD, 1-Methyl-4-phenylpyridinium (MPP^+^) treatment has been found to inactivate AKT.^377^ PINK1 regulates the activation of insulin-dependent AKT signaling pathway,^378^ possibly by phosphorylating FK506 binding protein 5, and rescues the damage of mitochondrial complex I induced by MPP^+^.^379,380^ Consistently, the MPTP-induced inactivation of AKT was recovered by overexpression of Parkin.^381^ LRRK2 phosphorylates AKT and promotes cell survival via inhibition of forkhead box protein O1. G2019S and R1441C mutants of *LRRK2* reduce the phosphorylation of AKT, and subsequent blockade of pro-cell survival by AKT inhibition may contribute to neuronal death in PD.^382^ DJ-1, a homodimeric protein, acts as an oxidative stress sensor in stressed conditions.^383^ Loss-of-function mutations of *DJ-1* are associated with autosomal recessive PD.^384,385^ A cytoprotective role of DJ-1 has been proposed involving the activation of cell survival-related ERK1/2 and PI3K/AKT pathways.^386,387^ Therefore, restoring the function of AKT and activating the AKT signaling pathway may show protective benefits in PD.

##### JNK/p38

MAPK family members, belonging to the CMGC group, are dysregulated in PD, and among them, JNK and p38 MAPK are activated in various PD models.^388–392^ JNK2 and JNK3, but not JNK1, induce upregulation of cyclooxygenase-2 expression and activate c-Jun-induced death of dopaminergic neurons in the MPTP-induced PD model.^393^ The activation of c-Jun and up-regulated expression of cyclooxygenase-2 were also observed in dopaminergic neurons of PD patients.^394^ Toxins such as Rotenone and MPTP can directly or indirectly activate the p38 MAPK pathway, which in turn results in ROS accumulation.^395^ In α-syn-A53T transgenic mice, increased p38 MAPK activity leads to direct phosphorylation of Parkin and subsequent mitochondrial dysfunction.^391^ Additionally, α-syn activates toll-like receptor 4-dependent p38 MAPK and triggers autophagy impairment in microglia and induction of neuroinflammation.^392^

Interestingly, JNK and p38 MAPK can be indirectly phosphorylated by LRRK2 via MAP2K, possibly because the LRRK2 kinase domain has a high homology with the MAP3K family members.^396,397^ Moreover, PD-associated *LRRK2*-G2019S mutation has been found to exhibit augmented phosphotransferase activity for MAP2K.^396^ Several drugs, such as SKF-86002,^398^ SB203580,^399^ and SB202190,^400^ inhibiting JNK/p38 MAPK and their upstream pathways have been tested in several models of PD. However, one such inhibitor CEP-1347 failed to reach the clinical endpoint in a clinical trial.^401^ This indicates that treatment with broad effects needs to be used with caution when applied to human patients.

##### ASK1

ASK1, a member of the MAP3K family, phosphorylates and activates MAP2K and subsequent MAPK to amplify the downstream signaling cascades. ASK1 itself takes part in physiological processes as well as cell death, by regulating the activation of JNK and p38.^402^ In the MPTP model, ASK1-MAPK was activated possibly due to TNF-dependent thioredoxin-1 oxidation,^403^ and knockout of *ASK1* rescued MPTP-induced motor deficits, dopaminergic neuron loss, and neuroinflammation.^404^ ASK1 inhibitor, JNK3-N-Tat alleviated mitochondrial damages and inhibited the apoptosis of dopaminergic neurons in MPP^+^-treated primary cortical cells as well as MPTP mouse model.^405,406^ In addition, Apelin-36, a neuromodulatory peptide, protects neurons from apoptosis in the MPTP mouse model by inhibiting the ASK1/JNK/caspase-3 pathway.^407^

ASK1 activation was also found in α-syn-overexpressing cells and transgenic animal models, suggestive of the involvement of ASK1 in the cascade effects of α-syn.^408^ In support, ASK1 deletion rescues the behavioral deficits induced by intrastriatal injections of α-syn pre-formed fibrils and accumulation of phosphorylated α-syn in the striatum and cortex.^409^ ASK1 also activates cell death-related pathways after phosphorylated by LRRK2 at Thr832,^410^ and abolished activation of ASK1 mediated by DJ-1 shows protective benefits in oxidative stress-induced cell death.^411^ However, inhibitors of ASK1 (including Selonsertib,^412^ ASK1-IN-2,^413^, and GS-444217^414^) have not been yet tested in clinical PD.

### HD

HD is a monogenic autosomal dominant genetic disease caused by mutations in the *huntingtin (HTT)* gene, the abnormal copy of which will have a 50% of probability being passed to the offsprings.^415^ At the molecular level, tandem repeats of triplet CAG are abnormally expanded in the encoding region of *HTT* gene, resulting in an insertion of polyglutamine (polyQ) polypeptide in the N-terminal domain of the mutant HTT (mHTT) protein which drives the death of the brain cells.^416–418^ HD symptoms usually appear between the ages of 30 and 50, and are influenced by familial inheritance. For example, the age of onset may be earlier in families with associated chromosomal abnormalities and about 8% of cases show onset before the age of 20.^419,420^ Early symptoms manifest as mild emotional or intellectual impairment, with only an uncoordinated and unsteady gait. As the disease progresses, incoordination of body movements becomes more pronounced, and progressively and eventually worsens until movement becomes difficult, speech becomes impossible, and decline of mental abilities develops into dementia.^421^ The incidence of HD is similar between sexes, and is about 12 per 100,000 people in the European population, but the incidence in Africa and Asia is approximately one-tenth of that in European.^422^

In HD, mHTT protein is defective in its normal physiological functions, instead, misfolded mHTT fragments form a variety of intracellular aggregation structures with toxicity to adversely impair cell functions.^423^ HTT protein undergoes various types of PTM, including phosphorylation, acetylation, palmitoylation, and ubiquitination. These modifications modulate the toxicity of mHTT, leading to potential clinical implications. Growing evidence from cellular and animal models shows that phosphorylation or pseudophosphorylation of Ser13, Ser16, Ser434, or Ser536, in HTT reduces the toxicity of polyQ-mHTT, and levels of phosphorylation are weakened by polyQ expansion,^424–426^ suggesting that phosphorylation state of HTT tightly links to toxicity in HD pathogenesis. Phosphorylations of HTT at Ser13 and Ser16 have been most studied, and are found to regulate the structure, aggregation, and subcellular localization of HTT.^425^

#### Major kinases in HD

**Fig. 9:**
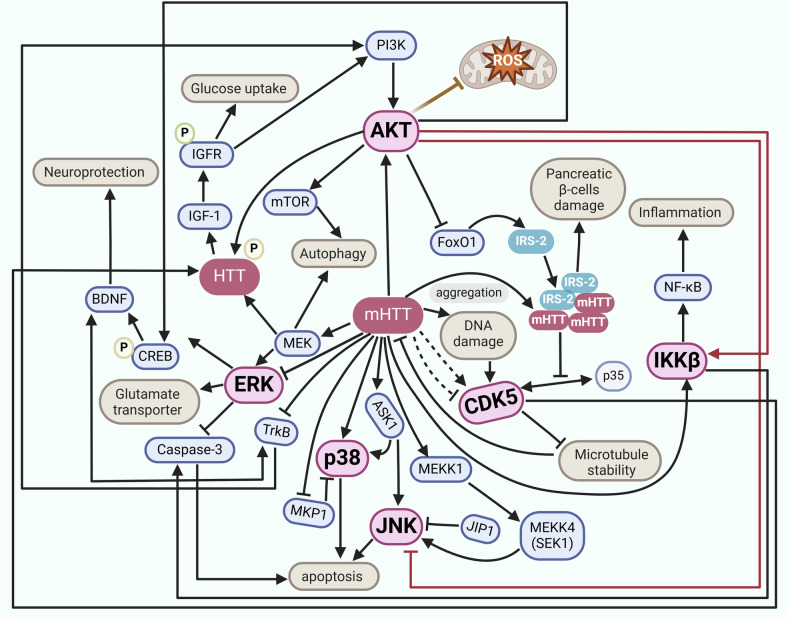
Schematic description of kinase signaling pathways in Huntington’s disease. mHTT promotes neuronal apoptosis and inflammatory responses by inhibiting ERK and activating the p38, JNK, and IKKβ signaling pathways. It establishes connections with HTT via the MEK/ERK and AKT signaling pathways, regulating neuronal autophagy, neuroprotection, and glucose uptake pathways. In addition to causing DNA damage by itself, mHTT’s aggregation also leads to pancreatic β-cells damage and activation of CDK5 through its interaction with IRS-2. The aberrant activation of CDK5 reduces microtubule stability, which, in turn, contributes to the exacerbation of mHTT pathology. In addition, the activation of the IKKβ signaling pathway by AKT promotes neuroinflammatory responses, while the inhibition of the JNK signaling cascade by AKT hinders cell apoptosis. The regulatory network formed by the mutual activation and inhibition of kinases plays distinct roles in different stages of the HD pathological process. This figure was created with BioRender.com

Kinases are involved in the pathophysiology of HD through multiple regulatory processes including integration of glutamate transmission and BDNF signaling, neuroimmune regulation, resistance to mHTT toxicity, neuronal apoptosis, cellular energy metabolism, and autophagy pathways. Phosphorylation of HTT at Ser1/Ser16 by IKKβ increases the clearance of mHTT by proteasomes and lysosomes thereby reducing mHTT-mediated cytotoxicity.^427^ Similarly, phosphorylation of HTT at Ser434 by CDK5 ameliorates HTT aggregates-stimulated cell toxicity and cell death.^88^ Interestingly, autophagic clearance of mHTT and suppressed accumulation of mHTT are found to be facilitated by TBK1-medicated HTT phosphorylation.^428^ In addition, kinases such as AKT, ERK, and JNK are also phosphor-activated to mediate the toxicity of mHTT.^429–431^ Here we summarize the major kinases and their regulatory mechanisms in HD pathogenesis (Figs. 2 and 9).

##### MEK/ERK

Studies have concluded that MEK (a member of the MAP2K family in the STE group) /ERK (a member of the MAPK family in the CMGC group) acts as a double-edged sword in HD. On the one hand, MEK/ERK signaling is considered to integrate glutamate transmission and BDNF signaling in HD.^415^ In R6/1 and R6/2 mouse models expressing exon 1 of *HTT*, wide variations in ERK activation related to age, brain regions, and cell types are identified.^432,433^ Mice expressing full-length HTT tend to exhibit suppressed ERK activity and downstream BDNF expression.^434^ ERK deficiency in the mHTT model triggers striatal degeneration and increases glutamate susceptibility.^435,436^ Therefore, reduced ERK activation leads to lessened trophic support by BDNF, and upregulated expression of glutamate transporters (reviewed elsewhere^415^), leading to subsequent neuronal excitotoxicity and apoptosis.

On the other hand, MEK/ERK activation is believed to be neuroprotective in HD.^430,432^ The reduction of nutrients obtained by striatal neurons will in turn activate ERK to increase compensatory responses.^430^ Increased MEK activity promotes the activation of ERK/CREB, reduces the caspase-3 activity, increases the phosphorylation of HTT and autophagic clearance of mHTT, and ultimately supports neuronal survival.^415,436,437^

##### IKK

IKK is a member of the other group of typical protein kinases and is a complex composed of two catalytic subunits IKKα and IKKβ, and a regulatory subunit IKKγ.^438^ The human IKK family has four members, the IKKα, IKKβ, TBK1, and IKKε. IKK directly interacts with mHTT,^439^ and chronic elevation of IKK caused by DNA damage and NF-κB pathway activation is associated with neurodegeneration in HD.^440–442^ Clinical data indicate that HD patients exhibit high levels of inflammatory factors resulting from the chronic increase of IKK/NF-κB activity in the CNS and serum specimen/samples before symptom onset, with the immune cells maintained in a sensitive state.^438,440,443^ Similar effects of IKK/NF-κB signaling involving the immune environment were observed in the HD cell model and R6/2 mouse brains,^439,444^ and increased IKK in Hdh^Q150^ mice was observed in the striatum.^445^

Specifically, IKKβ phosphorylates HTT at Ser13/Ser16, which activates the scavenging of HTT, and knockout of *IKKβ* further deteriorates deficits in motor behavior of R6/1 HD mice, accompanied by striatal neurodegeneration and microglia activation.^446^ Hence, IKKβ may protect the striatum from neuronal degeneration and slow HD progression through multiple mechanisms.^427,446^ However, since IKK is regulated by various factors such as AKT, TNF-α, and interleukin-1β, and both the activation and inhibition of IKKβ have been shown to exert neuroprotective effects on HTT aggregation,^447–449^ the confirmative role of IKKβ in HD needs to be further elucidated.

##### CDK5

CDK5 belongs to the CDK family, the activity of which is abnormally elevated in AD pathology, whereas in HD, reduced levels of CDK5 and p35 have been detected in human HD patients and brains of HD mouse model.^426,450^ In the mouse model, although CDK5 reduces the HTT aggregation by phosphorylating HTT at Ser434 and reduces its cleavage by caspases,^88^ phosphorylation of HTT at Ser434 by CDK5 was repressed.^426^ In addition, DNA damage-triggered CDK5 activation causes phosphorylation of HTT at Ser1181 and Ser1201, thereby protecting striatal neurons from mHTT-induced toxicity.^88^ Mitigated CDK5 activity stabilizes microtubules and facilitates the formation of mHTT inclusion bodies in HD.^451^ Conversely, CDK5 activation has been shown to dissociate microtubules in primary cortical neurons, thereby weakening mHTT aggregation.^451–453^ However, enhanced CDK5 activation in StHdh^Q111^ cells promotes cell death, possibly through dysregulation of glutamatergic and dopaminergic signaling.^454^ Therefore, single-targeting CDK5 to increase its activity may produce certain toxic side effects during treatment for HD.

##### JNK/p38

As a member of the MAPK family, JNK activates the transcription factor c-Jun, one of the activator protein-1 transcription complexes, to regulate the expression of pro-apoptotic genes.^455,456^ JNK also phosphorylates and stabilizes p53 thereby enhancing the pro-apoptotic activity of p53.^457^ Knockout of CNS-specific *JNK3* exerts resistance to excitotoxicity and increases PI3K/AKT activity, implying that JNK3 is antagonistic to AKT signaling.^458,459^ So far, upregulated JNK signaling can be observed in most animal models of HD, except the *StHdh*^Q111^ knockin model.^415^ Activation of MAP2K4 and MAP3K1, upstream activators of JNK, accelerates the formation of mHTT inclusion bodies and augments the resultant toxicity in HD transgenic models.^460,461^ Negative regulatory mutations of MAP3K1 and JNK-interacting protein 1 reduced dopamine and adenosine 3’5’-monophosphate-regulated phosphoprotein 32 kDa, but did not alter c-Jun expression and mHTT inclusion formation in a rat lentiviral model of HD.^462^ Furthermore, ASK1 activated by mHTT acts as an upstream kinase of JNK and p38 to indirectly promote apoptosis.^463,464^ Therefore, inhibition of JNK activation protects neurons from mHTT-induced toxicity and apoptosis.

In HD patients and mouse models, increased p38 activity is found to correlate with neuronal death.^465,466^ Phosphatase MAPK phosphatase 1 inactivates p38 by dephosphorylating p38 in the Thr-Gly-Tyr motif, and the decreased MAPK phosphatase 1 activity therefore accounts for enhanced p38 activity.^467^ Pharmacological stimulation of MAPK phosphatase 1 reduced p38 activity and protected cultured cells and mHTT-injected mice from neurotoxic outcomes resulting from mHTT expression.^101^ In HD mice, increased activation of p38 is partially responsible for striatal degeneration, and p38 inhibitor SB-239063 successfully protects neurons from degeneration,^468,469^ which may involve blockade of neuronal apoptotic signals and glial cell-mediated neuroinflammation.

##### AKT

Activation of AKT signaling in all HD cells, mouse models, and patients’ brains has been suggested to be neuroprotective.^415,470,471^ Phosphorylation of mHTT by AKT is essential for IGF-mediated neuroprotection,^429^ while Mn^2+^-induced reduction of p-IGFR/IR-dependent AKT phosphorylation leads to reduced glucose uptake in HD cells.^472^ In addition, insulin and IGF-1 signaling pathway improves mitochondrial function and mitigates reactive oxygen species production in a PI3K/AKT-dependent manner in HD knockin striatal cells,^473^ while enhancing autophagy by modulating PI3K/AKT/mTOR signaling improves HD-like lesions in rats.^474^ In the study of the relationship between HD and the pathogenesis of diabetes, it was shown that in the mouse pancreatic insulinoma cell line NIT-1 (160Q cells) expressing the N-terminal mHTT containing 160 polyglutamine, insulin receptor substrate 2 was recruited to the mHTT aggregates in HD β cells, leading to the inhibition of the PI3K/AKT/forkhead box protein O1 pathway and causing damage to pancreatic β cells.^475^ In addition, 11-week-old R6/2 mice showed smaller pancreatic islets, loss of insulin, glucagon, and somatostatin expression, and reduced activation and expression of PKA, AKT, ERK1/2, and STAT3 in the pancreas.^476^ These studies suggest that diabetes or abnormal glucose and lipid metabolism may be involved in the development of HD. mHTT regulates both pro-survival and pro-apoptotic signals via CDK5/AKT modulation.^415^ In brief, mHTT activates CDK5/AKT signaling to promote HTT phosphorylation for cell survival; on the other hand, the same process may inhibit JNK/caspase-3 signaling, accelerating cell apoptosis. The indication of this line of events depends on the diversities of research models and experimental conditions.

### ALS

ALS is a rare but fatal neurodegenerative disease characterized by progressive degeneration and loss of motor neurons. Familial ALS (fALS) accounts for 10–15% of all cases,^477^ and more than 40 ALS-related genes have been identified, including *C9orf72*, *TARDBP*, *SOD1*, and *FUS* genes.^478^ Variations in these four genes account for ~60% of fALS and 10% of sporadic ALS.^479^ In addition to genetic factors, environmental elements such as exposure to chemicals, metal contaminations, or radiation, can increase the risk of ALS through mechanisms such as augmented neuronal oxidative stress and mitochondrial damage.^480^ These factors also, by affecting protein phosphorylation, drive disease progression and selective degeneration of motor neurons.

Mutations in the TDP-43-encoding gene *TARDBP* account for approximately 4% of fALS patients.^481^ Interestingly, the TDP-43 pathology appears in the vast majority of ALS patients.^482^ Normally, TDP-43 is predominantly localized in the nucleus. However, pathological aggregates of TDP-43 in the cytoplasm are present in ALS pathology.^483^ Various PTM of TDP-43 such as ubiquitination,^484^ acetylation,^485^ and phosphorylation,^486^ phosphorylation on multiple sites (Ser409, Ser410, Ser379, Ser403, and Ser404) have been observed in experimental and clinical ALS specimens, and other potential phosphorylation sites in TDP-43 are continuously discovered.^487^ With the aggravation of TDP-43 pathology, the cognitive decline appears more and more rapid.^488^ Possible causes of TDP-43 toxicity involve loss of normal physiological activity, gain-of-function mutation induced by pathological aggregation, or combined effects.^489^

Mutations in *FUS* cause ALS in an autosomal dominant manner, and most *FUS* mutations lead to mislocalization of FUS (fused in sarcomas) from the nucleus to the cytoplasm as inclusions.^490^ FUS is a DNA/RNA binding protein mainly expressed and localized in the nucleus, and participates in physiological processes such as DNA repair and RNA metabolism.^491^ Transgenic mice overexpressing wild-type human *FUS*, as well as expressing ALS-associated *FUS* mutants or *FUS* lacking a nuclear localization signal greatly triggered the formation of cytoplasmic FUS inclusions, and exhibited ALS-like motor impairments.^492–494^ In both mouse and human brain tissue, FUS has been observed to bind to over 5500 pre-mRNAs.^495^ Knockdown of *FUS* results in decreased expression of mRNAs containing long introns, many of which encode proteins involved in synaptic function, such as neurexin 3 and neuroligin 1.^495^ Hence, the contribution of FUS in ALS may be primarily due to the loss-of-function of normal FUS in the nucleus and the gain-of-function of toxic FUS secondary to the mislocalized FUS aggregation in the cytoplasm.

To date, more than 200 *SOD1* variations have been disclosed in ALS (https://alsod.iop.kcl.ac.uk/), occurring in 14.8% of European fALS patients and 30% of Asian fALS patients.^496^ Cu/Zn superoxide dismutase 1 (SOD1), encoded by *SOD1*, is an antioxidant enzyme that regulates mitochondrial and cytosolic superoxide levels. Mutant SOD1 forms unstable depositions in the cytoplasm, and then greater aggregates and inclusions,^497^ which impair axonal transport.^480^ The spinal motor neurons in SOD1^G93A^ transgenic mice showed mitochondrial membrane potential loss, respiratory chain activity disruption, calcium homeostasis disruption, and mitochondrial vacuolation.^498^ However, for a long time, the loss-of-function hypothesis was not recognized due to the lack of correlations between enzyme activity of SOD1 and aggressiveness of clinical phenotypes in ALS patients,^499^ as well as the absence of obvious phenotypes in *SOD1* null mice in early studies.^500^ Nonetheless, a comprehensive review^501^ on SOD1-ALS patients revealed that almost all mutations lead to a substantial reduction in SOD1 enzyme activity, and significant deleterious effects in the nervous system were evident in *SOD1* knockout mice, supporting the contribution of loss-of-function mutation of *SOD1* to ALS pathogenesis.

Defects in mitochondrial morphology, functions, and dynamics were consistently observed in ALS patients and mouse models. C9orf72 is a mitochondrial inner membrane-associated protein stabilizing mitochondrial complex I assembly,^502^ and haploinsufficiency and loss-of-function mutation of *C9orf72* lead to decreased activity of neuronal mitochondrial complex I.^502,503^ Given the discovery of C9orf72 hexanucleotide repeat expansions as a critical cause of ALS,^504,505^ studies have ever since revealed the multifaceted effects of C9orf72. In addition to the detrimental effects on mitochondrial functions mentioned above, mutated C9orf72 also impairs a variety of intracellular processes such as RNA metabolism and autophagy. C9orf72 haploinsufficiency also negatively affects vesicle trafficking and inhibits the initiation of autophagy.^506^ Inhibition of autophagy impedes the clearance of misfolded and aggregated proteins, such as TDP-43 and FUS aggregates in ALS, thereby exacerbating cellular damage.

To date, Rilutek and Radicava have been approved by the FDA for ALS treatment to slow the progression of symptoms and improve the quality of life, although they are unable to reverse the existing damages caused by ALS.^507^ Continuing endeavors into the pathophysiology of ALS have led to several potential mechanism-based therapies that are currently being tested in clinical trials.

#### Major kinases in ALS

**Fig. 10:**
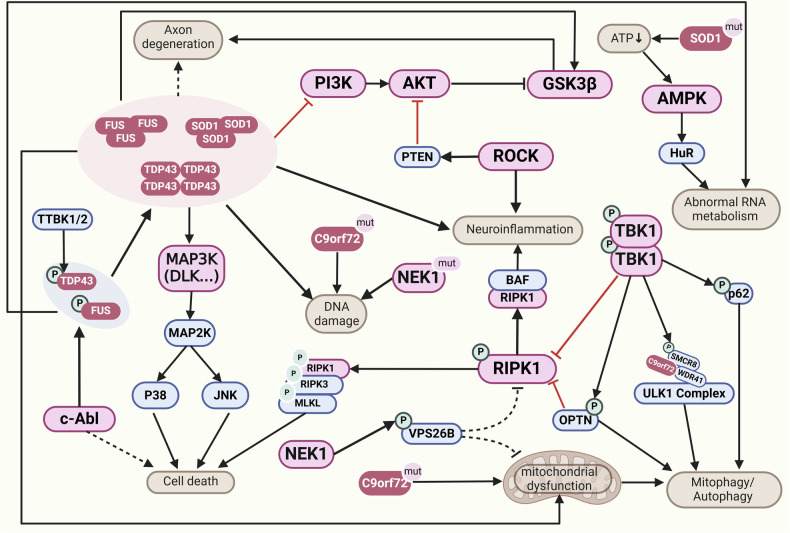
Schematic description of kinase signaling pathways in Amyotrophic lateral sclerosis. ALS genes encoding protein kinases, such as *TBK1* and *NEK1*, affect the proteostasis process, autophagy, and DNA damage. TBK1 phosphorylates OPTN and p62, leading to autophagy clearance and thus ensuring efficient degradation of ubiquitinated mitochondria. TBK1 inactivates RIPK1 and loss of TBK1 boosts RIPK1 activation and promotes cell death. NEK1 mutations disable DNA damage response and its deficiency reduced the phosphorylation of VPS26B, leading to disruption of endosomal transport and further dysfunction of mitochondria and lysosome. Several kinases are involved in the phosphorylation of TDP-43 and others. The decrease of AKT activity in ALS leads to the upregulated GSK3β activity, contributing to TDP-43-induced axonal degeneration. c-Abl kinase mediates the accumulation of toxic FUS by phosphorylating FUS. Proteins abnormally aggregated alter the kinase signaling networks, eventually leading to cell death of motor neurons. This figure was created with BioRender.com

Protein kinase-encoding genes such as *TBK1* and *NEK1* were recently discovered as ALS causal genes, as well as that several other kinases are widely involved in ALS pathogenesis (Figs. 2 and 10). In general, they take part in the phosphorylation of causative proteins in ALS such as TDP-43, leading to altered functions and abnormal aggregation of these proteins. Furthermore, dysregulation of the kinase signaling network may directly contribute to the death of motor neurons via complex interactions.

##### TBK1

Whole-exome sequencing data of 2869 ALS patients and 6405 controls have identified TBK1 as an ALS-associated gene,^508^ further supported by another independent study showing that the haploinsufficiency of TBK1 caused ALS.^509^ Thereafter, the pathogenic role of ALS-*TBK1* mutations has been validated,^510–512^ e.g., nonsense, frameshift, and missense mutant forms were found in sporadic and fALS patients.^513^

TBK1 is a IKK family protein, composed of a Ser/Thr kinase domain with two lobes, a ubiquitin-like domain, and two coiled-coil domains.^514^ Activation of TBK1 involves a multi-step mechanism whereby Lys30 and Lys401 are first poly-ubiquitinated, followed by phosphorylation of Ser172, resulting in a conformational change to allow substrates to bind.^176^ Regulated by adaptor proteins such as NAK-associated protein 1, TANK, and Sintbad, TBK1 participates in physiological activities such as induction of interferons and autophagy regulation.^515^ Activated TBK1 modulates autophagy by phosphorylating autophagic adaptor proteins and membrane components in autophagosome such as LC3 and C9orf72-binding partner SMCR8.^516,517^ In addition, other autophagy receptors OPTN, nuclear dot protein 52, Tax1 (human T cell leukemia virus type I) binding protein 1, and p62, are all TBK1 substrates, and phosphorylation of these receptors leads to autophagy clearance and degradation of ubiquitinated mitochondria, a process of which is of great importance considering that both TBK1 and OPTN are genetically linked to ALS.^518^

ALS-associated *TBK1-S172A* mutation blocks the efficient formation of autophagosomes and damages autophagosomal membranes, suggesting that impairment of mitophagy may be a key pathophysiological mechanism for ALS.^517,519^ In the early disease stage of SOD1^G93A^ mice, the heterozygous deletion of *TBK1* impairs autophagy in motor neurons and precedes disease onset.^520^ However, in the late disease stage, heterozygous *TBK1* deletion shows opposite effects of significantly attenuated microglial neuroinflammation and prolonged survival.^520^ Similarly, *TBK1* G217R and R228H mutations show decreased kinase activity, thereby accelerating the onset of disease in SOD1^G93A^ mice, whilst extending their lifespan.^177^ Given these observations, TBK1 may exert a multifaceted role in SOD1-related ALS.

##### NEK1

Another ALS-associated gene *NEK1* has also been screened out from a whole-exome analysis of familial and sporadic ALS patients, and *NEK1* variations usually result in a loss-of-function outcome for the *NEK1*-encoding kinase NEK1.^521–524^ Comparing hiPSC-derived motor neurons carrying a *NEK1* mutation and neurons carrying a *C9orf72* mutation with wild-type neurons, neurons with *NEK1* mutation showed the most severe DNA damage, implying that *NEK1* mutations disrupt DNA damage response in ALS pathology.^525^ In addition, NEK1 deficiency reduces the phosphorylation of VPS26B and causes disruption of endosomal transport and consequent dysfunction of mitochondria and lysosome.^526^

##### DLK

As a member of the mixed-lineage kinase, DLK acts upstream of MAPK signaling and activates JNK and p38 via MAPK-kinase 4/7.^527^ DLK in the CNS facilitates the establishment of neural circuits during development^528^ and acts as an axonal damage sensor in diseased states.^527,529^ DLK/JNK activity is profoundly elevated, and pharmacological inhibition or genetic knockout of *DLK* reduces the neuronal loss and axonal degeneration in ALS mouse models.^530^ Moreover, overexpression of activating transcription factor 3 post-DLK deletion significantly protects motor neurons from death and axonal degeneration in ALS mice, highlighting the feasibility of combination therapy in ALS.^531^

##### RIPK1

RIPK1 mainly regulates cell death and activates inflammatory pathways in ALS patients.^71^ TBK1 deficiency in mice causes strong activation of RIPK1 and embryonic death, which can be rescued by RIPK1 inactivation. Mechanistically, TBK1 inactivates RIPK1 by phosphorylating RIPK1 at Thr189, and loss of TBK1 boosts RIPK1 activation and aggravates late-onset ALS/frontotemporal dementia-like pathology,^532,533^ providing insights on intermolecular regulation between TBK1 and RIPK1 in ALS. In addition, defects in glucose uptake caused by NEK1 depletion stimulate RIPK1 activation, while inhibition of RIPK1 restores BBB damage, neuroinflammation, and accumulation of misfolded proteins, suggesting ALS may be treated by blocking RIPK1 activation.^534^

Mutations in another gene *OPTN* are associated with both familial and sporadic ALS.^71^ Expression of OPTN encoded by *OPTN* attenuates RIPK1-dependent cell death by modulating the turnover of RIPK1, and loss of *OPTN* leads to progressive axonal degeneration through RIPK1-dependent necrosis.^71,535^ RIPK1/BRM-associated factor complex was recently reported to promote the transcription of pro-inflammatory genes in ALS patients as a key regulator of chromatin remodeling.^536^ These results highlight the importance of RIPK1 in ALS, and consequently, RIPK1 inhibitors have entered clinical trials as a new class of drugs for the treatment of ALS.^537^

##### AMPK

AMPK coordinates cellular energy homeostasis by promoting catabolism and reducing ATP consumption. In ALS, AMPK activity is dysregulated, and abnormal activation of AMPK has been observed in motor neurons of ALS patients.^538^ As ALS progresses, patients may lose weight and body fat, or even exhibit malnutrition, stongly suggesting a metabolic dysfunction and an energy imbalance.^539^ Consistently, in the SOD1^G93A^ mouse model, AMPK activity is increased in motor neurons, likely attributed to a severe reduction in glucose uptake and a significant decline in ATP production.^540,541^ Activation of AMPK disrupts the distribution of human antigen R, a well-characterized mRNA stabilizer mainly located in the nucleus, through the phosphorylation of importin,^538^ which leads to an imbalance in RNA metabolism, presenting an alternative mechanism for the pathogenesis of ALS.

Nevertheless, controversial results have been reported on AMPK activation for ALS treatment. Several studies have indicated that the inhibition of AMPK activity may offer protection against ALS. Reducing aak-2, an ortholog of AMPKα2 catalytic subunit, improved the motor behavior in *SOD1*-G85R mutant nematodes.^542^ Disease progression of TDP-43 mutant mice can also be delayed by down-regulating AMPKα1 or inhibition of AMPK activity using cAMP analogs.^543^ In another study, the activation of AMPK by latrepirdine delayed the onset of motor symptoms and expanded the lifespan of SOD1^G93A^ mice.^544^ The effect of AMPK activation or inhibition on ALS may depend on the specific model used and the time window for pharmacological intervention, and future studies are still desired to determine whether the modulation of AMPK activity can represent a viable therapeutic avenue for ALS patients.

##### PI3K-AKT-GSK3β pathway

As one of the major anti-apoptotic pathways, the PI3K/AKT signaling is suppressed in the spinal cord of an ALS mouse model^545^; consequently, downstream proteins and their phosphorylation are broadly affected including GSK3β. AKT phosphorylates GSK3β at Ser9 thereby down-regulates GSK3β activity.^546^ Consequently, it is expected that the reduced AKT activity in ALS enhances GSK3β activity. Consistently, enhanced GSK3β activity was present in both animal models and patients with ALS,^547,548^ and GSK3β inactivation showed neuroprotective effects in ALS pathogenesis, partly through amelioration of phenotypic defects caused by expression of TDP-43, SOD1, or FUS mutants.^549^ GSK3β is also involved in TDP-43-induced axonal degeneration, since the loss of GSK3β/Shaggy inhibits TDP-43^Q331K^-mediated degeneration of axon and neuromuscular junction in *Drosophila*.^550^ A small molecule chemical, kenpaullone, was screened out and found to significantly prolong the survival of embryonic stem cell-derived motor neurons from SOD1 mice, partly due to its inhibitory effects on GSK3 activity.^551^

##### ROCK

Both isoforms of ROCK (ROCK1 and ROCK2) are Ser/Thr kinases.^552^ ROCK is catalytically inactive in its natural state as the kinase domain is automatically blocked by the C-terminal region.^553^ Expression of ROCK in adult mouse brains demonstrates that glial cells express ROCK1 while neurons express ROCK2,^554^ and ROCK2 levels are significantly increased in the skeletal muscle of patients with ALS.^555^ Upregulated ROCK activity activates phosphatase and tensin homolog and decreases AKT activity in ALS mice model^556^; ROCK inhibitor Fasudil greatly prolonged the survival of ALS mice, accompanied by reduced phosphatase and tensin homolog phosphorylation and restored AKT activity, in addition to increase the number of protective microglia in the spinal cord and decreased secretion of pro-inflammatory cytokines and chemokines in the early stage of the disease (day 100).^557^ Considering this drug is clinically available, it may be a promising treatment for ALS trials.

##### c-Abl

c-Abl is a member of the non-RTKs family with DNA binding domain sequence, nuclear export sequence, and nuclear localization signal. c-Abl participates in neurite extension and dendritic spine stability directly through its F-actin-binding region or indirectly through phosphorylation of actin-binding proteins (such as the small GTPase RhoA/Rac1 and CDK5),^558,559^ and it has recently emerged as a potential target for neurodegenerative diseases.^560^ Increased phosphorylation of Src/c-Abl was found in post-mortem spinal cord tissues of ALS patients, and inhibition of Src/c-Abl by pharmacological or genetic manipulations promotes the survival of motor neurons in various ALS models.^561,562^ Phosphorylation of pathological ALS proteins such as TDP-43 and FUS also involves c-Abl,^563,564^ e.g., c-Abl-mediated phosphorylation of TDP-43 at Tyr43 leads to its cytoplasmic accumulation and triggers neuronal cell death.^563^ Similarly, c-Abl kinase stimulates the cytoplasmic mislocalization and accumulation of toxic FUS by phosphorylating FUS at Tyr526.^564^

##### Kinases that phosphorylate TDP-43

TDP-43 is recognized as a major target of ALS, and the several kinases responsible for TDP-43 phosphorylation have been identified. In TDP-43^cKO^ mice, IKKβ promoted the degradation of cytoplasmic TDP-43 by proteasomes in hippocampal neurons via phosphorylating Thr8 and Ser92 at the N-terminus of TDP-43.^565^ IKKβ also significantly reduced the expression level and toxicity of pathogenic TDP-43-encoding gene *TARDBP* mutations (A321V and K181E) in N2a.^565^ Negative regulation of the cAMP/PKA signaling pathway by the phosphodiesterase dunce and inhibitory subunit PKA-R2 promotes the aggregation and mislocalization of the *Drosophila* TDP-43 ortholog *TBPH* in the cell bodies of motor neurons, leading to motor defects and shortened lifespan.^566^ Overexpression of the PKA target CrebA rescued TBPH mislocalization in the TBPH-overexpressing ALS *Drosophila* model.^566^ Therefore, activation of cAMP/PKA may be a strategy to improve the molecular and functional effects of pathological TDP-43, although this study was only in *Drosophila*.

CK1 phosphorylates multiple Ser residues in the glycine-rich region of the C-terminus of TDP-43 (including Ser379, Ser403/404, and Ser409/410), suggesting that CK1 may be involved in the pathological phosphorylation of TDP-43 in vivo.^486,567^ CDC7 strongly phosphorylates Ser409/410 of TDP-43, promoting neurotoxicity in TDP-43 transgenic animals.^568^ TTBK1/2 also strongly phosphorylates TDP-43 at Ser409/410, leading to synergistic exacerbation of behavioral abnormalities and increased pathological protein phosphorylation in TDP-43/TTBK1 transgenic *Caenorhabditis elegans*.^569^ In addition, elevated TTBK1/2 protein levels were found in the postmortem frontal cortex of patients with frontotemporal lobar degeneration, and TTBK1/2 colocalized with TDP-43 inclusions in ALS spinal cord.^570^ These studies suggest that targeting kinases may be an effective strategy for treating TDP-43 proteinopathy.

### Kinases in other neurodegenerative diseases

Kinases also are considered crucial in other neurodegenerative diseases, such as spinocerebellar ataxias (SCAs) and chronic traumatic encephalopathy (CTE).

Autosomal dominant SCA is a chronic progressive neurological disease, in which SCA type 14 is affected by point mutations in *PRKCG* (encoding PKCγ),^571^ a Ser/Thr protein kinase. Specifically, mutations in *PRKCG*, such as D115Y, enhance the basal activity of the kinase by compromising its autoinhibition, thereby promoting SCA type 14 disease progression.^572^ A study on SCA type 17 has shown that activation of MAPK/ERK occurs in the cerebellum of SCA type 17 mice, and that increased gliosis due to ERK activation may lead to neural apoptosis, suggesting that Purkinje cell loss may lag behind ERK activation in the SCA type 17 mouse model.^573^ In SCA type 11, TTBK2 inhibits disease progression by initiating ciliogenesis in vivo, and dominant truncating mutations in human *TTBK2* cause SCA type 11.^574^ The NMDA receptor NR2D subunit interacts with the SH3 domain of c-Abl, inhibiting the autophosphorylation activity of c-Abl, thereby reducing the loss of cerebellar neuronal function, suggesting that changes in c-Abl activity are associated with the development of cerebellar ataxia.^575^ Therefore, abnormal activation of kinases and mutations in specific sites of their encoding genes may lead to diverse types of SCAs.

CTE is a progressive neurodegenerative disease associated with repeated traumatic brain injury (TBI) that causes symptoms of cognitive impairment, behavioral changes, mood disturbances, and movement disorders.^576^ The pathology of CTE mainly includes the accumulation of hyperphosphorylated tau in neurons around blood vessels, and a unique molecular structural configuration of p-tau fibrils that is distinct from changes observed in aging, AD, or any other tauopathies.^577^ DAPK1 was found to induce cis-p-tau conformational changes and neurodegeneration by phosphorylating Pin1 (a unique prolyl isomerase known to inhibit the conformational state of p-tau) at Ser71 in the mouse brain of CTE model.^578^ In CTE brains, overactivation of GSK3β and CDK5 increased abnormal phosphorylation of tau, further leading to synaptic defects, a process that could be reversed by inhibition of GSK3β and CDK5.^579^ In the mouse model of TBI, the activity of ASK1-K716R was significantly reduced. Specifically increasing the activity of ASK1-K716R may maintain the integrity of the BBB, decrease the number of inflammatory microglia/macrophages, and white matter damage, and improve the nerve conduction of nerve fibers after TBI by suppressing the kinase activity of ASK1/JNK and p38.^580^ Activation of JAK2/STAT3 promotes the expression of interleukin-2 receptor γ, interleukin-4 receptor α in astrocytes, thereby increasing the inflammatory response in the cortex and hippocampus of adult male rats.^581^ Furthermore, axonemal dynein light intermediate polypeptide 1 was shown to promote neurodegeneration after TBI by preventing the clearance of phosphorylated tau via inhibition of autophagosome-lysosome fusion.^582^

## Clinical trials targeting kinases in neurodegenerative diseases

As summarized above, protein kinases are crucial for disease pathogenesis. In AD, the regulatory roles of kinases are predominantly manifested in tau phosphorylation, Aβ metabolism, and neuronal survival activities. In PD, kinases regulate α-syn phosphorylation, mitochondrial dynamics, autophagy pathways, and formation/plasticity of neuronal synapses. In HD, the occurrence is facilitated by PKC kinase which phosphorylates HTT and alters its structure and function, resulting in further aggregation and damage to the neurons. As one of the hotspots in ALS-related research, kinases participate in ALS pathogenesis by regulating the phosphorylation and aggregation/metabolism of pathological protein TDP-43 and other causative proteins in ALS. In other diseases such as SCAs and TBI, *PRKCG* gene mutations and abnormal activation of MAPK/ERK, c-Abl, and TTBK2 are involved in the pathogenesis of SCAs, while abnormal activation of DAPK1, GSK3β, CDK5, JAK2 and decreased activity of ASK1-K716R promote neuropathy after TBI. Collectively, these findings support the notion that drug development targeting protein kinases may provide new directions and prospects for neurodegenerative diseases. Several clinical trials with kinase-targeted drugs are completed or currently underway (Table 1 and Table 2).

**Table 1:** Drugs targeting protein kinases involved in neurodegenerative disease in clinical trials (can be found at https://clinicaltrials.gov/)

NCT Number	Compound	Target	Conditions	Phase	Status	Combination Therapy
NCT05194163	MW150	p38αMAPK^583–585^	Alzheimer’s disease	phase II	Not yet recruiting	None
NCT02423200	VX-745	p38αMAPK^586^	Alzheimer’s disease	phase II	Completed	None
NCT02423122	VX-745	p38αMAPK^586^	Alzheimer’s disease Mild cognitive impairment	phase II	Completed	None
NCT03402659	Neflamapimod	p38αMAPK^586^	Alzheimer’s disease	phase II	Completed	None
NCT00088387	Lithium	GSK3^598–600^	Alzheimer’s disease	phase II	Completed	Divalproex
NCT00948259	NP031112	GSK3β^592^	Alzheimer’s disease	phase I/II	Completed	None
NCT01055392	Lithium Carbonate	GSK3^598^	Cognitive Impairment Alzheimer’s disease	phase II	Unknown	None
NCT05564169	Masitinib (4.5)	Tyrosine kinase^610^	Mild to moderate Alzheimer’s disease	phase III	Recruiting	Cholinesterase inhibitors (donepezil, rivastigmine or galantamine) and/or memantine
NCT05143528	Nilotinib BE (84 mg or 112 mg)	Tyrosine kinase^610^	Early Alzheimer’s disease	phase III	Not yet recruiting	None
NCT01872598	Masitinib	Tyrosine kinase^610^	Mild to moderate Alzheimer’s disease	phase III	Completed	Cholinesterase inhibitor (donepezil, rivastigmine or galantamine) and/or memantine
NCT00606164	Bryostatin 1	Protein kinase C^616^	Alzheimer’s disease	phase II	Unknown status	None
NCT02167256	AZD0530 (100 or 125 mg daily)	Src family kinases (SFKs)-Fyn^614^	Alzheimer’s disease	phase II	Completed	None
NCT01699711	Epigallocatechin-3gallate	Dyrk1A and APP^620^	Down syndrome	phase II	Completed	Dietary supplement
NCT Number	Compound	Target	Conditions	Phase	Status	Combination Therapy
NCT05189106	Baricitinib	JAK1/2^621^	Amyotrophic lateral sclerosis Alzheimer disease Mild cognitive impairment	phase I/II	Recruiting	None
NCT03655236	K0706	Abl Tyrosine kinase^364^	Early Parkinson’s disease	phase II	Recruiting	None
NCT00095355	Lithium	Tyrosine kinase and ERK^627^	Huntington’s disease	phase II	Completed	Divalproex
NCT03792490	Fasudil	Rho kinase (ROCK)^556,557^	Amyotrophic lateral sclerosis	phase II	Completed	None
NCT05218668	WP-0512	ROCK^556,557^	Amyotrophic lateral sclerosis	phase II	Active, not recruiting	None
NCT01935518	Fasudil	ROCK^556,557^	Amyotrophic lateral sclerosis	phase II	Unknown status	None
NCT03127267	Masitinib (6.0) Masitinib (4.5)	Tyrosine kinase^635^	Amyotrophic lateral sclerosis	phase III	Recruiting	Riluzole
NCT04326283	Trametinib (0.5 mg) Trametinib (1 mg)	MEK^640^	Amyotrophic lateral sclerosis	phase I/II	Recruiting	Riluzole
NCT05105958	Tideglusib (NP031112) (1000 mg/day)	GSK3β^646^	Amyotrophic lateral sclerosis	phase II	Not yet recruiting	None
NCT03932669	Nilotinib	Bcr-Abl^575^	Spinocerebellar ataxia	phase II	Completed	None
NCT06065046	Baricitinib (4 mg)	JAK1 and JAK2^581^	Traumatic brain injury	phase II	Not yet recruiting	Standard treatment

**Table 2:** Structures of drugs related to Table 1 (can be found at https://pubchem.ncbi.nlm.nih.gov/)

				
MW150	VX-745 Neflamapimod	NP031112	Lithium Carbonate	Masitinib
				
Nilotinib	Bryostatin 1	AZD0530	Epigallocatechin-3gallate	Baricitinib
K0706	Fasudil (WP-0512)	Trametinib		

### AD

#### p38α MAPK inhibitors

MW01-18-150SRM (MW150), an isoform-selective inhibitor of p38α MAPK, effectively rescues hippocampal-dependent associative and spatial memory deficits in two AD mouse models.^583^ Subsequently, in the APP/PS1 knockin mouse model, effective MW150 administration selectively downregulates neuroinflammatory responses associated with pathological progression, without altering the physiological function of microglia.^584^ Selectively inhibiting stress-activated p38α MAPK with MW150 attenuates entorhinal cortex dysfunction associated with neuroinflammation early in AD progression.^585^ Dysregulated LTP in 2-month-old AD mice was restored by MW150 treatment, with efficacy comparable to that of widely used multi-kinase inhibitor SB203580,^585^ avoiding the limitations of using isoform-unspecific inhibitor drugs of p38 MAPK. These data have provided preliminary clinical results supporting p38α MAPK-directed drug development, and a phase II clinical trial of MW150 is underway (ClinicalTrials.gov identifier: NCT05194163).

VX-745 (also known as neflamapimod) is a novel selective p38α kinase inhibitor with excellent pharmacokinetic characteristics and in vivo activity in inflammation models.^586^ The main difference between VX-745 and MW150 is that VX-745 exhibits potent, BBB permeable, and highly selective inhibition of p38α, and has no inhibition of p38γ. Phase IIa clinical evidence in patients with early AD suggested that 6-12 weeks of VX-745 treatment significantly improves episodic memory, supporting its potential use in human AD.^587^ Oral intake of VX-745 in early AD patients attenuated Aβ production and improved episodic memory.^588^ These two clinical trials investigated the effects of VX-745 treatment (40 mg or 150 mg twice daily) for episodic memory improvement and Aβ production in AD patients for 6 to 12 weeks, which are relatively short period of time for AD trials. Therefore, a phase II clinical study of the multi-center, randomized, double-blind, placebo-controlled trial of VX-745 was conducted between December 29, 2017, and June 17, 2019, and it concluded that 24 weeks of VX-745 (40 mg, twice daily) treatment did not improve episodic memory in patients with mild AD compared to placebo, although the drug resulted in significant reductions of CSF total tau and p-tau 181 levels (ClinicalTrials.gov identifier: NCT03761849). The follow-up pharmacokinetic-pharmacodynamic analysis suggested that further studies of longer-term and higher doses of VX-745 treatment are needed to assess the effects on AD progression.^589^

#### GSK3 inhibitors

NP031112 (Tideglusib), a non-ATP competitive inhibitor of GSK3β, is a thiazolidinedione compound with anti-inflammatory and neuroprotective effects.^590,591^ In rats, injection of NP031112 into the hippocampus significantly reduced inflammation and hippocampal damages induced by kainic acid, with neuroprotective effects involving the activation of peroxisome proliferator-activated receptor-γ.^592^ Considering the potential beneficial effect of GSK3β inhibition for AD, a pilot human study was conducted in 2013 on 30 patients with mild to moderate AD, indicating a positive benefit in the NP031112 treated group compared to the placebo in multifaceted evaluations including Mini-Mental State Examination, AD Assessment Scale-Cognitive subscale, Geriatric Depression Scale, and Global Clinical Assessment, providing critical safety and efficacy evaluation for the use of NP031112 in AD. However, due to the small sample size and dose escalation method, although there are positive trends in clinical indications (including Mini-Mental State Examination, AD Assessment Scale-Cognitive subscale, Vocabulary Fluency, Geriatric Depression Scale, and final Global Clinical Assessment), it lacked statistical significance to support or reject the benefits of NP031112 in AD.^593^ A second study conducted in 2014 on 306 AD patients who received 26 weeks of NP031112 or placebo treatment reported that short-term (26 weeks) NP031112 treatment was safe and well-tolerated, but did not offer clinical benefit.^594^ Additionally, no increase in AchE activity was observed with NP031112 in the CSF of AD patients, which is disappointing because AChE co-localizes with hyperphosphorylated tau in NFTs, and inhibition of GSK3β is expected to preserve the AchE activity.^595^

Lithium is another GSK3β inhibitor that has demonstrated promising effects in animal models of AD. In PDAPP (APP-V717F) transgenic mice, lithium treatment reduces plaque load by suppressing APP processing and eliminating GSK3β-mediated Aβ synthesis in the brain.^596,597^ In addition, oral treatment with lithium carbonate can prevent NFT formation but does not improve motor or working memory deficits in aged animals.^598^ Lithium carbonate also prevents object recognition memory impairment caused by Aβ_25-35_ in rats.^599^ Moreover, chronic lithium therapy in 3xTg-AD mice showed dose-dependent improvement in brain inflammation and oxidative stress.^600^ Low doses of novel lithium salicylate proline ion co-crystal, lithium carbonate, and lithium salicylate prevented spatial cognitive decline and depressive-like behavior in APPswe/PS1dE9 mice, while lithium salicylate proline ion co-crystal also rescued hippocampus-dependent associative memory decline and irritability.^601^ Consistently, long-term lithium therapy in AD patients is associated with a lower incidence of AD and increased BDNF activity, suggesting a role of lithium in preventing early-stage AD patients at risk from deterioration.^602^ A study using [^18^F]FDG-PET imaging shows that long-term lithium therapy can reduce glucose metabolism in the cerebellum and hippocampus of non-demented elderly individuals.^603^ However, a multicenter, short-term (10-week) lithium treatment clinical trial in 71 patients with mild AD did not find an effect of lithium treatment on GSK-3 activity or CSF-based biomarker concentrations (Controlled-Trials.com identifier: ISRCTN72046462).^604^ Despite the long-term lithium therapy may have beneficial effects in AD animal models or patients, there are also studies demonstrating that lithium-induced Aβ elevation may not be related to GSK3β inhibition,^605,606^ and the neuronal protection provided by lithium under tau pathology is independent of GSK3β.^606,607^ It was also pointed out that lithium treatment also reduces tau in cell culture and mouse brain, and chronic treatment may have the side-effect of tau-dependent iron accumulation demonstrated by MRI imaging analysis.^608^ Therefore, targetting a multi-functional kinase such as GSK3β may not be the primary choice of drug target for AD.

#### Tyrosine kinase inhibitors

Two prominent tyrosine kinase inhibitors, masitinib, and nilotinib, have been developed to alleviate AD symptoms. Masitinib specifically targets mast cells and microglia activity in the neuroimmune system, and may improve AD symptoms by preventing the cell cycle re-entry, improving neuronal plasticity, inhibiting tau phosphorylation, and modulating NMDA receptors by blocking Fyn kinase activity and activation of mast cells and microglia.^609^ In non-neuronal cells, nilotinib treatment also effectively stimulates the activation of microglia and the proliferation of astrocytes, promotes Aβ clearance, and modulates immune responses in early-stage AD.^610^

A previous phase II study (ClinicalTrials.gov identifier: NCT00976118) demonstrated that masitinib considerably improves cognitive decline in patients with mild to moderate AD, offering guidance to larger clinical trials.^611^ Another completed phase IIb/III study (ClinicalTrials.gov identifier: NCT01872598) showed that masitinib (4.5 mg/kg/day) as an adjunct to cholinesterase inhibitors memantine may benefit patients with mild-to-moderate AD.^612^ In a completed phase II trial (ClinicalTrials.gov identifier: NCT02947893), compared to the placebo group, the nilotinib group exhibits significantly decreased Aβ load in the frontal lobe of patients with mild-to-moderate dementia due to AD, Aβ40, and Aβ42 in the CSF were reduced at 6 and 12 months after treatment, respectively. Additionally, hippocampal volume was larger and pTau-181 levels in CSF were lower in the nilotinib group than that in the placebo group.^613^ These trials provide strong prospects for the upcoming phase III clinical trial to evaluate the effectiveness and safety of masitinib and nilotinib in early-stage AD.

AZD0530, an inhibitor of Src family kinases including Fyn, is developed to treat mild to moderate AD. In P301S transgenic mice, chronic AZD0530 treatment prevents spatial memory and passive avoidance learning deficits, accompanied by reduced accumulation of phosphorylated tau in the hippocampus, decreased glial activation, and restoration of presynaptic markers indicative of notable neuroprotection.^614^ A phase Ib multicenter, randomized, double-blind, placebo-controlled trial (ClinicalTrials.gov identifier: NCT01864655) lasted 4 weeks with 24 subjects and was conducted using escalating doses (50 mg, 100 mg, 125 mg per day) of AZD0530, and demonstrated that an oral dose of 100-125 mg achieved significant CNS penetration in patients with mild to moderate AD, with good safety and tolerability. Subsequently, a larger phase IIa clinical trial was conducted to evaluate whether AZD0530 treatment could slow down the decline in the cerebral metabolic rate of glucose (CMRgl), as well as its safety and tolerability. However, no significant effects on relative CMRgl in AD-related regions of interest were observed. Secondary volumetric magnetic resonance imaging analysis showed no therapeutic protective effects of AZD0530 on total brain volume or ventricular volume, but did indicate a trend of slowing down the reduction of hippocampal volume and olfactory thickness. Therefore, targeting the Fyn kinase may be further evaluated as a therapeutic strategy for AD.

#### Other kinase modulators

Other kinase modulators have been investigated for AD treatment, with targets including PKC activators, Dyrk1A modulators, and JAK1/2 inhibitors. Bryostatin 1 activates PKCε by binding to the C1A and C1B domains of both conventional and novel PKC subtypes, which leads to increased levels of BDNF and PSD95, reversed synaptic loss, and accelerated synaptic maturation in AD mouse models.^615^ Additionally, bryostatin 1 enhanced synaptogenesis in cortical neurons while reducing dendritic spine density in a PKC-dependent manner.^616^ Elevated levels of PKCε during the first 24 weeks in one expanded access patient trial (ClinicalTrials.gov identifier: NCT02221947) were closely associated with cognitive benefits indicated by Mini-Mental State Examination and ADCS-ADL psychometrics.^617^ These findings offer preliminary support for bryostatin 1 as a potential therapeutic drug for AD.

Epigallocatechin-3-gallate (EGCG) is the predominant catechin present in green tea and has been shown to attenuate the excessive expression of Dyrk1A and APP in the brains of Down syndrome (DS) mouse model.^618^ Clinical trials have demonstrated that a 12-month intervention with EGCG and cognitive training yields significant improvements in visual recognition memory, inhibitory control, and adaptive behavior compared to placebo-alone and cognitive training-alone groups in DS patients.^619^ Given the high-degree association between DS and AD, subsequent preclinical investigations using the combination of EGCG with docosahexaenoic acid and α-lipoic acid have demonstrated potent anti-inflammatory and neuroprotective effects in a mouse model of AD, highlighting a protective role of EGCG from a daily diet in reducing the risk of AD.^620^ Nevertheless, it should be noted that EGCG is also an antioxidant and thus the effects may not necessarily be related to its kinase activity.

JAK1/2 inhibitor baricitinib, an anti-inflammatory drug used to treat rheumatoid arthritis,^621^ rescues inflammatory biomarkers and attenuates cell death in a dose-dependent manner in vitro.^622,623^ A recent phase I/II clinical trial (ClinicalTrials.gov identifier: NCT05189106) targeting mild cognitive impairment, AD, and ALS has commenced to investigate the penetration of baricitinib into CSF at daily doses of 2 mg or 4 mg and the effects on inflammatory biomarkers in the CSF of patients at risk of AD or with ALS. It will be concluded by June 2025.

### PD, SCAs, and HD

There are fewer kinase inhibitors entering phase II-III stages of clinical trials for PD, and completed and ongoing ones primarily assess c-Abl tyrosine kinase inhibitors such as nilotinib and vodobatinib (K0706). However, in clinical practice, nilotinib at the maximal permissible dose for 1 to 6 months has limited penetration into the CNS, resulting in minimal improvement in symptoms and no improvement in cognitive impairment in PD patients.^624^ In contrast, a more specific c-Abl inhibitor, K0706, has shown higher affinity with c-Abl in vitro than nilotinib. When orally administered to healthy volunteers and PD patients for 7 and 14 days, respectively, vodobatinib indicated better CNS penetration in PD patients than nilotinib, and more importantly, a significantly higher inhibitory potential on c-Abl is observed with vodobatinib in the brain, which was also indicated by the CSF pharmacokinetic curve of drugs, showing the same conclusion of better neuroprotective effects of vodobatinib.^624^ A phase II clinical trial evaluating the safety and efficacy of K0706 in early-stage PD patients has commenced the recruitment phase (ClinicalTrials.gov identifier: NCT03655236). Currently, a phase II clinical trial evaluating the efficacy and safety of nilotinib for 1 year has been completed in patients with autosomal dominant spinocerebellar ataxia (ClinicalTrials.gov identifier: NCT03932669). The data from this study suggest that nilotinib may improve the severity of ataxia in patients with autosomal dominant spinocerebellar ataxia, and serum protein markers (including leucine-rich α-2-glycoprotein, vitamin D binding protein, and C4b binding protein β and α chain) may be clues to predict response to nilotinib.^625^

Similarly, limited kinase-targeted drugs are tested clinically for HD. Since the low levels of BDNF in the brain and CSF of HD patients may account for the disruption of the cortical-striatal circuit and disease progression, kinase-related drugs responsible for BDNF regulation or improving vesicular transport or expression levels of BDNF may be a viable approach.^626,627^ In line with this, a phase II clinical trial (ClinicalTrials.gov identifier: NCT00095355) initiated in 2004 has investigated the effects of lithium carbonate and sodium valproate (primarily used for mood disorders and epileptic seizures) on BDNF expression in CSF of HD patients, and whether TKs and ERK signaling pathways are involved, although the final report was not released after the study was completed in 2005. The protein expression and function of CREB and BDNF are abnormal in the brains of HD patients. Phosphodiesterase inhibition restored the protein levels of CREB and BDNF in the brain of the R6/2 transgenic mouse model, thereby reducing the degeneration of the striatum and cortex.^628^ Therefore, the primary focus of HD-related clinical trials later turned to the efficacy and safety of phosphodiesterase inhibitors. For example, a positron emission tomography clinical trial of rolipram, a selective phosphodiesterase 4 inhibitor, began in 2011 (ClinicalTrials.gov identifier: NCT01602900), but the results have not yet been published.

### ALS and CTE

#### ROCK inhibitor

Fasudil, which effectively targets ROCK, is proven to be a potential drug for the treatment of ALS. In 2013, oral doses of fasudil at 30 or 100 mg/kg were administered to SOD1^G93A^ mice, and fasudil reduced ROCK activity and tensin homolog expression, increased p-AKT levels, and thereby prevented the death of motor neurons and mitigated the disease progression.^556^ Intriguingly, the release of pro-inflammatory factors tumor necrosis factor-α, interleukin-6, and chemokines such as C-C motif ligand 2, C-C motif ligand 3, and C-C motif ligand 5 was consistently decreased by fasudil in the spinal cord of SOD1^G93A^ mice. Fasudil also facilitated the process of neuromuscular junction by remodeling the motor axons of the sciatic nerve. It notably improved the motor behavior of male SOD1^G93A^ mice with good tolerance, likely due to the higher ROCK activity present in the male mice at the advanced stage of ALS receiving better drug response.^629^ Taken together, although firstly approved for vasospasm, fasudil exerts great therapeutic potential in treating ALS.

The first clinical trial (ClinicalTrials.gov identifier: NCT01935518), initiated in September 2013 aiming to assess the safety and effectiveness of fasudil in ALS patients, was concluded without releasing the results. In addition, a phase IIa clinical trial, known as the ROCK-ALS trial (initiated recruitment in 2019 from 16 centers across Germany, France, and Switzerland. ClinicalTrials.gov identifier: NCT03792490), was proposed to evaluate the safety, tolerance, and efficacy of fasudil as an intervention in early-stage ALS patients, encompassing a total of 120 individuals suspected or diagnosed with ALS within 6-18 months of experiencing muscle weakness, and the results of this study have shown that fasudil is well tolerated and safe in patients with ALS.^630,631^ In March 2020, the compassionate use of fasudil was reported in three ALS patients, a 66-year-old male and two females aged 62 and 68, indicating well tolerance.^632^ Overall, fasudil has shown promising effects in ALS animal models, and the clinical benefits have been demonstrated in trials.

#### RIPK1 inhibitors

Since RIPK1 is essential in cell death decisions, including necroptosis and RIPK1-dependent apoptosis, its inhibitor has been tested in several disease models including ALS. Inhibition of RIPK1 prevents oligodendrocyte degeneration that precedes the onset of motor dysfunction in SOD^G93A^ transgenic mice.^71^ GSK2982772, a type III inhibitor of RIPK1, is being developed for the treatment of chronic inflammatory diseases. A phase I safety trial of oral safety, tolerability, pharmacokinetics, and exploratory pharmacodynamics of GSK2982772 has been completed in healthy male volunteers. The results showed that single and repeated doses of oral GSK2982772 are safe and well tolerated^633^ (ClinicalTrials.gov identifier: NCT02302404). Another brain-penetrant small molecule inhibitor of RIPK1 is DNL747, also known as SAR443060 (reducing RIPK1 phosphorylation at Ser166), which has also completed phase I safety, tolerability, pharmacokinetics, and pharmacodynamics trials in AD or ALS patients (ClinicalTrials.gov identifier: NCT03757325, NCT03757351). The results showed that DNL747 was safe and well tolerated in AD and ALS patients, exhibited good blood-brain barrier permeability after oral administration, and had good peripheral target binding ability,^634^ which brings potential therapeutic prospects for AD, ALS, and other neurodegenerative diseases. In addition, DNL788 (also known as SAR443820), as a potent, selective, brain-penetrating RIPK1 small molecule inhibitor, showed good safety, pharmacokinetics, and strong interaction with the target in a phase I clinical trial conducted in healthy adult participants (ClinicalTrials.gov identifier: NCT05795907). Subsequently, DNL788 entered phase II clinical trials for the treatment of ALS in 2022 (ClinicalTrials.gov identifier: NCT05237284). Unfortunately, Denali recently stated in a document submitted to the U.S. Securities and Exchange Commission that the phase II Himalaya trial of DNL788 failed to reach the primary endpoint of the ALS-Functional Rating Scale (https://alsnewstoday.com/news/denali-therapy-candidate-fails-slowing-als-progression-trial/), where the official clinical trial data has not yet been released. Therefore, the clinical drug research targeting RIPK1 for the treatment of ALS still encounters significant challenges.

#### Other kinase inhibitors

Other potential kinase inhibitors tested for ALS include TK inhibitors (such as JAK inhibitors) and MEK inhibitors that downregulate the MAPK/ERK pathway. Highly selective TK inhibitor masitinib exhibits considerable preventive effects against CNS neuroinflammation in ALS, stroke, and AD. Neuronal death and rapid progression of paralysis involve abnormal proliferation of spinal cord microglial cells and activation of astrocytes in the SOD1^G93A^ rat, while oral administration of masitinib after the onset of paralysis reduced these pathologies. Noteworthily, masitinib treatment initiated 7 days after the onset of paralysis extended post-paralysis survival by 40%.^635^ In vitro, masitinib also effectively inhibits colony-stimulating factor-induced proliferation, cell migration, and expression of inflammatory mediators in cultured spinal cord microglial cells.^635,636^ These preclinical findings provide compelling biological evidence supporting the use of masitinib for ALS-related neuroinflammation.

A randomized phase III clinical trial AB10015 (ClinicalTrials.gov identifier: NCT02588677) has investigated masitinib as an adjunct therapy to riluzole in ALS patients. In this trial, 394 patients were assigned in a 1:1:1 ratio to receive either riluzole (100 mg/day) plus placebo, or masitinib at doses of 4.5 or 3.0 mg/kg/day. A higher dose of masitinib was well-tolerated and effectively decelerated the rate of functional decline in ALS patients, as assessed by the ALS-Functional Rating Scale-Revised.^637^ Subsequently, an evaluation of long-term overall survival data was conducted for all participants in the AB10015 study, suggesting that initiating oral administration of masitinib (4.5 mg/kg/day) before severe impairment of neuromuscular function can prolong patient survival by over 2 years compared to that of the placebo.^638^ A confirmatory phase III clinical trial to validate the efficacy and safety of masitinib versus placebo in combination with riluzole for ALS treatment was initiated in 2021 (ClinicalTrials.gov identifier: NCT03127267) and is currently in the recruitment phase.

JAK inhibitors proven to be potent anti-inflammatory drugs include tofacitinib (inhibiting JAK1/2/3), baricitinib (inhibiting JAK1/2), and upadacitinib (selectively inhibiting JAK1), all of which have received FDA approval for treating rheumatoid arthritis. However, the clinical investigation of JAK inhibitors for the treatment of neurodegenerative diseases is still in the early stages. A phase I/II clinical trial has been initiated to assess whether baricitinib (at doses of 2 or 4 mg/day) could achieve therapeutic concentration in the CSF and inhibit type I interferon-related inflammatory biomarkers in ALS, AD, and mild cognitive impairment patients, with an estimated completion in June 2025 (ClinicalTrials.gov identifier: NCT05189106). In addition to ALS and AD, a multicenter randomized controlled phase II clinical trial studying the therapeutic effect of baricitinib (given daily at the dosage of 4 mg) on patients with moderate to severe traumatic intracerebral hemorrhage/cerebral contusion is also being planned (the current status is not yet recruiting), and is expected to be completed in December 2025 (ClinicalTrials.gov identifier: NCT06065046).

Trametinib (GSK1120212, SNR1611) is a selective MEK1/2 inhibitor approved for the clinical treatment of metastatic melanoma with *BRAF* V600E or V600K mutations.^639,640^ A phase I/II clinical trial evaluating the safety, tolerability, and efficacy of trametinib in ALS patients was initiated in 2020 (ClinicalTrials.gov identifier: NCT04326283), aiming to assess whether suppressing the MAPK/ERK pathway by trametinib is of benefit for ALS, and the trial is currently recruiting participants.

## Concluding remarks

Protein kinases play diverse roles in signal pathways engaged in cellular metabolism, cell growth, and neuroinflammation, positioning them as central targets for treating neurodegenerative diseases. However, the intricate network of protein kinase-protein interactions and mechanisms of action have limited the thorough understanding of their functions, inhibitors, and activators in diseases. Extensive research has been focusing on unraveling the unique roles and regulatory functions of different protein kinases in specific neurodegenerative diseases.

The inter-regulatory network constructed by diverse protein kinases such as GSK3β, CDK5, CK1, PKA, p38 MAPK, Fyn, TTBK1, AMPK, and others serves as an imperative hub to guide the pathogenesis and progression of AD. In the AD brain, abnormal protein hydrolysis of p35-CDK5 by Aβ, Ca^2+^, and calpain-1 leads to the generation of p25-CDK5, which in turn activates the cell cycle re-entry, mitochondrial dysfunction, and apoptosis. Elevated CK1 activity leads to the excessive activation of GSK3β and CDK5, and the former further triggers Fyn activation, exacerbating neuronal excitotoxicity and neural network function, and ultimately contributing to neurodegeneration in AD. Additionally, AMPK, by inhibiting mTOR activity, promotes autophagy and reduces Aβ toxicity. The reduced PKA activity in AD results in decreased expression of CREB/BDNF and SIRT1, giving rise to enhanced Aβ production and impaired synaptic plasticity and memory function. Furthermore, increased activity of p38 MAPK is mainly responsible for the neuroinflammation and oxidative stress in AD. Therefore, the predominant focus is being put on p38α MAPK, GSK3, and tyrosine kinases in the therapeutic landscape for AD, and the majority of these therapies are still in phase I/II clinical trials.

mHTT pathogenesis is notably accelerated by a signaling network that engages AKT, ERK, p38, JNK, CDK5, and IKKβ. Conversely, these kinase signaling cascades are inevitably influenced by the action of abnormal mHTT. For instance, mHTT aggregation inhibits the upstream PI3K/AKT or the ERK/CREB signaling, which are proven to be neuroprotective. Alternatively, mHTT activates the IKKβ/NF-κB pathway and promotes inflammatory signaling, in addition to p38 activation-induced cell apoptosis. Furthermore, MEK/ERK activation by mHTT enhances HTT phosphorylation, thereby disrupting the transport and uptake of neurotransmitter glutamate. While fALS only accounts for a very small percentage of ALS, the majority of sporadic ALS cases with indefinable causes are the principal challenges for clinical treatment. Etiological studies of ALS have highlighted the importance of a homeostatic environment regulated by kinase networks. Clinical trials targeting ROCK, RIPK1, and MEK in ALS are currently in phase I/II, and tyrosine kinase inhibitors have reached phase III. However, due to the complexity of protein kinase signaling networks, modifying the activity of one kinase in purpose may yield broad outcomes from various respects in distinct neurodegenerative diseases, possibly resulting from interferences of the dynamics of its interacting kinases, therefore disruption of physiological processes by such inhibitor may also result in potential side effects.^641^

It is worth noting that most neurodegenerative diseases are related to family genetic inheritance. Although gene expression is related to the environment, genes determine the phenotype to a large extent. Therefore, variations in kinase genes may be another important reason that affects kinase function, leads to disease and affects pathological progression. In AD cohort studies, it has been confirmed that a single nucleotide polymorphism (rs2651206) located in the *TTBK1* gene may promote the occurrence of sporadic late-onset AD in the Chinese Han population.^285^ SNPs in nucleotides within the functional domain of *LRRK2* have been identified that cause common and sporadic forms of PD, including G2019S (the most common and inherited in an autosomal dominant manner), I2020T, N1437H, R1441C, R1441G, I1441H, and Y1699C, these variants all lead to increased LRRK2 kinase activity.^63^ On the contrary, *LRRK2* also has protective variants in PD (such as R1398H and N551K) that reduce LRRK2 activity,^642^ which suggests that kinase gene mutations have two sides in disease progression and maintaining wild-type LRRK2 kinase activity is not necessarily optimal in PD treatment. *NEK1* variants include frameshift variants (p.Glu929Asnfs*12) and missense variants (p.Val713Met, p.Ser909Cys and p.Arg1073Cys), all of which reduce the protein level of NEK1 in fibroblasts from ALS patients, leading to a partial loss of NEK1 function and promoting the progression of ALS.^643^ *TBK1* site mutation (R228H) causes neurodegeneration by reducing TBK1 activity, thereby increasing the course of frontotemporal dementia-ALS.^644^ Gene sequence analysis of population cohort data showed that missense mutations (Gln127Arg) in the *PRKCG* gene may cause the occurrence of SCA14^645^ (disease-related variations in genes can be found at https://rddc.tsinghua-gd.org/). Similarly, mutations in non-kinase genes such as *TARDBP*, *mHTT*, and *OPTN* may also promote/reduce kinase activity by regulating abnormal protein expression, thereby unbalancing protein homeostasis and promoting the occurrence of neurodegenerative diseases. Therefore, when performing gene therapy or ASO therapy on kinase genes, it may be necessary to consider the physiological function of the kinase.

In summary, kinase signaling networks play a double-edged sword role in the progression of neurodegenerative diseases and are closely linked to neurodegenerative lesions advance. When developing kinase-targeted drugs and formulating dosing strategies, in addition to physiological barriers in the CNS like BBB and the blood-CSF barrier, factors such as the drug delivery window, chronic inflammation, and acute immune activation may also need to be considered. While the link between kinase regulatory networks and neurodegenerative diseases remains incompletely understood, researchers are making steady progress in developing better drugs targeting protein kinases and more efficient delivery techniques directed to the CNS. Targeted kinase therapy for neurodegenerative diseases continues to hold potential and challenges simultaneously.

## References

[1] Roskoski, R. Jr A historical overview of protein kinases and their targeted small molecule inhibitors. *Pharm. Res.* **100**, 1–23 (2015). 10.1016/j.phrs.2015.07.010 26207888

[2] Attwood, M. M. et al. Trends in kinase drug discovery: targets, indications and inhibitor design. *Nat. Rev. Drug Discov.* **20**, 839–861 (2021). 34354255 10.1038/s41573-021-00252-y

[3] Manning, G. et al. The protein kinase complement of the human genome. *Science* **298**, 1912–1934 (2002). 12471243 10.1126/science.1075762

[4] Zhang, H. et al. A subcellular map of the human kinome. *Elife*. **10**, e64943 (2021). 10.7554/eLife.64943 PMC8175086 33988507

[5] Klaeger, S. et al. The target landscape of clinical kinase drugs. *Science* **358**, eaan4368 (2017). 10.1126/science.aan4368 PMC6542668 29191878

[6] Liu, W. S. et al. Plasma proteomics identify biomarkers and undulating changes of brain aging. *Nat Aging* **5**, 99–112 (2024). 10.1038/s43587-024-00753-6 39653801

[7] Nandi, A. et al. Global and regional projections of the economic burden of Alzheimer’s disease and related dementias from 2019 to 2050: a value of statistical life approach. *EClinicalMedicine* **51**, 101580 (2022). 35898316 10.1016/j.eclinm.2022.101580 PMC9310134

[8] Budd Haeberlein, S. et al. Two randomized phase 3 studies of aducanumab in early Alzheimer’s disease. *J. Prev. Alzheimers Dis.* **9**, 197–210 (2022). 35542991 10.14283/jpad.2022.30

[9] van Dyck, C. H. et al. Lecanemab in early Alzheimer’s Disease. *N. Engl. J. Med.* **388**, 9–21 (2023). 36449413 10.1056/NEJMoa2212948

[10] Sims, J. R. et al. Donanemab in early symptomatic Alzheimer Disease: the TRAILBLAZER-ALZ 2 randomized clinical trial. *JAMA* **330**, 512–527 (2023). 37459141 10.1001/jama.2023.13239 PMC10352931

[11] Mummery, C. J. et al. Tau-targeting antisense oligonucleotide MAPT(Rx) in mild Alzheimer’s disease: a phase 1b, randomized, placebo-controlled trial. *Nat. Med.* **29**, 1437–1447 (2023). 37095250 10.1038/s41591-023-02326-3 PMC10287562

[12] Bejanin, A. & Villain, N. Posterior cortical atrophy: new insights into treatments and biomarkers for Alzheimer’s disease. *Lancet Neurol.* **23**, 127–128 (2024). 38267172 10.1016/S1474-4422(23)00501-X

[13] Congdon, E. E. et al. Tau-targeting therapies for Alzheimer disease: current status and future directions. *Nat. Rev. Neurol.* **19**, 715–736 (2023). 37875627 10.1038/s41582-023-00883-2 PMC10965012

[14] Lang, A. E. et al. Trial of cinpanemab in early Parkinson’s Disease. *N. Engl. J. Med.* **387**, 408–420 (2022). 35921450 10.1056/NEJMoa2203395

[15] Pagano, G. et al. Trial of prasinezumab in early-stage Parkinson’s Disease. *N. Engl. J. Med.* **387**, 421–432 (2022). 35921451 10.1056/NEJMoa2202867

[16] Buur, L. et al. Randomized phase I trial of the alpha-synuclein antibody Lu AF82422. *Mov. Disord*. **39**, 936–944 (2024). 10.1002/mds.29784 38494847

[17] Sengupta, U. & Kayed, R. Amyloid beta, Tau, and alpha-Synuclein aggregates in the pathogenesis, prognosis, and therapeutics for neurodegenerative diseases. *Prog. Neurobiol.* **214**, 102270 (2022). 35447272 10.1016/j.pneurobio.2022.102270

[18] Paganoni, S. et al. Trial of sodium phenylbutyrate-taurursodiol for amyotrophic lateral sclerosis. *N. Engl. J. Med.* **383**, 919–930 (2020). 32877582 10.1056/NEJMoa1916945 PMC9134321

[19] Jaiswal, M. K. Riluzole and edaravone: a tale of two amyotrophic lateral sclerosis drugs. *Med Res Rev.* **39**, 733–748 (2019). 30101496 10.1002/med.21528

[20] Miller, T. M. et al. An antisense oligonucleotide against SOD1 delivered intrathecally for patients with SOD1 familial amyotrophic lateral sclerosis: a phase 1, randomised, first-in-man study. *Lancet Neurol.* **12**, 435–442 (2013). 23541756 10.1016/S1474-4422(13)70061-9 PMC3712285

[21] Blair, H. A. Tofersen: first approval. *Drugs* **83**, 1039–1043 (2023). 37316681 10.1007/s40265-023-01904-6

[22] van den Berg, L. H. et al. Safety, tolerability, and pharmacokinetics of antisense oligonucleotide BIIB078 in adults with C9orf72-associated amyotrophic lateral sclerosis: a phase 1, randomised, double blinded, placebo-controlled, multiple ascending dose study. *Lancet Neurol.* **23**, 901–912 (2024). 39059407 10.1016/S1474-4422(24)00216-3

[23] Kim, G. et al. Genome-wide CRISPR screen reveals v-ATPase as a drug target to lower levels of ALS protein ataxin-2. *Cell Rep.* **41**, 111508 (2022). 36288714 10.1016/j.celrep.2022.111508 PMC9664452

[24] Sengupta, A. et al. Phosphorylation of tau at both Thr 231 and Ser 262 is required for maximal inhibition of its binding to microtubules. *Arch. Biochem. Biophys.* **357**, 299–309 (1998). 9735171 10.1006/abbi.1998.0813

[25] Parra Bravo, C., Naguib, S. A. & Gan, L. Cellular and pathological functions of tau. *Nat. Rev. Mol. Cell Biol.* **25**, 845–864 (2024). 10.1038/s41580-024-00753-9 39014245

[26] Mangiafico, S. P. et al. Tau suppresses microtubule-regulated pancreatic insulin secretion. *Mol. Psychiatr.* **28**, 3982–3993 (2023). 10.1038/s41380-023-02267-w 37735502

[27] Johnson, G. V. & Stoothoff, W. H. Tau phosphorylation in neuronal cell function and dysfunction. *J. Cell Sci.* **117**, 5721–5729 (2004). 15537830 10.1242/jcs.01558

[28] Ramalingam, N. & Dettmer, U. alpha-Synuclein serine129 phosphorylation - the physiology of pathology. *Mol. Neurodegener.* **18**, 84 (2023). 37953316 10.1186/s13024-023-00680-x PMC10641962

[29] Neumann, M. et al. Phosphorylation of S409/410 of TDP-43 is a consistent feature in all sporadic and familial forms of TDP-43 proteinopathies. *Acta Neuropathol.* **117**, 137–149 (2009). 19125255 10.1007/s00401-008-0477-9 PMC2693625

[30] Heras-Sandoval, D., Perez-Rojas, J. M., Hernandez-Damian, J. & Pedraza-Chaverri, J. The role of PI3K/AKT/mTOR pathway in the modulation of autophagy and the clearance of protein aggregates in neurodegeneration. *Cell Signal* **26**, 2694–2701 (2014). 25173700 10.1016/j.cellsig.2014.08.019

[31] Muraleedharan, R. & Dasgupta, B. AMPK in the brain: its roles in glucose and neural metabolism. *FEBS J.* **289**, 2247–2262 (2022). 34355526 10.1111/febs.16151

[32] Ding, C., Wu, Y., Dabas, H. & Hammarlund, M. Activation of the CaMKII-Sarm1-ASK1-p38 MAP kinase pathway protects against axon degeneration caused by loss of mitochondria. *Elife*. **11** (2022). 10.7554/eLife.73557 PMC8920508 35285800

[33] Hara, S. et al. Serine 129 phosphorylation of membrane-associated alpha-synuclein modulates dopamine transporter function in a G protein-coupled receptor kinase-dependent manner. *Mol. Biol. Cell* **24**, 1649–1660 (2013). S1641-1643. 23576548 10.1091/mbc.E12-12-0903 PMC3667719

[34] Bergeron, M. et al. In vivo modulation of polo-like kinases supports a key role for PLK2 in Ser129 alpha-synuclein phosphorylation in mouse brain. *Neuroscience* **256**, 72–82 (2014). 24128992 10.1016/j.neuroscience.2013.09.061

[35] Degterev, A., Ofengeim, D. & Yuan, J. Targeting RIPK1 for the treatment of human diseases. *Proc. Natl Acad. Sci. USA* **116**, 9714–9722 (2019). 31048504 10.1073/pnas.1901179116 PMC6525537

[36] Zhao, B. et al. Targeting RACK1 to alleviate TDP-43 and FUS proteinopathy-mediated suppression of protein translation and neurodegeneration. *Acta Neuropathol. Com.* **11**, 200 (2023). 10.1186/s40478-023-01705-8 PMC10726565 38111057

[37] Tolosa, E., Vila, M., Klein, C. & Rascol, O. LRRK2 in Parkinson disease: challenges of clinical trials. *Nat. Rev. Neurol.* **16**, 97–107 (2020). 31980808 10.1038/s41582-019-0301-2

[38] Eid, S. et al. KinMap: a web-based tool for interactive navigation through human kinome data. *BMC Bioinforma.* **18**, 16 (2017). 10.1186/s12859-016-1433-7 PMC5217312 28056780

[39] Wang, B. et al. An overview of kinase downregulators and recent advances in discovery approaches. *Signal Transduct. Tar.* **6**, 423 (2021). 10.1038/s41392-021-00826-7 PMC8685278 34924565

[40] Kanev, G. K. et al. The landscape of atypical and eukaryotic protein kinases. *Trends Pharm. Sci.* **40**, 818–832 (2019). 31677919 10.1016/j.tips.2019.09.002

[41] Gomez-Puerta, J. A. & Mocsai, A. Tyrosine kinase inhibitors for the treatment of rheumatoid arthritis. *Curr. Top. Med. Chem.* **13**, 760–773 (2013). 23574525 10.2174/15680266113139990094 PMC3796894

[42] Robinson, D. R., Wu, Y. M. & Lin, S. F. The protein tyrosine kinase family of the human genome. *Oncogene* **19**, 5548–5557 (2000). 11114734 10.1038/sj.onc.1203957

[43] Lemmon, M. A. & Schlessinger, J. Cell signaling by receptor tyrosine kinases. *Cell* **141**, 1117–1134 (2010). 20602996 10.1016/j.cell.2010.06.011 PMC2914105

[44] Qi, D. et al. HO-1 attenuates hippocampal neurons injury via the activation of BDNF-TrkB-PI3K/Akt signaling pathway in stroke. *Brain Res.* **1577**, 69–76 (2014). 24997248 10.1016/j.brainres.2014.06.031

[45] Yuan, H. et al. The regulatory mechanism of neurogenesis by IGF-1 in adult mice. *Mol. Neurobiol.* **51**, 512–522 (2015). 24777577 10.1007/s12035-014-8717-6

[46] Shen, J. et al. Neurovascular coupling in the dentate gyrus regulates adult hippocampal neurogenesis. *Neuron* **103**, 878–890 e873 (2019). 31257104 10.1016/j.neuron.2019.05.045 PMC6728189

[47] Liang, S. et al. Neuroprotective profile of novel SRC kinase inhibitors in rodent models of cerebral ischemia. *J. Pharm. Exp. Ther.* **331**, 827–835 (2009). 10.1124/jpet.109.156562 19741150

[48] Lennmyr, F. et al. Src family kinase-inhibitor PP2 reduces focal ischemic brain injury. *Acta Neurol. Scand.* **110**, 175–179 (2004). 15285775 10.1111/j.1600-0404.2004.00306.x

[49] Tuo, Q. Z. et al. Thrombin induces ACSL4-dependent ferroptosis during cerebral ischemia/reperfusion. *Signal Transduct. Target Ther.* **7**, 59 (2022). 35197442 10.1038/s41392-022-00917-z PMC8866433

[50] Tuo, Q. Z. & Lei, P. Ferroptosis in ischemic stroke: animal models and mechanisms. *Zool. Res.* **45**, 1235–1248 (2024). 39397243 10.24272/j.issn.2095-8137.2024.239 PMC11668946

[51] Matrone, C., Petrillo, F., Nasso, R. & Ferretti, G. Fyn tyrosine kinase as harmonizing factor in neuronal functions and dysfunctions. *Int. J. Mol. Sci*. **21**, 4444 (2020). 10.3390/ijms21124444 PMC7352836 32580508

[52] Brown, M. T. & Cooper, J. A. Regulation, substrates and functions of src. *Biochim. Biophys. Acta* **1287**, 121–149 (1996). 8672527 10.1016/0304-419x(96)00003-0

[53] Nada, S. et al. Constitutive activation of Src family kinases in mouse embryos that lack Csk. *Cell* **73**, 1125–1135 (1993). 8513497 10.1016/0092-8674(93)90642-4

[54] Nguyen, T. H., Liu, J. & Lombroso, P. J. Striatal enriched phosphatase 61 dephosphorylates Fyn at phosphotyrosine 420. *J. Biol. Chem.* **277**, 24274–24279 (2002). 11983687 10.1074/jbc.M111683200

[55] Liebl, E. C. et al. Dosage-sensitive, reciprocal genetic interactions between the Abl tyrosine kinase and the putative GEF trio reveal trio’s role in axon pathfinding. *Neuron* **26**, 107–118 (2000). 10798396 10.1016/s0896-6273(00)81142-3

[56] Lebouvier, T. et al. The microtubule-associated protein tau is also phosphorylated on tyrosine. *J. Alzheimers Dis.* **18**, 1–9 (2009). 19542604 10.3233/JAD-2009-1116

[57] Hatami, M. et al. STAT5a and STAT6 gene expression levels in multiple sclerosis patients. *Cytokine* **106**, 108–113 (2018). 29126764 10.1016/j.cyto.2017.10.022

[58] Brooks, A. J. et al. Mechanism of activation of protein kinase JAK2 by the growth hormone receptor. *Science* **344**, 1249783 (2014). 24833397 10.1126/science.1249783

[59] Hebenstreit, D., Horejs-Hoeck, J. & Duschl, A. JAK/STAT-dependent gene regulation by cytokines. *Drug N. Perspect.* **18**, 243–249 (2005). 10.1358/dnp.2005.18.4.908658 16034480

[60] Luoqian, J. et al. Ferroptosis promotes T-cell activation-induced neurodegeneration in multiple sclerosis. *Cell Mol. Immunol.* **19**, 913–924 (2022). 35676325 10.1038/s41423-022-00883-0 PMC9338013

[61] Shen, D. et al. Genome-wide and functional analyses of tyrosine kinase-like family genes reveal potential roles in development and virulence in mosquito pathogen *Pythium guiyangense*. *Fungal Genet. Biol.* **130**, 11–18 (2019). 31022498 10.1016/j.fgb.2019.04.009

[62] Galatsis, P. Leucine-rich repeat kinase 2 inhibitors: a patent review (2014-2016). *Expert Opin. Ther. Pat.* **27**, 667–676 (2017). 28117607 10.1080/13543776.2017.1280464

[63] Myasnikov, A. et al. Structural analysis of the full-length human LRRK2. *Cell* **184**, 3519–3527 e3510 (2021). 34107286 10.1016/j.cell.2021.05.004 PMC8887629

[64] Wang, X. et al. LRRK2 regulates mitochondrial dynamics and function through direct interaction with DLP1. *Hum. Mol. Genet.* **21**, 1931–1944 (2012). 22228096 10.1093/hmg/dds003 PMC3315202

[65] Seol, W., Nam, D. & Son, I. Rab GTPases as physiological substrates of LRRK2 Kinase. *Exp. Neurobiol.* **28**, 134–145 (2019). 31138985 10.5607/en.2019.28.2.134 PMC6526114

[66] Ho, D. H. et al. LRRK2 kinase activity induces mitochondrial fission in microglia via Drp1 and modulates neuroinflammation. *Exp. Neurobiol.* **27**, 171–180 (2018). 30022868 10.5607/en.2018.27.3.171 PMC6050415

[67] Liu, J. et al. The structure of mouse RIPK1 RHIM-containing domain as a homo-amyloid and in RIPK1/RIPK3 complex. *Nat. Commun.* **15**, 6975 (2024). 39143113 10.1038/s41467-024-51303-y PMC11325021

[68] Meng, H. et al. Death-domain dimerization-mediated activation of RIPK1 controls necroptosis and RIPK1-dependent apoptosis. *Proc. Natl. Acad. Sci. USA* **115**, E2001–E2009 (2018). 29440439 10.1073/pnas.1722013115 PMC5834731

[69] Ofengeim, D. & Yuan, J. Regulation of RIP1 kinase signalling at the crossroads of inflammation and cell death. *Nat. Rev. Mol. Cell Biol.* **14**, 727–736 (2013). 24129419 10.1038/nrm3683

[70] Yuan, J., Amin, P. & Ofengeim, D. Necroptosis and RIPK1-mediated neuroinflammation in CNS diseases. *Nat. Rev. Neurosci.* **20**, 19–33 (2019). 30467385 10.1038/s41583-018-0093-1 PMC6342007

[71] Ito, Y. et al. RIPK1 mediates axonal degeneration by promoting inflammation and necroptosis in ALS. *Science* **353**, 603–608 (2016). 27493188 10.1126/science.aaf6803 PMC5444917

[72] Ofengeim, D. et al. RIPK1 mediates a disease-associated microglial response in Alzheimer’s disease. *Proc. Natl. Acad. Sci. USA* **114**, E8788–E8797 (2017). 28904096 10.1073/pnas.1714175114 PMC5642727

[73] Lule, S. et al. Genetic inhibition of receptor interacting protein kinase-1 reduces cell death and improves functional outcome after intracerebral hemorrhage in mice. *Stroke* **48**, 2549–2556 (2017). 28765287 10.1161/STROKEAHA.117.017702 PMC6366458

[74] Newton, K. et al. Activity of protein kinase RIPK3 determines whether cells die by necroptosis or apoptosis. *Science* **343**, 1357–1360 (2014). 24557836 10.1126/science.1249361

[75] Holzman, L. B., Merritt, S. E. & Fan, G. Identification, molecular cloning, and characterization of dual leucine zipper bearing kinase. A novel serine/threonine protein kinase that defines a second subfamily of mixed lineage kinases. *J. Biol. Chem.* **269**, 30808–30817 (1994). 7983011

[76] Ferraris, D., Yang, Z. & Welsbie, D. Dual leucine zipper kinase as a therapeutic target for neurodegenerative conditions. *Future Med. Chem.* **5**, 1923–1934 (2013). 24175744 10.4155/fmc.13.150

[77] Shin, J. E. et al. Dual leucine zipper kinase is required for retrograde injury signaling and axonal regeneration. *Neuron* **74**, 1015–1022 (2012). 22726832 10.1016/j.neuron.2012.04.028 PMC3383631

[78] Welsbie, D. S. et al. Enhanced functional genomic screening identifies novel mediators of dual leucine zipper kinase-dependent injury signaling in neurons. *Neuron* **94**, 1142–1154 e1146 (2017). 28641113 10.1016/j.neuron.2017.06.008 PMC5553555

[79] Hirai, S. et al. The c-Jun N-terminal kinase activator dual leucine zipper kinase regulates axon growth and neuronal migration in the developing cerebral cortex. *J. Neurosci.* **26**, 11992–12002 (2006). 17108173 10.1523/JNEUROSCI.2272-06.2006 PMC6674859

[80] Hu, X. et al. Microglial and macrophage polarization-new prospects for brain repair. *Nat. Rev. Neurol.* **11**, 56–64 (2015). 25385337 10.1038/nrneurol.2014.207 PMC4395497

[81] Liu, B. et al. Interleukin-1 receptor-associated kinase (IRAK)-M -mediated type 2 microglia polarization ameliorates the severity of experimental autoimmune encephalomyelitis (EAE). *J. Autoimmun.* **102**, 77–88 (2019). 31036429 10.1016/j.jaut.2019.04.020

[82] Miranda-Hernandez, S. & Baxter, A. G. Role of toll-like receptors in multiple sclerosis. *Am. J. Clin. Exp. Immunol.* **2**, 75–93 (2013). 23885326 PMC3714200

[83] Akhurst, R. J. & Hata, A. Targeting the TGFbeta signalling pathway in disease. *Nat. Rev. Drug Discov.* **11**, 790–811 (2012). 23000686 10.1038/nrd3810 PMC3520610

[84] Wu, X. et al. Photoactivation of TGFbeta/SMAD signaling pathway ameliorates adult hippocampal neurogenesis in Alzheimer’s disease model. *Stem Cell Res. Ther.* **12**, 345 (2021). 34116709 10.1186/s13287-021-02399-2 PMC8196501

[85] Arany, P. R. et al. Photoactivation of endogenous latent transforming growth factor-beta1 directs dental stem cell differentiation for regeneration. *Sci. Transl. Med.* **6**, 238ra269 (2014). 10.1126/scitranslmed.3008234 PMC4113395 24871130

[86] Tebben, A. J. et al. Crystal structures of apo and inhibitor-bound TGFbetaR2 kinase domain: insights into TGFbetaR isoform selectivity. *Acta Crystallogr. D Struct. Biol.* **72**, 658–674 (2016). 27139629 10.1107/S2059798316003624

[87] Varjosalo, M. et al. The protein interaction landscape of the human CMGC kinase group. *Cell Rep.* **3**, 1306–1320 (2013). 23602568 10.1016/j.celrep.2013.03.027

[88] Cheung, Z. H. & Ip, N. Y. Cdk5: a multifaceted kinase in neurodegenerative diseases. *Trends Cell Biol.* **22**, 169–175 (2012). 22189166 10.1016/j.tcb.2011.11.003

[89] Bianchetta, M. J., Lam, T. T., Jones, S. N. & Morabito, M. A. Cyclin-dependent kinase 5 regulates PSD-95 ubiquitination in neurons. *J. Neurosci.* **31**, 12029–12035 (2011). 21849563 10.1523/JNEUROSCI.2388-11.2011 PMC3190401

[90] Plattner, F. et al. Memory enhancement by targeting Cdk5 regulation of NR2B. *Neuron* **81**, 1070–1083 (2014). 24607229 10.1016/j.neuron.2014.01.022 PMC4010123

[91] Zhong, P. et al. Cyclin-dependent kinase 5 in the ventral tegmental area regulates depression-related behaviors. *J. Neurosci.* **34**, 6352–6366 (2014). 24790206 10.1523/JNEUROSCI.3673-13.2014 PMC4004818

[92] Lebel, M. et al. Dopamine D1 receptor activation induces tau phosphorylation via cdk5 and GSK3 signaling pathways. *Neuropharmacology* **57**, 392–402 (2009). 19591849 10.1016/j.neuropharm.2009.06.041

[93] Liu, S. L. et al. The role of Cdk5 in Alzheimer’s disease. *Mol. Neurobiol.* **53**, 4328–4342 (2016). 26227906 10.1007/s12035-015-9369-x

[94] Fu, A. K. et al. Cyclin-dependent kinase 5 phosphorylates signal transducer and activator of transcription 3 and regulates its transcriptional activity. *Proc. Natl. Acad. Sci. USA* **101**, 6728–6733 (2004). 15096606 10.1073/pnas.0307606100 PMC404113

[95] Lin, W. et al. Neurotransmitter acetylcholine negatively regulates neuromuscular synapse formation by a Cdk5-dependent mechanism. *Neuron* **46**, 569–579 (2005). 15944126 10.1016/j.neuron.2005.04.002

[96] Wang, C. X. et al. Cyclin-dependent kinase-5 prevents neuronal apoptosis through ERK-mediated upregulation of Bcl-2. *Cell Death Differ.* **13**, 1203–1212 (2006). 16273078 10.1038/sj.cdd.4401804

[97] Malumbres, M. Cyclin-dependent kinases. *Genome Biol.* **15**, 122 (2014). 25180339 10.1186/gb4184 PMC4097832

[98] Chivukula, S. & Malkhed, V. The role of CDK20 protein in carcinogenesis. *Curr. Drug Targets* **24**, 790–796 (2023). 37469151 10.2174/1389450124666230719102112

[99] Kyriakis, J. M. & Avruch, J. Mammalian MAPK signal transduction pathways activated by stress and inflammation: a 10-year update. *Physiol. Rev.* **92**, 689–737 (2012). 22535895 10.1152/physrev.00028.2011

[100] Larhammar, M. et al. The Ste20 family kinases MAP4K4, MINK1, and TNIK converge to regulate stress-induced JNK signaling in neurons. *J. Neurosci.* **37**, 11074–11084 (2017). 28993483 10.1523/JNEUROSCI.0905-17.2017 PMC6596808

[101] Taylor, D. M. et al. MAP kinase phosphatase 1 (MKP-1/DUSP1) is neuroprotective in Huntington’s disease via additive effects of JNK and p38 inhibition. *J. Neurosci.* **33**, 2313–2325 (2013). 23392662 10.1523/JNEUROSCI.4965-11.2013 PMC3711389

[102] Gee, M. S. et al. A selective p38alpha/beta MAPK inhibitor alleviates neuropathology and cognitive impairment, and modulates microglia function in 5XFAD mouse. *Alzheimers Res. Ther.* **12**, 45 (2020). 32317025 10.1186/s13195-020-00617-2 PMC7175487

[103] Shaw, P. C. et al. Isolation and chromosomal mapping of human glycogen synthase kinase-3 alpha and -3 beta encoding genes. *Genome* **41**, 720–727 (1998). 9809441

[104] Yao, H. B., Shaw, P. C., Wong, C. C. & Wan, D. C. Expression of glycogen synthase kinase-3 isoforms in mouse tissues and their transcription in the brain. *J. Chem. Neuroanat.* **23**, 291–297 (2002). 12048112 10.1016/s0891-0618(02)00014-5

[105] Zhao, J. et al. GSK3: a potential target and pending issues for treatment of Alzheimer’s disease. *CNS Neurosci. Ther.* **30**, e14818 (2024). 38946682 10.1111/cns.14818 PMC11215492

[106] Wang, L., Li, J. & Di, L. J. Glycogen synthesis and beyond, a comprehensive review of GSK3 as a key regulator of metabolic pathways and a therapeutic target for treating metabolic diseases. *Med. Res. Rev.* **42**, 946–982 (2022). 34729791 10.1002/med.21867 PMC9298385

[107] Li, S. et al. DYRK1A interacts with histone acetyl transferase p300 and CBP and localizes to enhancers. *Nucleic Acids Res.* **46**, 11202–11213 (2018). 30137413 10.1093/nar/gky754 PMC6265467

[108] Hao, N. et al. Combined computational and experimental analysis reveals mitogen-activated protein kinase-mediated feedback phosphorylation as a mechanism for signaling specificity. *Mol. Biol. Cell* **23**, 3899–3910 (2012). 22875986 10.1091/mbc.E12-04-0333 PMC3459865

[109] Chuang, H. C., Wang, X. & Tan, T. H. MAP4K Family Kinases in Immunity and Inflammation. *Adv. Immunol.* **129**, 277–314 (2016). 26791862 10.1016/bs.ai.2015.09.006

[110] Li, Y. et al. Activation of MAP3K DLK and LZK in Purkinje cells causes rapid and slow degeneration depending on signaling strength. *Elife*. **10**, e63509 (2021). 10.7554/eLife.63509 PMC7870138 33475086

[111] Bos, P. H. et al. Development of MAP4 kinase inhibitors as motor neuron-protecting agents. *Cell Chem. Biol.* **26**, 1703–1715.e1737 (2019). 31676236 10.1016/j.chembiol.2019.10.005 PMC7253076

[112] Arencibia, J. M. et al. AGC protein kinases: from structural mechanism of regulation to allosteric drug development for the treatment of human diseases. *Biochim. Biophys. Acta* **1834**, 1302–1321 (2013). 23524293 10.1016/j.bbapap.2013.03.010

[113] Leroux, A. E., Schulze, J. O. & Biondi, R. M. AGC kinases, mechanisms of regulation and innovative drug development. *Semin Cancer Biol.* **48**, 1–17 (2018). 28591657 10.1016/j.semcancer.2017.05.011

[114] Brandon, E. P., Idzerda, R. L. & McKnight, G. S. PKA isoforms, neural pathways, and behaviour: making the connection. *Curr. Opin. Neurobiol.* **7**, 397–403 (1997). 9232801 10.1016/s0959-4388(97)80069-4

[115] Dagda, R. K. & Das Banerjee, T. Role of protein kinase A in regulating mitochondrial function and neuronal development: implications to neurodegenerative diseases. *Rev. Neurosci.* **26**, 359–370 (2015). 25741943 10.1515/revneuro-2014-0085 PMC4437841

[116] Knighton, D. R. et al. Crystal structure of the catalytic subunit of cyclic adenosine monophosphate-dependent protein kinase. *Science* **253**, 407–414 (1991). 1862342 10.1126/science.1862342

[117] Taylor, S. S. et al. PKA: lessons learned after twenty years. *Biochim Biophys. Acta* **1834**, 1271–1278 (2013). 23535202 10.1016/j.bbapap.2013.03.007 PMC3763834

[118] Zheng, J. et al. Crystal structure of the catalytic subunit of cAMP-dependent protein kinase complexed with MgATP and peptide inhibitor. *Biochemistry* **32**, 2154–2161 (1993). 8443157 10.1021/bi00060a005

[119] Pearce, L. R., Komander, D. & Alessi, D. R. The nuts and bolts of AGC protein kinases. *Nat. Rev. Mol. Cell Biol.* **11**, 9–22 (2010). 20027184 10.1038/nrm2822

[120] Hergovich, A. Regulation and functions of mammalian LATS/NDR kinases: looking beyond canonical Hippo signalling. *Cell Biosci.* **3**, 32 (2013). 23985307 10.1186/2045-3701-3-32 PMC3849777

[121] Turnham, R. E. & Scott, J. D. Protein kinase A catalytic subunit isoform PRKACA; History, function and physiology. *Gene* **577**, 101–108 (2016). 26687711 10.1016/j.gene.2015.11.052 PMC4713328

[122] Pretre, V. & Wicki, A. Inhibition of Akt and other AGC kinases: a target for clinical cancer therapy? *Semin. Cancer Biol.* **48**, 70–77 (2018). 28473255 10.1016/j.semcancer.2017.04.011

[123] Zurashvili, T. et al. Interaction of PDK1 with phosphoinositides is essential for neuronal differentiation but dispensable for neuronal survival. *Mol. Cell Biol.* **33**, 1027–1040 (2013). 23275438 10.1128/MCB.01052-12 PMC3623085

[124] Cordon-Barris, L. et al. Mutation of the 3-phosphoinositide-dependent protein kinase 1 (PDK1) substrate-docking site in the developing brain causes microcephaly with abnormal brain morphogenesis independently of Akt, leading to impaired cognition and disruptive behaviors. *Mol. Cell Biol.* **36**, 2967–2982 (2016). 27644329 10.1128/MCB.00230-16 PMC5108884

[125] Henderson, B. W. et al. Pharmacologic inhibition of LIMK1 provides dendritic spine resilience against beta-amyloid. *Sci Signal*. **12**, eaaw9318 (2019). 10.1126/scisignal.aaw9318 PMC7088434 31239325

[126] Muller, C. P. et al. CaM kinases: from memories to addiction. *Trends Pharm. Sci.* **37**, 153–166 (2016). 26674562 10.1016/j.tips.2015.11.001

[127] Krebs, E. G. & Fischer, E. H. The phosphorylase b to a converting enzyme of rabbit skeletal muscle. *Biochim. Biophys. Acta* **20**, 150–157 (1956). 13315361 10.1016/0006-3002(56)90273-6

[128] Krebs, E. G., Kent, A. B. & Fischer, E. H. The muscle phosphorylase b kinase reaction. *J. Biol. Chem.* **231**, 73–83 (1958). 13538949

[129] Yamauchi, T. & Fujisawa, H. Evidence for three distinct forms of calmodulin-dependent protein kinases from rat brain. *FEBS Lett.* **116**, 141–144 (1980). 7409141 10.1016/0014-5793(80)80628-4

[130] Bayer, K. U. & Schulman, H. CaM Kinase: still Inspiring at 40. *Neuron* **103**, 380–394 (2019). 31394063 10.1016/j.neuron.2019.05.033 PMC6688632

[131] Zhang, L., Luo, B., Lu, Y. & Chen, Y. Targeting death-associated protein kinases for treatment of human diseases: recent advances and future directions. *J. Med. Chem.* **66**, 1112–1136 (2023). 36645394 10.1021/acs.jmedchem.2c01606

[132] Egan, D., Kim, J., Shaw, R. J. & Guan, K. L. The autophagy initiating kinase ULK1 is regulated via opposing phosphorylation by AMPK and mTOR. *Autophagy* **7**, 643–644 (2011). 21460621 10.4161/auto.7.6.15123 PMC3359466

[133] Lin, S. C. & Hardie, D. G. AMPK: sensing glucose as well as cellular energy status. *Cell Metab.* **27**, 299–313 (2018). 29153408 10.1016/j.cmet.2017.10.009

[134] Vingtdeux, V., Davies, P., Dickson, D. W. & Marambaud, P. AMPK is abnormally activated in tangle- and pre-tangle-bearing neurons in Alzheimer’s disease and other tauopathies. *Acta Neuropathol.* **121**, 337–349 (2011). 20957377 10.1007/s00401-010-0759-x PMC3060560

[135] Woods, A. et al. LKB1 is the upstream kinase in the AMP-activated protein kinase cascade. *Curr. Biol.* **13**, 2004–2008 (2003). 14614828 10.1016/j.cub.2003.10.031

[136] Wang, X., Zimmermann, H. R. & Ma, T. Therapeutic potential of AMP-activated protein kinase in Alzheimer’s disease. *J. Alzheimers Dis.* **68**, 33–38 (2019). 30776001 10.3233/JAD-181043 PMC6446925

[137] Martin, S. J., Grimwood, P. D. & Morris, R. G. Synaptic plasticity and memory: an evaluation of the hypothesis. *Annu. Rev. Neurosci.* **23**, 649–711 (2000). 10845078 10.1146/annurev.neuro.23.1.649

[138] Lisman, J., Yasuda, R. & Raghavachari, S. Mechanisms of CaMKII action in long-term potentiation. *Nat. Rev. Neurosci.* **13**, 169–182 (2012). 22334212 10.1038/nrn3192 PMC4050655

[139] Hanson, P. I. & Schulman, H. Inhibitory autophosphorylation of multifunctional Ca2+/calmodulin-dependent protein kinase analyzed by site-directed mutagenesis. *J. Biol. Chem.* **267**, 17216–17224 (1992). 1324926

[140] Lamsa, K., Irvine, E. E., Giese, K. P. & Kullmann, D. M. NMDA receptor-dependent long-term potentiation in mouse hippocampal interneurons shows a unique dependence on Ca(2+)/calmodulin-dependent kinases. *J. Physiol.* **584**, 885–894 (2007). 17884930 10.1113/jphysiol.2007.137380 PMC2276991

[141] Yasuda, R., Hayashi, Y. & Hell, J. W. CaMKII: a central molecular organizer of synaptic plasticity, learning and memory. *Nat. Rev. Neurosci.* **23**, 666–682 (2022). 36056211 10.1038/s41583-022-00624-2

[142] Harda, Z. et al. Autophosphorylation of alphaCaMKII affects social interactions in mice. *Genes Brain Behav.* **17**, e12457 (2018). 29316205 10.1111/gbb.12457

[143] Tao, W. et al. Synaptic memory requires CaMKII. *Elife*. **10**, e60360 (2021). 10.7554/eLife.60360 PMC8798046 34908526

[144] Kim, B. M. et al. Death-associated protein kinase 1 has a critical role in aberrant tau protein regulation and function. *Cell Death Dis.* **5**, e1237 (2014). 24853415 10.1038/cddis.2014.216 PMC4047864

[145] Zhang, T., Kim, B. M. & Lee, T. H. Death-associated protein kinase 1 as a therapeutic target for Alzheimer’s disease. *Transl. Neurodegener.* **13**, 4 (2024). 38195518 10.1186/s40035-023-00395-5 PMC10775678

[146] Su, Y. et al. MicroRNA-26a/death-associated protein kinase 1 signaling induces synucleinopathy and dopaminergic neuron degeneration in Parkinson’s disease. *Biol. Psychiatry* **85**, 769–781 (2019). 30718039 10.1016/j.biopsych.2018.12.008 PMC8861874

[147] Pei, L. et al. DAPK1-p53 interaction converges necrotic and apoptotic pathways of ischemic neuronal death. *J. Neurosci.* **34**, 6546–6556 (2014). 24806680 10.1523/JNEUROSCI.5119-13.2014 PMC6608141

[148] Venerando, A., Ruzzene, M. & Pinna, L. A. Casein kinase: the triple meaning of a misnomer. *Biochem J.* **460**, 141–156 (2014). 24825444 10.1042/BJ20140178

[149] Ikezu, S. & Ikezu, T. Tau-tubulin kinase. *Front Mol. Neurosci.* **7**, 33 (2014). 24808823 10.3389/fnmol.2014.00033 PMC4009424

[150] Fulcher, L. J. & Sapkota, G. P. Functions and regulation of the serine/threonine protein kinase CK1 family: moving beyond promiscuity. *Biochem. J.* **477**, 4603–4621 (2020). 33306089 10.1042/BCJ20200506 PMC7733671

[151] Schittek, B. & Sinnberg, T. Biological functions of casein kinase 1 isoforms and putative roles in tumorigenesis. *Mol. Cancer* **13**, 231 (2014). 25306547 10.1186/1476-4598-13-231 PMC4201705

[152] Knippschild, U. et al. The casein kinase 1 family: participation in multiple cellular processes in eukaryotes. *Cell Signal* **17**, 675–689 (2005). 15722192 10.1016/j.cellsig.2004.12.011

[153] Flajolet, M. et al. Regulation of Alzheimer’s disease amyloid-beta formation by casein kinase I. *Proc. Natl. Acad. Sci. USA* **104**, 4159–4164 (2007). 17360493 10.1073/pnas.0611236104 PMC1820725

[154] Sunkari, Y. K., Meijer, L. & Flajolet, M. The protein kinase CK1: inhibition, activation, and possible allosteric modulation. *Front. Mol. Biosci.* **9**, 916232 (2022). 36090057 10.3389/fmolb.2022.916232 PMC9449355

[155] Gross, S. D. & Anderson, R. A. Casein kinase I: spatial organization and positioning of a multifunctional protein kinase family. *Cell Signal* **10**, 699–711 (1998). 9884021 10.1016/s0898-6568(98)00042-4

[156] Gietzen, K. F. & Virshup, D. M. Identification of inhibitory autophosphorylation sites in casein kinase I epsilon. *J. Biol. Chem.* **274**, 32063–32070 (1999). 10542239 10.1074/jbc.274.45.32063

[157] Singh, T. J., Grundke-Iqbal, I. & Iqbal, K. Phosphorylation of tau protein by casein kinase-1 converts it to an abnormal Alzheimer-like state. *J. Neurochem.* **64**, 1420–1423 (1995). 7532213 10.1046/j.1471-4159.1995.64031420.x

[158] Desdouits, F., Siciliano, J. C., Greengard, P. & Girault, J. A. Dopamine- and cAMP-regulated phosphoprotein DARPP-32: phosphorylation of Ser-137 by casein kinase I inhibits dephosphorylation of Thr-34 by calcineurin. *Proc. Natl. Acad. Sci. USA* **92**, 2682–2685 (1995). 7708705 10.1073/pnas.92.7.2682 PMC42282

[159] Kloss, B., Rothenfluh, A., Young, M. W. & Saez, L. Phosphorylation of period is influenced by cycling physical associations of double-time, period, and timeless in the Drosophila clock. *Neuron* **30**, 699–706 (2001). 11430804 10.1016/s0896-6273(01)00320-8

[160] Marin, O. et al. A noncanonical sequence phosphorylated by casein kinase 1 in beta-catenin may play a role in casein kinase 1 targeting of important signaling proteins. *Proc. Natl. Acad. Sci. USA* **100**, 10193–10200 (2003). 12925738 10.1073/pnas.1733909100 PMC193538

[161] Liu, C. et al. Control of beta-catenin phosphorylation/degradation by a dual-kinase mechanism. *Cell* **108**, 837–847 (2002). 11955436 10.1016/s0092-8674(02)00685-2

[162] Amit, S. et al. Axin-mediated CKI phosphorylation of beta-catenin at Ser 45: a molecular switch for the Wnt pathway. *Genes Dev.* **16**, 1066–1076 (2002). 12000790 10.1101/gad.230302 PMC186245

[163] Winter, M. et al. Protein kinase CK1delta phosphorylates key sites in the acidic domain of murine double-minute clone 2 protein (MDM2) that regulate p53 turnover. *Biochemistry* **43**, 16356–16364 (2004). 15610030 10.1021/bi0489255

[164] Xu, F. et al. Mammalian sterile 20-like kinase 1/2 inhibits the Wnt/beta-catenin signalling pathway by directly binding casein kinase 1epsilon. *Biochem. J.* **458**, 159–169 (2014). 24180524 10.1042/BJ20130986

[165] Liu, C. Y. et al. The hippo tumor pathway promotes TAZ degradation by phosphorylating a phosphodegron and recruiting the SCF{beta}-TrCP E3 ligase. *J. Biol. Chem.* **285**, 37159–37169 (2010). 20858893 10.1074/jbc.M110.152942 PMC2988322

[166] Denef, N., Neubuser, D., Perez, L. & Cohen, S. M. Hedgehog induces opposite changes in turnover and subcellular localization of patched and smoothened. *Cell* **102**, 521–531 (2000). 10966113 10.1016/s0092-8674(00)00056-8

[167] Chen, Y. et al. Sonic Hedgehog-dependent phosphorylation by CK1alpha and GRK2 is required for ciliary accumulation and activation of smoothened. *PLoS Biol.* **9**, e1001083 (2011). 21695114 10.1371/journal.pbio.1001083 PMC3114773

[168] De Wit, T., Baekelandt, V. & Lobbestael, E. Inhibition of LRRK2 or casein kinase 1 results in LRRK2 protein destabilization. *Mol. Neurobiol.* **56**, 5273–5286 (2019). 30592011 10.1007/s12035-018-1449-2 PMC6657425

[169] Sergeant, N. et al. Biochemistry of Tau in Alzheimer’s disease and related neurological disorders. *Expert Rev. Proteom.* **5**, 207–224 (2008). 10.1586/14789450.5.2.207 18466052

[170] Sato, S., Cerny, R. L., Buescher, J. L. & Ikezu, T. Tau-tubulin kinase 1 (TTBK1), a neuron-specific tau kinase candidate, is involved in tau phosphorylation and aggregation. *J. Neurochem.* **98**, 1573–1584 (2006). 16923168 10.1111/j.1471-4159.2006.04059.x

[171] Fry, A. M., O’Regan, L., Sabir, S. R. & Bayliss, R. Cell cycle regulation by the NEK family of protein kinases. *J. Cell Sci.* **125**, 4423–4433 (2012). 23132929 10.1242/jcs.111195 PMC3500863

[172] Johnson, L. N., Noble, M. E. & Owen, D. J. Active and inactive protein kinases: structural basis for regulation. *Cell* **85**, 149–158 (1996). 8612268 10.1016/s0092-8674(00)81092-2

[173] Hinz, M. & Scheidereit, C. The IkappaB kinase complex in NF-kappaB regulation and beyond. *EMBO Rep.* **15**, 46–61 (2014). 24375677 10.1002/embr.201337983 PMC4303448

[174] Courtois, G. & Gilmore, T. D. Mutations in the NF-kappaB signaling pathway: implications for human disease. *Oncogene* **25**, 6831–6843 (2006). 17072331 10.1038/sj.onc.1209939

[175] Xiang, S. et al. TANK-binding kinase 1 (TBK1): An emerging therapeutic target for drug discovery. *Drug Discov. Today* **26**, 2445–2455 (2021). 34051368 10.1016/j.drudis.2021.05.016

[176] Tu, D. et al. Structure and ubiquitination-dependent activation of TANK-binding kinase 1. *Cell Rep.* **3**, 747–758 (2013). 23453972 10.1016/j.celrep.2013.01.033 PMC3863638

[177] Gerbino, V. et al. The loss of TBK1 kinase activity in motor neurons or in all cell types differentially impacts ALS disease progression in SOD1 mice. *Neuron* **106**, 789–805.e785 (2020). 32220666 10.1016/j.neuron.2020.03.005

[178] Sharma, S. et al. Triggering the interferon antiviral response through an IKK-related pathway. *Science* **300**, 1148–1151 (2003). 12702806 10.1126/science.1081315

[179] Kim, J. Y. et al. Dissection of TBK1 signaling via phosphoproteomics in lung cancer cells. *Proc. Natl. Acad. Sci. USA* **110**, 12414–12419 (2013). 23836654 10.1073/pnas.1220674110 PMC3725062

[180] Tokumitsu, H. & Sakagami, H. Molecular mechanisms underlying Ca(2+)/calmodulin-dependent protein kinase kinase signal transduction. *Int. J. Mol. Sci*. **23**, 11025 (2022). 10.3390/ijms231911025 PMC9570080 36232320

[181] Guo, T. et al. Molecular and cellular mechanisms underlying the pathogenesis of Alzheimer’s disease. *Mol. Neurodegener.* **15**, 40 (2020). 32677986 10.1186/s13024-020-00391-7 PMC7364557

[182] Tarawneh, R. & Holtzman, D. M. The clinical problem of symptomatic Alzheimer disease and mild cognitive impairment. *CSH Perspect. Med.* **2**, a006148 (2012). 10.1101/cshperspect.a006148 PMC3331682 22553492

[183] Walczak-Nowicka, L. J. & Herbet, M. Acetylcholinesterase inhibitors in the treatment of neurodegenerative diseases and the role of acetylcholinesterase in their pathogenesis. *Int. J. Mol. Sci*. **22**, 9290 (2021). 10.3390/ijms22179290 PMC8430571 34502198

[184] Song, X. et al. Mechanism of NMDA receptor channel block by MK-801 and memantine. *Nature* **556**, 515–519 (2018). 29670280 10.1038/s41586-018-0039-9 PMC5962351

[185] Tolar, M. et al. Aducanumab, gantenerumab, BAN2401, and ALZ-801-the first wave of amyloid-targeting drugs for Alzheimer’s disease with potential for near-term approval. *Alzheimers Res Ther.* **12**, 95 (2020). 32787971 10.1186/s13195-020-00663-w PMC7424995

[186] Mintun, M. A. et al. Donanemab in early Alzheimer’s Disease. *N. Engl. J. Med.* **384**, 1691–1704 (2021). 33720637 10.1056/NEJMoa2100708

[187] Tonnies, E. & Trushina, E. Oxidative stress, synaptic dysfunction, and Alzheimer’s Disease. *J. Alzheimers Dis.* **57**, 1105–1121 (2017). 28059794 10.3233/JAD-161088 PMC5409043

[188] Iqbal, K., Liu, F. & Gong, C. X. Tau and neurodegenerative disease: the story so far. *Nat. Rev. Neurol.* **12**, 15–27 (2016). 26635213 10.1038/nrneurol.2015.225

[189] Greenberg, S. M. et al. Cerebral amyloid angiopathy and Alzheimer disease—one peptide, two pathways. *Nat. Rev. Neurol.* **16**, 30–42 (2020). 31827267 10.1038/s41582-019-0281-2 PMC7268202

[190] Sharoar, M. G. et al. Accumulation of saposin in dystrophic neurites is linked to impaired lysosomal functions in Alzheimer’s disease brains. *Mol. Neurodegener.* **16**, 45 (2021). 34215298 10.1186/s13024-021-00464-1 PMC8254260

[191] Lei, P. et al. Tau deficiency induces parkinsonism with dementia by impairing APP-mediated iron export. *Nat. Med.* **18**, 291–295 (2012). 22286308 10.1038/nm.2613

[192] Jiao, L. et al. Iron metabolism mediates microglia susceptibility in ferroptosis. *Front. Cell Neurosci.* **16**, 995084 (2022). 36111246 10.3389/fncel.2022.995084 PMC9469838

[193] Kwapong, W. R. et al. Choriocapillaris reduction accurately discriminates against early-onset Alzheimer’s disease. *Alzheimers Dement.* **20**, 4185–4198 (2024). 38747519 10.1002/alz.13871 PMC11180859

[194] Yan, H. F., Tuo, Q. Z. & Lei, P. Cell density impacts the susceptibility to ferroptosis by modulating IRP1-mediated iron homeostasis. *J. Neurochem.* **168**, 1359–1373 (2024). 38382918 10.1111/jnc.16085

[195] Zhang, Y. W., Thompson, R., Zhang, H. & Xu, H. APP processing in Alzheimer’s disease. *Mol. Brain* **4**, 3 (2011). 21214928 10.1186/1756-6606-4-3 PMC3022812

[196] Iwatsubo, T. et al. Visualization of A beta 42(43) and A beta 40 in senile plaques with end-specific A beta monoclonals: evidence that an initially deposited species is A beta 42(43). *Neuron* **13**, 45–53 (1994). 8043280 10.1016/0896-6273(94)90458-8

[197] Lei, P., Ayton, S. & Bush, A. I. The essential elements of Alzheimer’s disease. *J. Biol. Chem.* **296**, 100105 (2021). 33219130 10.1074/jbc.REV120.008207 PMC7948403

[198] Congdon, E. E. & Sigurdsson, E. M. Tau-targeting therapies for Alzheimer disease. *Nat. Rev. Neurol.* **14**, 399–415 (2018). 29895964 10.1038/s41582-018-0013-z PMC6463489

[199] Lei, P. & Ayton, S. TRIMming the tangles. *Sci. Bull.* **68**, 2507–2509 (2023). 10.1016/j.scib.2023.09.019 37758617

[200] Lei, P. et al. Motor and cognitive deficits in aged tau knockout mice in two background strains. *Mol. Neurodegener.* **9**, 29 (2014). 25124182 10.1186/1750-1326-9-29 PMC4141346

[201] Augustinack, J. C., Schneider, A., Mandelkow, E. M. & Hyman, B. T. Specific tau phosphorylation sites correlate with severity of neuronal cytopathology in Alzheimer’s disease. *Acta Neuropathol.* **103**, 26–35 (2002). 11837744 10.1007/s004010100423

[202] Shukla, V., Skuntz, S. & Pant, H. C. Deregulated Cdk5 activity is involved in inducing Alzheimer’s disease. *Arch. Med. Res.* **43**, 655–662 (2012). 23142263 10.1016/j.arcmed.2012.10.015 PMC3532552

[203] Planel, E., Sun, X. & Takashima, A. Role of GSK-3? In Alzheimer’s disease pathology. *Drug Dev. Res.* **56**, 491–510 (2002).

[204] Zhu, X. et al. Activation and redistribution of c-jun N-terminal kinase/stress-activated protein kinase in degenerating neurons in Alzheimer’s disease. *J. Neurochem.* **76**, 435–441 (2001). 11208906 10.1046/j.1471-4159.2001.00046.x

[205] Platenik, J. et al. GSK3beta, CREB, and BDNF in peripheral blood of patients with Alzheimer’s disease and depression. *Prog. Neuropsychopharmacol. Biol. Psychiatry* **50**, 83–93 (2014). 24334212 10.1016/j.pnpbp.2013.12.001

[206] Triaca, V. et al. NGF controls APP cleavage by downregulating APP phosphorylation at Thr668: relevance for Alzheimer’s disease. *Aging Cell* **15**, 661–672 (2016). 27076121 10.1111/acel.12473 PMC4933663

[207] Rockenstein, E. et al. Neuroprotective effects of regulators of the glycogen synthase kinase-3beta signaling pathway in a transgenic model of Alzheimer’s disease are associated with reduced amyloid precursor protein phosphorylation. *J. Neurosci.* **27**, 1981–1991 (2007). 17314294 10.1523/JNEUROSCI.4321-06.2007 PMC6673566

[208] Lauretti, E., Dincer, O. & Pratico, D. Glycogen synthase kinase-3 signaling in Alzheimer’s disease. *Biochim Biophys. Acta Mol. Cell Res.* **1867**, 118664 (2020). 32006534 10.1016/j.bbamcr.2020.118664 PMC7047718

[209] Qian, W. et al. PP2A regulates tau phosphorylation directly and also indirectly via activating GSK-3beta. *J. Alzheimers Dis.* **19**, 1221–1229 (2010). 20308788 10.3233/JAD-2010-1317

[210] Manoharan, S. D. et al. Could protein phosphatase 2A and glycogen synthase kinase-3 beta be targeted by natural compounds to ameliorate Alzheimer’s pathologies? *Brain Res.* **1829**, 148793 (2024). 38309553 10.1016/j.brainres.2024.148793

[211] Baki, L. et al. PS1 activates PI3K thus inhibiting GSK-3 activity and tau overphosphorylation: effects of FAD mutations. *EMBO J.* **23**, 2586–2596 (2004). 15192701 10.1038/sj.emboj.7600251 PMC449766

[212] Hadi, F., Akrami, H., Shahpasand, K. & Fattahi, M. R. Wnt signalling pathway and tau phosphorylation: a comprehensive study on known connections. *Cell Biochem. Funct.* **38**, 686–694 (2020). 32232872 10.1002/cbf.3530

[213] Zhang, Z. et al. Destabilization of beta-catenin by mutations in presenilin-1 potentiates neuronal apoptosis. *Nature* **395**, 698–702 (1998). 9790190 10.1038/27208

[214] Mishra, R. et al. Glycogen synthase kinase-3beta induces neuronal cell death via direct phosphorylation of mixed lineage kinase 3. *J. Biol. Chem.* **282**, 30393–30405 (2007). 17711861 10.1074/jbc.M705895200 PMC5323256

[215] Liu, X. H. et al. Blocking GSK3beta-mediated dynamin1 phosphorylation enhances BDNF-dependent TrkB endocytosis and the protective effects of BDNF in neuronal and mouse models of Alzheimer’s disease. *Neurobiol. Dis.* **74**, 377–391 (2015). 25484286 10.1016/j.nbd.2014.11.020

[216] Mercado-Gomez, O. et al. Inhibition of Wnt and PI3K signaling modulates GSK-3beta activity and induces morphological changes in cortical neurons: role of tau phosphorylation. *Neurochem. Res.* **33**, 1599–1609 (2008). 18461448 10.1007/s11064-008-9714-9

[217] Fao, L., Mota, S. I. & Rego, A. C. Shaping the Nrf2-ARE-related pathways in Alzheimer’s and Parkinson’s diseases. *Ageing Res. Rev.* **54**, 100942 (2019). 31415806 10.1016/j.arr.2019.100942

[218] Ko, C. Y. et al. Glycogen synthase kinase-3beta-mediated CCAAT/enhancer-binding protein delta phosphorylation in astrocytes promotes migration and activation of microglia/macrophages. *Neurobiol. Aging* **35**, 24–34 (2014). 23993701 10.1016/j.neurobiolaging.2013.07.021

[219] Hernandez, F., Lucas, J. J. & Avila, J. GSK3 and tau: two convergence points in Alzheimer’s disease. *J. Alzheimers Dis.* **33**, S141–S144 (2013). 22710914 10.3233/JAD-2012-129025

[220] Ly, P. T. et al. Inhibition of GSK3 beta-mediated BACE1 expression reduces Alzheimer-associated phenotypes. *J. Clin. Investig.* **123**, 224–235 (2013). 23202730 10.1172/JCI64516 PMC3533290

[221] Zempel, H., Thies, E., Mandelkow, E. & Mandelkow, E. M. Abeta oligomers cause localized Ca(2+) elevation, missorting of endogenous Tau into dendrites, Tau phosphorylation, and destruction of microtubules and spines. *J. Neurosci.* **30**, 11938–11950 (2010). 20826658 10.1523/JNEUROSCI.2357-10.2010 PMC6633549

[222] Cruz, J. C. et al. Aberrant Cdk5 activation by p25 triggers pathological events leading to neurodegeneration and neurofibrillary tangles. *Neuron* **40**, 471–483 (2003). 14642273 10.1016/s0896-6273(03)00627-5

[223] Lopes, J. P., Oliveira, C. R. & Agostinho, P. Neurodegeneration in an Abeta-induced model of Alzheimer’s disease: the role of Cdk5. *Aging Cell* **9**, 64–77 (2010). 19895631 10.1111/j.1474-9726.2009.00536.x

[224] Matrone, C. et al. Tyrosine kinase nerve growth factor receptor switches from prosurvival to proapoptotic activity via Abeta-mediated phosphorylation. *Proc. Natl. Acad. Sci. USA* **106**, 11358–11363 (2009). 19549834 10.1073/pnas.0904998106 PMC2699376

[225] Lau, K. F. et al. Cyclin-dependent kinase-5/p35 phosphorylates Presenilin 1 to regulate carboxy-terminal fragment stability. *Mol. Cell Neurosci.* **20**, 13–20 (2002). 12056836 10.1006/mcne.2002.1108

[226] Jayapalan, S. & Natarajan, J. The role of CDK5 and GSK3B kinases in hyperphosphorylation of microtubule-associated protein tau (MAPT) in Alzheimer’s disease. *Bioinformation* **9**, 1023–1030 (2013). 24497730 10.6026/97320630091023 PMC3910359

[227] Cancino, G. I. et al. c-Abl tyrosine kinase modulates tau pathology and Cdk5 phosphorylation in AD transgenic mice. *Neurobiol. Aging* **32**, 1249–1261 (2011). 19700222 10.1016/j.neurobiolaging.2009.07.007

[228] Tian, B., Yang, Q. & Mao, Z. Phosphorylation of ATM by Cdk5 mediates DNA damage signalling and regulates neuronal death. *Nat. Cell Biol.* **11**, 211–218 (2009). 19151707 10.1038/ncb1829 PMC2760486

[229] Sun, K. H., Lee, H. G., Smith, M. A. & Shah, K. Direct and indirect roles of cyclin-dependent kinase 5 as an upstream regulator in the c-Jun NH2-terminal kinase cascade: relevance to neurotoxic insults in Alzheimer’s disease. *Mol. Biol. Cell* **20**, 4611–4619 (2009). 19776350 10.1091/mbc.E09-05-0433 PMC2770948

[230] Li, B. S. et al. Cyclin-dependent kinase 5 prevents neuronal apoptosis by negative regulation of c-Jun N-terminal kinase 3. *EMBO J.* **21**, 324–333 (2002). 11823425 10.1093/emboj/21.3.324 PMC125822

[231] Gong, X. et al. Cdk5-mediated inhibition of the protective effects of transcription factor MEF2 in neurotoxicity-induced apoptosis. *Neuron* **38**, 33–46 (2003). 12691662 10.1016/s0896-6273(03)00191-0

[232] Sun, K. H., de Pablo, Y., Vincent, F. & Shah, K. Deregulated Cdk5 promotes oxidative stress and mitochondrial dysfunction. *J. Neurochem.* **107**, 265–278 (2008). 18691386 10.1111/j.1471-4159.2008.05616.x

[233] Park, J. et al. Loss of mitofusin 2 links beta-amyloid-mediated mitochondrial fragmentation and Cdk5-induced oxidative stress in neuron cells. *J. Neurochem.* **132**, 687–702 (2015). 25359615 10.1111/jnc.12984

[234] Chang, K. H., Vincent, F. & Shah, K. Deregulated Cdk5 triggers aberrant activation of cell cycle kinases and phosphatases inducing neuronal death. *J. Cell Sci.* **125**, 5124–5137 (2012). 22899714 10.1242/jcs.108183

[235] Kim, D. et al. Deregulation of HDAC1 by p25/Cdk5 in neurotoxicity. *Neuron* **60**, 803–817 (2008). 19081376 10.1016/j.neuron.2008.10.015 PMC2912147

[236] Chow, H. M. et al. Age-related hyperinsulinemia leads to insulin resistance in neurons and cell-cycle-induced senescence. *Nat. Neurosci.* **22**, 1806–1819 (2019). 31636448 10.1038/s41593-019-0505-1

[237] Xie, W. et al. CEND1 deficiency induces mitochondrial dysfunction and cognitive impairment in Alzheimer’s disease. *Cell Death Differ.* **29**, 2417–2428 (2022). 35732922 10.1038/s41418-022-01027-7 PMC9751129

[238] Zhou, J. et al. Cyclin-Dependent Kinase 5-Dependent BAG3 Degradation Modulates Synaptic Protein Turnover. *Biol. Psychiatry* **87**, 756–769 (2020). 31955914 10.1016/j.biopsych.2019.11.013

[239] Li, Z. et al. Astrocytes deliver CK1 to neurons via extracellular vesicles in response to inflammation promoting the translation and amyloidogenic processing of APP. *J. Extracell. Vesicles* **10**, e12035 (2020). 33408815 10.1002/jev2.12035 PMC7775567

[240] Roth, A. et al. CK1delta-derived peptides as novel tools inhibiting the interactions between CK1delta and APP695 to modulate the pathogenic metabolism of APP. *Int. J .Mol. Sci*. **22**, 6423 (2021). 10.3390/ijms22126423 PMC8232658 34203978

[241] Kuret, J. et al. Casein kinase 1 is tightly associated with paired-helical filaments isolated from Alzheimer’s disease brain. *J. Neurochem.* **69**, 2506–2515 (1997). 9375684 10.1046/j.1471-4159.1997.69062506.x

[242] Yang, M. et al. Casein Kinase 1delta phosphorylates TDP-43 and suppresses its function in tau mRNA processing. *J. Alzheimers Dis.* **91**, 1527–1539 (2023). 36641675 10.3233/JAD-220985

[243] Roth, A. et al. Comprehensive characterization of CK1delta-mediated tau phosphorylation in Alzheimer’s Disease. *Front. Mol. Biosci.* **9**, 872171 (2022). 36203870 10.3389/fmolb.2022.872171 PMC9531328

[244] Meijer, L., Flajolet, M. & Greengard, P. Pharmacological inhibitors of glycogen synthase kinase 3. *Trends Pharm. Sci.* **25**, 471–480 (2004). 15559249 10.1016/j.tips.2004.07.006

[245] Liu, F. et al. Regulation of cyclin-dependent kinase 5 and casein kinase 1 by metabotropic glutamate receptors. *Proc. Natl. Acad. Sci. USA* **98**, 11062–11068 (2001). 11572969 10.1073/pnas.191353898 PMC58683

[246] Walter, J. et al. Phosphorylation regulates intracellular trafficking of beta-secretase. *J. Biol. Chem.* **276**, 14634–14641 (2001). 11278841 10.1074/jbc.M011116200

[247] Tong, L., Thornton, P. L., Balazs, R. & Cotman, C. W. Beta -amyloid-(1-42) impairs activity-dependent cAMP-response element-binding protein signaling in neurons at concentrations in which cell survival Is not compromised. *J. Biol. Chem.* **276**, 17301–17306 (2001). 11278679 10.1074/jbc.M010450200

[248] Meng, C., He, Z. & Xing, D. Low-level laser therapy rescues dendrite atrophy via upregulating BDNF expression: implications for Alzheimer’s disease. *J. Neurosci.* **33**, 13505–13517 (2013). 23946409 10.1523/JNEUROSCI.0918-13.2013 PMC6705158

[249] Liang, Z. et al. Down-regulation of cAMP-dependent protein kinase by over-activated calpain in Alzheimer’s disease brain. *J. Neurochem.* **103**, 2462–2470 (2007). 17908236 10.1111/j.1471-4159.2007.04942.x PMC2262109

[250] Tong, L., Balazs, R., Thornton, P. L. & Cotman, C. W. Beta-amyloid peptide at sublethal concentrations downregulates brain-derived neurotrophic factor functions in cultured cortical neurons. *J. Neurosci.* **24**, 6799–6809 (2004). 15282285 10.1523/JNEUROSCI.5463-03.2004 PMC6729714

[251] Chen, Y. et al. Alzheimer’s beta-secretase (BACE1) regulates the cAMP/PKA/CREB pathway independently of beta-amyloid. *J. Neurosci.* **32**, 11390–11395 (2012). 22895721 10.1523/JNEUROSCI.0757-12.2012 PMC3446780

[252] Herskovits, A. Z. & Guarente, L. SIRT1 in neurodevelopment and brain senescence. *Neuron* **81**, 471–483 (2014). 24507186 10.1016/j.neuron.2014.01.028 PMC4040287

[253] Zhang, Z. et al. Activation of PKA/SIRT1 signaling pathway by photobiomodulation therapy reduces Abeta levels in Alzheimer’s disease models. *Aging Cell* **19**, e13054 (2020). 31663252 10.1111/acel.13054 PMC6974721

[254] Munoz, L. & Ammit, A. J. Targeting p38 MAPK pathway for the treatment of Alzheimer’s disease. *Neuropharmacology* **58**, 561–568 (2010). 19951717 10.1016/j.neuropharm.2009.11.010

[255] Lee, J. C. et al. A protein kinase involved in the regulation of inflammatory cytokine biosynthesis. *Nature* **372**, 739–746 (1994). 7997261 10.1038/372739a0

[256] Sun, A. P38 MAP kinase is activated at early stages in Alzheimer’s disease brain. *Exp. Neurol.* **183**, 394–405 (2003). 14552880 10.1016/s0014-4886(03)00180-8

[257] Kim, S. H., Smith, C. J. & Van Eldik, L. J. Importance of MAPK pathways for microglial pro-inflammatory cytokine IL-1 beta production. *Neurobiol. Aging* **25**, 431–439 (2004). 15013563 10.1016/S0197-4580(03)00126-X

[258] Decourt, B., Lahiri, D. K. & Sabbagh, M. N. Targeting tumor necrosis factor-alpha for Alzheimer’s Disease. *Curr. Alzheimer Res.* **14**, 412–425 (2017). 27697064 10.2174/1567205013666160930110551 PMC5328927

[259] Lee, Y. B., Schrader, J. W. & Kim, S. U. p38 map kinase regulates TNF-alpha production in human astrocytes and microglia by multiple mechanisms. *Cytokine* **12**, 874–880 (2000). 10880231 10.1006/cyto.2000.0688

[260] Hua, L. L. et al. Role of mitogen-activated protein kinases in inducible nitric oxide synthase and TNFalpha expression in human fetal astrocytes. *J. Neuroimmunol.* **126**, 180–189 (2002). 12020969 10.1016/s0165-5728(02)00055-3

[261] Keum, Y. S. et al. Mechanism of action of sulforaphane: inhibition of p38 mitogen-activated protein kinase isoforms contributing to the induction of antioxidant response element-mediated heme oxygenase-1 in human hepatoma HepG2 cells. *Cancer Res.* **66**, 8804–8813 (2006). 16951197 10.1158/0008-5472.CAN-05-3513

[262] Kelleher, I. et al. Kinase activities increase during the development of tauopathy in htau mice. *J. Neurochem.* **103**, 2256–2267 (2007). 17908241 10.1111/j.1471-4159.2007.04930.x

[263] Griffin, R. et al. The age-related attenuation in long-term potentiation is associated with microglial activation. *J. Neurochem.* **99**, 1263–1272 (2006). 16981890 10.1111/j.1471-4159.2006.04165.x

[264] Bains, J. S. & Oliet, S. H. Glia: they make your memories stick! *Trends Neurosci.* **30**, 417–424 (2007). 17631972 10.1016/j.tins.2007.06.007

[265] Luo, Q. et al. p38alpha-MAPK-deficient myeloid cells ameliorate symptoms and pathology of APP-transgenic Alzheimer’s disease mice. *Aging Cell* **21**, e13679 (2022). 35909315 10.1111/acel.13679 PMC9381888

[266] Schnoder, L. et al. Neuronal deficiency of p38alpha-MAPK ameliorates symptoms and pathology of APP or Tau-transgenic Alzheimer’s mouse models. *FASEB J.* **34**, 9628–9649 (2020). 32475008 10.1096/fj.201902731RR

[267] Schnoder, L. et al. Deficiency of neuronal p38alpha MAPK attenuates amyloid pathology in Alzheimer disease mouse and cell models through facilitating lysosomal degradation of BACE1. *J. Biol. Chem.* **291**, 2067–2079 (2016). 26663083 10.1074/jbc.M115.695916 PMC4732195

[268] Ittner, A. et al. Site-specific phosphorylation of tau inhibits amyloid-beta toxicity in Alzheimer’s mice. *Science* **354**, 904–908 (2016). 27856911 10.1126/science.aah6205

[269] Ittner, A. & Ittner, L. M. Dendritic tau in Alzheimer’s Disease. *Neuron* **99**, 13–27 (2018). 30001506 10.1016/j.neuron.2018.06.003

[270] Ittner, A. et al. Reduction of advanced tau-mediated memory deficits by the MAP kinase p38gamma. *Acta Neuropathol.* **140**, 279–294 (2020). 32725265 10.1007/s00401-020-02191-1

[271] Ittner, L. M. et al. Dendritic function of tau mediates amyloid-beta toxicity in Alzheimer’s disease mouse models. *Cell* **142**, 387–397 (2010). 20655099 10.1016/j.cell.2010.06.036

[272] Matrone, C., Iannuzzi, F. & Annunziato, L. The Y682ENPTY687 motif of APP: Progress and insights toward a targeted therapy for Alzheimer’s disease patients. *Ageing Res. Rev.* **52**, 120–128 (2019). 31039414 10.1016/j.arr.2019.04.003

[273] Um, J. W. et al. Alzheimer’s amyloid-beta oligomer bound to postsynaptic prion protein activates Fyn to impair neurons. *Nat. Neurosci.* **15**, 1227–1235 (2012). 22820466 10.1038/nn.3178 PMC3431439

[274] Nygaard, H. B. Targeting Fyn kinase in Alzheimer’s Disease. *Biol. Psychiatry* **83**, 369–376 (2018). 28709498 10.1016/j.biopsych.2017.06.004 PMC5729051

[275] Hernandez, P., Lee, G., Sjoberg, M. & Maccioni, R. B. Tau phosphorylation by cdk5 and Fyn in response to amyloid peptide Abeta (25–35): involvement of lipid rafts. *J. Alzheimers Dis.* **16**, 149–156 (2009). 19158430 10.3233/JAD-2009-0933

[276] Lee, G. et al. Phosphorylation of tau by fyn: implications for Alzheimer’s disease. *J. Neurosci.* **24**, 2304–2312 (2004). 14999081 10.1523/JNEUROSCI.4162-03.2004 PMC6730442

[277] Roberson, E. D. et al. Amyloid-beta/Fyn-induced synaptic, network, and cognitive impairments depend on tau levels in multiple mouse models of Alzheimer’s disease. *J. Neurosci.* **31**, 700–711 (2011). 21228179 10.1523/JNEUROSCI.4152-10.2011 PMC3325794

[278] Lau, D. H. et al. Critical residues involved in tau binding to fyn: implications for tau phosphorylation in Alzheimer’s disease. *Acta Neuropathol. Commun.* **4**, 49 (2016). 27193083 10.1186/s40478-016-0317-4 PMC4870772

[279] Lopes, S. et al. Tau protein is essential for stress-induced brain pathology. *Proc. Natl. Acad. Sci. USA* **113**, E3755–E3763 (2016). 27274066 10.1073/pnas.1600953113 PMC4932951

[280] Rong, Y. et al. Tyrosine phosphorylation of ionotropic glutamate receptors by Fyn or Src differentially modulates their susceptibility to calpain and enhances their binding to spectrin and PSD-95. *J. Neurochem.* **79**, 382–390 (2001). 11677266 10.1046/j.1471-4159.2001.00565.x

[281] Kaufman, A. C. et al. Fyn inhibition rescues established memory and synapse loss in Alzheimer’s mice. *Ann. Neurol.* **77**, 953–971 (2015). 25707991 10.1002/ana.24394 PMC4447598

[282] Li, C. & Gotz, J. Somatodendritic accumulation of Tau in Alzheimer’s disease is promoted by Fyn-mediated local protein translation. *EMBO J.* **36**, 3120–3138 (2017). 28864542 10.15252/embj.201797724 PMC5666608

[283] Sato, S. et al. Spatial learning impairment, enhanced CDK5/p35 activity, and downregulation of NMDA receptor expression in transgenic mice expressing tau-tubulin kinase 1. *J. Neurosci.* **28**, 14511–14521 (2008). 19118186 10.1523/JNEUROSCI.3417-08.2008 PMC6671237

[284] Vazquez-Higuera, J. L. et al. Genetic variations in tau-tubulin kinase-1 are linked to Alzheimer’s disease in a Spanish case-control cohort. *Neurobiol. Aging* **32**, 550.e555–559 (2011). 10.1016/j.neurobiolaging.2009.12.021 20096481

[285] Yu, N. N. et al. Tau-tubulin kinase-1 gene variants are associated with Alzheimer’s disease in Han Chinese. *Neurosci. Lett.* **491**, 83–86 (2011). 21219968 10.1016/j.neulet.2011.01.011

[286] Lund, H. et al. Tau-tubulin kinase 1 expression, phosphorylation and co-localization with phospho-Ser422 tau in the Alzheimer’s disease brain. *Brain Pathol.* **23**, 378–389 (2013). 23088643 10.1111/bpa.12001 PMC8029021

[287] Halkina, T. et al. Discovery of potent and brain-penetrant tau tubulin kinase 1 (TTBK1) inhibitors that lower tau phosphorylation in vivo. *J. Med. Chem.* **64**, 6358–6380 (2021). 33944571 10.1021/acs.jmedchem.1c00382

[288] Simpkins, K. L. et al. Selective activation induced cleavage of the NR2B subunit by calpain. *J. Neurosci.* **23**, 11322–11331 (2003). 14672996 10.1523/JNEUROSCI.23-36-11322.2003 PMC6740527

[289] Zhang, S. et al. Cdk5 regulates the phosphorylation of tyrosine 1472 NR2B and the surface expression of NMDA receptors. *J. Neurosci.* **28**, 415–424 (2008). 18184784 10.1523/JNEUROSCI.1900-07.2008 PMC6670547

[290] Hawasli, A. H. et al. Cyclin-dependent kinase 5 governs learning and synaptic plasticity via control of NMDAR degradation. *Nat. Neurosci.* **10**, 880–886 (2007). 17529984 10.1038/nn1914 PMC3910113

[291] Wang, Q. et al. Scopolamine causes delirium-like brain network dysfunction and reversible cognitive impairment without neuronal loss. *Zool. Res.* **44**, 712–724 (2023). 37313848 10.24272/j.issn.2095-8137.2022.473 PMC10415773

[292] Assefa, B. T., Tafere, G. G., Wondafrash, D. Z. & Gidey, M. T. The bewildering effect of AMPK activators in Alzheimer’s Disease: review of the current evidence. *Biomed. Res. Int.* **2020**, 9895121 (2020). 32149150 10.1155/2020/9895121 PMC7049408

[293] Ibrahim, W. W., Kamel, A. S., Wahid, A. & Abdelkader, N. F. Dapagliflozin as an autophagic enhancer via LKB1/AMPK/SIRT1 pathway in ovariectomized/D-galactose Alzheimer’s rat model. *Inflammopharmacology* **30**, 2505–2520 (2022). 10.1007/s10787-022-00973-5 PMC9700568 35364737

[294] Schubert, D. Glucose metabolism and Alzheimer’s disease. *Ageing Res. Rev.* **4**, 240–257 (2005). 15950548 10.1016/j.arr.2005.02.003

[295] Brown, G. C., Murphy, M. P., Jornayvaz, F. R. & Shulman, G. I. Regulation of mitochondrial biogenesis. *Essays Biochem.* **47**, 69–84 (2010). 20533901 10.1042/bse0470069 PMC3883043

[296] Ling, D. & Salvaterra, P. M. Brain aging and Abeta(1)(-)(4)(2) neurotoxicity converge via deterioration in autophagy-lysosomal system: a conditional Drosophila model linking Alzheimer’s neurodegeneration with aging. *Acta Neuropathol.* **121**, 183–191 (2011). 21076961 10.1007/s00401-010-0772-0

[297] Caccamo, A. et al. Molecular interplay between mammalian target of rapamycin (mTOR), amyloid-beta, and Tau: effects on cognitive impairments. *J. Biol. Chem.* **285**, 13107–13120 (2010). 20178983 10.1074/jbc.M110.100420 PMC2857107

[298] Cai, Z. et al. Roles of AMP-activated protein kinase in Alzheimer’s disease. *Neuromology* **14**, 1–14 (2012). 10.1007/s12017-012-8173-2 22367557

[299] Lee, A. et al. Abeta42 oligomers trigger synaptic loss through CAMKK2-AMPK-dependent effectors coordinating mitochondrial fission and mitophagy. *Nat. Commun.* **13**, 4444 (2022). 35915085 10.1038/s41467-022-32130-5 PMC9343354

[300] Berg, D. et al. Changing the research criteria for the diagnosis of Parkinson’s disease: obstacles and opportunities. *Lancet Neurol.* **12**, 514–524 (2013). 23582175 10.1016/S1474-4422(13)70047-4

[301] Tang, F. et al. Inhibition of ACSL4 alleviates parkinsonism phenotypes by reduction of lipid reactive oxygen species. *Neurotherapeutics* **20**, 1154–1166 (2023). 37133631 10.1007/s13311-023-01382-4 PMC10457271

[302] Jankovic, J. & Tan, E. K. Parkinson’s disease: etiopathogenesis and treatment. *J. Neurol. Neurosurg. Psychiatry* **91**, 795–808 (2020). 32576618 10.1136/jnnp-2019-322338

[303] Collaborators, G. B. D. N. Global, regional, and national burden of neurological disorders, 1990–2016: a systematic analysis for the Global Burden of Disease Study 2016. *Lancet Neurol.* **18**, 459–480 (2019). 30879893 10.1016/S1474-4422(18)30499-X PMC6459001

[304] Aarsland, D. et al. Parkinson disease-associated cognitive impairment. *Nat. Rev. Dis. Prim.* **7**, 47 (2021). 34210995 10.1038/s41572-021-00280-3

[305] Guo, Y. J. et al. Brain regions susceptible to alpha-synuclein spreading. *Mol. Psychiatry* **27**, 758–770 (2022). 34561613 10.1038/s41380-021-01296-7

[306] Santos Garcia, D. et al. Non-motor symptoms burden, mood, and gait problems are the most significant factors contributing to a poor quality of life in non-demented Parkinson’s disease patients: Results from the COPPADIS Study Cohort. *Parkinsonism Relat. Disord.* **66**, 151–157 (2019). 31409572 10.1016/j.parkreldis.2019.07.031

[307] Bloem, B. R., Okun, M. S. & Klein, C. Parkinson’s disease. *Lancet* **397**, 2284–2303 (2021). 33848468 10.1016/S0140-6736(21)00218-X

[308] Chen, K., Guo, Y.-J., Lei, P. & Finkelstein, D. I. Can alpha-synuclein be both the cause and a consequence of Parkinson’s disease? *Ageing Neurodegener. Dis*. **3**, 10 (2023).

[309] Shahmoradian, S. H. et al. Lewy pathology in Parkinson’s disease consists of crowded organelles and lipid membranes. *Nat. Neurosci.* **22**, 1099–1109 (2019). 31235907 10.1038/s41593-019-0423-2

[310] Jankovic, J. et al. Safety and tolerability of multiple ascending doses of PRX002/RG7935, an anti-alpha-synuclein monoclonal antibody, in patients with Parkinson's Disease: a randomized clinical trial. *JAMA Neurol.* **75**, 1206–1214 (2018). 29913017 10.1001/jamaneurol.2018.1487 PMC6233845

[311] Dunning, C. J., George, S. & Brundin, P. What’s to like about the prion-like hypothesis for the spreading of aggregated alpha-synuclein in Parkinson's Disease? *Prion* **7**, 92–97 (2013). 23360753 10.4161/pri.23806 PMC3609056

[312] He, S. et al. Effects of alpha-synuclein-associated post-translational modifications in Parkinson’s Disease. *ACS Chem. Neurosci.* **12**, 1061–1071 (2021). 33769791 10.1021/acschemneuro.1c00028

[313] Dzamko, N., Zhou, J., Huang, Y. & Halliday, G. M. Parkinson’s disease-implicated kinases in the brain; insights into disease pathogenesis. *Front. Mol. Neurosci.* **7**, 57 (2014). 25009465 10.3389/fnmol.2014.00057 PMC4068290

[314] Chen, L. & Feany, M. B. Alpha-synuclein phosphorylation controls neurotoxicity and inclusion formation in a Drosophila model of Parkinson's disease. *Nat. Neurosci.* **8**, 657–663 (2005). 15834418 10.1038/nn1443

[315] Febbraro, F. et al. Ser129D mutant alpha-synuclein induces earlier motor dysfunction while S129A results in distinctive pathology in a rat model of Parkinson’s disease. *Neurobiol. Dis.* **56**, 47–58 (2013). 23567651 10.1016/j.nbd.2013.03.014

[316] McFarland, N. R. et al. Alpha-synuclein S129 phosphorylation mutants do not alter nigrostriatal toxicity in a rat model of Parkinson's disease. *J. Neuropathol. Exp. Neurol.* **68**, 515–524 (2009). 19525899 10.1097/NEN.0b013e3181a24b53 PMC2753269

[317] Sato, H., Kato, T. & Arawaka, S. The role of Ser129 phosphorylation of alpha-synuclein in neurodegeneration of Parkinson’s disease: a review of in vivo models. *Rev. Neurosci.* **24**, 115–123 (2013). 23314528 10.1515/revneuro-2012-0071

[318] Fujiwara, H. et al. alpha-Synuclein is phosphorylated in synucleinopathy lesions. *Nat. Cell Biol.* **4**, 160–164 (2002). 11813001 10.1038/ncb748

[319] Arawaka, S. et al. The role of G-protein-coupled receptor kinase 5 in pathogenesis of sporadic Parkinson’s disease. *J. Neurosci.* **26**, 9227–9238 (2006). 16957079 10.1523/JNEUROSCI.0341-06.2006 PMC6674490

[320] Jellinger, K. A. Neuropathology of sporadic Parkinson’s disease: evaluation and changes of concepts. *Mov. Disord.* **27**, 8–30 (2012). 22081500 10.1002/mds.23795

[321] Fearnley, J. M. & Lees, A. J. Ageing and Parkinson’s disease: substantia nigra regional selectivity. *Brain* **114**, 2283–2301 (1991). 1933245 10.1093/brain/114.5.2283

[322] Pacelli, C. et al. Elevated mitochondrial bioenergetics and axonal arborization size are key contributors to the vulnerability of dopamine neurons. *Curr. Biol.* **25**, 2349–2360 (2015). 26320949 10.1016/j.cub.2015.07.050

[323] Matsuda, W. et al. Single nigrostriatal dopaminergic neurons form widely spread and highly dense axonal arborizations in the neostriatum. *J. Neurosci.* **29**, 444–453 (2009). 19144844 10.1523/JNEUROSCI.4029-08.2009 PMC6664950

[324] Polymeropoulos, M. H. et al. Mutation in the alpha-synuclein gene identified in families with Parkinson’s disease. *Science* **276**, 2045–2047 (1997). 9197268 10.1126/science.276.5321.2045

[325] Pihlstrom, L. & Toft, M. Genetic variability in SNCA and Parkinson’s disease. *Neurogenetics* **12**, 283–293 (2011). 21800132 10.1007/s10048-011-0292-7

[326] Trinh, J. et al. Genotype-phenotype relations for the Parkinson’s disease genes SNCA, LRRK2, VPS35: MDS gene systematic review. *Mov. Disord.* **33**, 1857–1870 (2018). 30357936 10.1002/mds.27527

[327] Jha, S. K. et al. p38 MAPK and PI3K/AKT signalling cascades in Parkinson’s Disease. *Int. J. Mol. Cell Med.* **4**, 67–86 (2015). 26261796 PMC4499569

[328] Morrison, R. S. et al. Neuronal survival and cell death signaling pathways. *Adv. Exp. Med. Biol.* **513**, 41–86 (2002). 12575817 10.1007/978-1-4615-0123-7_2

[329] Di Maio, R. et al. LRRK2 activation in idiopathic Parkinson’s disease. *Sci. Transl. Med*. **10**, eaar5429 (2018). 10.1126/scitranslmed.aar5429 PMC6344941 30045977

[330] Aasly, J. O. et al. Clinical features of LRRK2-associated Parkinson’s disease in central Norway. *Ann. Neurol.* **57**, 762–765 (2005). 15852371 10.1002/ana.20456

[331] Marras, C. et al. Phenotype in parkinsonian and nonparkinsonian LRRK2 G2019S mutation carriers. *Neurology* **77**, 325–333 (2011). 21753163 10.1212/WNL.0b013e318227042d PMC3140802

[332] Bonet-Ponce, L. et al. LRRK2 mediates tubulation and vesicle sorting from lysosomes. *Sci Adv*. **6**, eabb2454 (2020). 10.1126/sciadv.abb2454 PMC7673727 33177079

[333] Steger, M. et al. Phosphoproteomics reveals that Parkinson’s disease kinase LRRK2 regulates a subset of Rab GTPases. *Elife*. **5**, e12813 (2016). 10.7554/eLife.12813 PMC4769169 26824392

[334] Vazquez-Velez, G. E. & Zoghbi, H. Y. Parkinson’s disease genetics and pathophysiology. *Annu. Rev. Neurosci.* **44**, 87–108 (2021). 34236893 10.1146/annurev-neuro-100720-034518

[335] Orenstein, S. J. et al. Interplay of LRRK2 with chaperone-mediated autophagy. *Nat. Neurosci.* **16**, 394–406 (2013). 23455607 10.1038/nn.3350 PMC3609872

[336] Nguyen, M. & Krainc, D. LRRK2 phosphorylation of auxilin mediates synaptic defects in dopaminergic neurons from patients with Parkinson’s disease. *Proc. Natl. Acad. Sci. USA* **115**, 5576–5581 (2018). 29735704 10.1073/pnas.1717590115 PMC6003526

[337] Herbst, S. et al. LRRK2 activation controls the repair of damaged endomembranes in macrophages. *EMBO J.* **39**, e104494 (2020). 32643832 10.15252/embj.2020104494 PMC7507578

[338] West, A. B. Achieving neuroprotection with LRRK2 kinase inhibitors in Parkinson disease. *Exp. Neurol.* **298**, 236–245 (2017). 28764903 10.1016/j.expneurol.2017.07.019 PMC5693612

[339] Chan, S. L. & Tan, E. K. Targeting LRRK2 in Parkinson’s disease: an update on recent developments. *Expert Opin. Ther. Targets* **21**, 601–610 (2017). 28443359 10.1080/14728222.2017.1323881

[340] Shihabuddin, L. S. et al. New frontiers in Parkinson’s Disease: from genetics to the clinic. *J. Neurosci.* **38**, 9375–9382 (2018). 30381429 10.1523/JNEUROSCI.1666-18.2018 PMC6705997

[341] Chen, K. et al. Leucine-rich repeat kinase 2 (LRRK2) inhibition upregulates microtubule-associated protein 1B to ameliorate lysosomal dysfunction and parkinsonism. *MedComm* **4**, e429 (2023). 38020716 10.1002/mco2.429 PMC10661827

[342] Zhao, H. T. et al. LRRK2 antisense oligonucleotides ameliorate alpha-synuclein inclusion formation in a Parkinson’s Disease mouse model. *Mol. Ther. Nucleic Acids* **8**, 508–519 (2017). 28918051 10.1016/j.omtn.2017.08.002 PMC5573879

[343] Jennings, D. et al. Preclinical and clinical evaluation of the LRRK2 inhibitor DNL201 for Parkinson’s disease. *Sci. Transl. Med.* **14**, eabj2658 (2022). 35675433 10.1126/scitranslmed.abj2658

[344] Jennings, D. et al. LRRK2 inhibition by BIIB122 in healthy participants and patients with Parkinson’s disease. *Mov. Disord.* **38**, 386–398 (2023). 36807624 10.1002/mds.29297

[345] Cao, R. et al. Recent advances in targeting leucine-rich repeat kinase 2 as a potential strategy for the treatment of Parkinson’s disease. *Bioorg. Chem.* **141**, 106906 (2023). 37837728 10.1016/j.bioorg.2023.106906

[346] Misgeld, T. & Schwarz, T. L. Mitostasis in neurons: maintaining mitochondria in an extended cellular architecture. *Neuron* **96**, 651–666 (2017). 29096078 10.1016/j.neuron.2017.09.055 PMC5687842

[347] Dawson, T. M. & Dawson, V. L. Molecular pathways of neurodegeneration in Parkinson’s disease. *Science* **302**, 819–822 (2003). 14593166 10.1126/science.1087753

[348] Schapira, A. H. et al. Mitochondrial complex I deficiency in Parkinson’s disease. *Lancet* **1**, 1269 (1989). 2566813 10.1016/s0140-6736(89)92366-0

[349] Giannoccaro, M. P., La Morgia, C., Rizzo, G. & Carelli, V. Mitochondrial DNA and primary mitochondrial dysfunction in Parkinson’s disease. *Mov. Disord.* **32**, 346–363 (2017). 28251677 10.1002/mds.26966

[350] Clark, E. H., Vazquez de la Torre, A., Hoshikawa, T. & Briston, T. Targeting mitophagy in Parkinson’s disease. *J. Biol. Chem.* **296**, 100209 (2021). 33372898 10.1074/jbc.REV120.014294 PMC7948953

[351] Vives-Bauza, C. et al. PINK1-dependent recruitment of Parkin to mitochondria in mitophagy. *Proc. Natl. Acad. Sci. USA* **107**, 378–383 (2010). 19966284 10.1073/pnas.0911187107 PMC2806779

[352] Rakovic, A. et al. PINK1-dependent mitophagy is driven by the UPS and can occur independently of LC3 conversion. *Cell Death Differ.* **26**, 1428–1441 (2019). 30375512 10.1038/s41418-018-0219-z PMC6748138

[353] Harper, J. W., Ordureau, A. & Heo, J. M. Building and decoding ubiquitin chains for mitophagy. *Nat. Rev. Mol. Cell Biol.* **19**, 93–108 (2018). 29358684 10.1038/nrm.2017.129

[354] Ge, P., Dawson, V. L. & Dawson, T. M. PINK1 and Parkin mitochondrial quality control: a source of regional vulnerability in Parkinson’s disease. *Mol. Neurodegener.* **15**, 20 (2020). 32169097 10.1186/s13024-020-00367-7 PMC7071653

[355] Matsuda, N. & Tanaka, K. Uncovering the roles of PINK1 and parkin in mitophagy. *Autophagy* **6**, 952–954 (2010). 20724841 10.4161/auto.6.7.13039 PMC3039741

[356] Kasten, M. et al. Genotype-phenotype relations for the Parkinson’s Disease genes parkin, PINK1, DJ1: MDSGene systematic review. *Mov. Disord.* **33**, 730–741 (2018). 29644727 10.1002/mds.27352

[357] Kitada, T. et al. Mutations in the parkin gene cause autosomal recessive juvenile parkinsonism. *Nature* **392**, 605–608 (1998). 9560156 10.1038/33416

[358] Valente, E. M. et al. Hereditary early-onset Parkinson’s disease caused by mutations in PINK1. *Science* **304**, 1158–1160 (2004). 15087508 10.1126/science.1096284

[359] Beilina, A. et al. Mutations in PTEN-induced putative kinase 1 associated with recessive parkinsonism have differential effects on protein stability. *Proc. Natl. Acad. Sci. USA* **102**, 5703–5708 (2005). 15824318 10.1073/pnas.0500617102 PMC556294

[360] Sim, C. H. et al. C-terminal truncation and Parkinson’s disease-associated mutations down-regulate the protein serine/threonine kinase activity of PTEN-induced kinase-1. *Hum. Mol. Genet*.**15**, 3251–3262 (2006). 17000703 10.1093/hmg/ddl398

[361] Wang, H. L. et al. PINK1 mutants associated with recessive Parkinson’s disease are defective in inhibiting mitochondrial release of cytochrome C. *Neurobiol. Dis.* **28**, 216–226 (2007). 17707122 10.1016/j.nbd.2007.07.010

[362] Ko, H. S. et al. Accumulation of the authentic parkin substrate aminoacyl-tRNA synthetase cofactor, p38/JTV-1, leads to catecholaminergic cell death. *J. Neurosci.* **25**, 7968–7978 (2005). 16135753 10.1523/JNEUROSCI.2172-05.2005 PMC6725452

[363] Lee, Y. et al. Parthanatos mediates AIMP2-activated age-dependent dopaminergic neuronal loss. *Nat. Neurosci.* **16**, 1392–1400 (2013). 23974709 10.1038/nn.3500 PMC3785563

[364] Ko, H. S. et al. Phosphorylation by the c-Abl protein tyrosine kinase inhibits Parkin’s ubiquitination and protective function. *Proc. Natl. Acad. Sci. USA* **107**, 16691–16696 (2010). 20823226 10.1073/pnas.1006083107 PMC2944759

[365] Pankratz, N. et al. Genomewide association study for susceptibility genes contributing to familial Parkinson disease. *Hum. Genet.* **124**, 593–605 (2009). 18985386 10.1007/s00439-008-0582-9 PMC2627511

[366] Rhodes, S. L. et al. Replication of GWAS associations for GAK and MAPT in Parkinson’s disease. *Ann. Hum. Genet.* **75**, 195–200 (2011). 21058943 10.1111/j.1469-1809.2010.00616.x PMC3074465

[367] Li, N. N. et al. GWAS-linked GAK locus in Parkinson’s disease in Han Chinese and meta-analysis. *Hum. Genet.* **131**, 1089–1093 (2012). 22198721 10.1007/s00439-011-1133-3

[368] Li, H., Zhang, C. & Ji, Y. Association of GAK rs1564282 With Susceptibility to Parkinson’s Disease in Chinese populations. *Front. Genet.* **12**, 777942 (2021). 34868266 10.3389/fgene.2021.777942 PMC8637629

[369] Shulman, J. M. et al. Association of Parkinson disease risk loci with mild parkinsonian signs in older persons. *JAMA Neurol.* **71**, 429–435 (2014). 24514572 10.1001/jamaneurol.2013.6222 PMC4039209

[370] Eisenberg, E. & Greene, L. E. Multiple roles of auxilin and hsc70 in clathrin-mediated endocytosis. *Traffic* **8**, 640–646 (2007). 17488288 10.1111/j.1600-0854.2007.00568.x

[371] Lee, D. W. et al. Essential role of cyclin-G-associated kinase (Auxilin-2) in developing and mature mice. *Mol. Biol. Cell* **19**, 2766–2776 (2008). 18434600 10.1091/mbc.E07-11-1115 PMC2441687

[372] Egawa, J. et al. The cyclin G-associated kinase (GAK) inhibitor SGC-GAK-1 inhibits neurite outgrowth and synapse formation. *Mol. Brain* **15**, 68 (2022). 35883152 10.1186/s13041-022-00951-6 PMC9327206

[373] Munson, M. J. et al. GAK and PRKCD are positive regulators of PRKN-independent mitophagy. *Nat. Commun.* **12**, 6101 (2021). 34671015 10.1038/s41467-021-26331-7 PMC8528926

[374] Song, L. et al. Auxilin underlies progressive locomotor deficits and dopaminergic neuron loss in a drosophila model of Parkinson’s Disease. *Cell Rep.* **18**, 1132–1143 (2017). 28147270 10.1016/j.celrep.2017.01.005

[375] Dumitriu, A. et al. Cyclin-G-associated kinase modifies alpha-synuclein expression levels and toxicity in Parkinson’s disease: results from the GenePD study. *Hum. Mol. Genet.* **20**, 1478–1487 (2011). 21258085 10.1093/hmg/ddr026 PMC3063983

[376] Ali, M. Z. & Dholaniya, P. S. Oxidative phosphorylation mediated pathogenesis of Parkinson’s disease and its implication via Akt signaling. *Neurochem. Int.* **157**, 105344 (2022). 35483538 10.1016/j.neuint.2022.105344

[377] Koutsilieri, E., Chan, W. W., Reinitzer, D. & Rausch, W. D. Functional changes in cocultures of mesencephalon and striatal neurons from embryonic C57/BL6 mice due to low concentrations of 1-methyl-4-phenylpyridinium (MPP+). *J. Neural Transm. Gen. Sect.* **94**, 189–197 (1993). 7907217 10.1007/BF01277024

[378] Akundi, R. S., Zhi, L. & Bueler, H. PINK1 enhances insulin-like growth factor-1-dependent Akt signaling and protection against apoptosis. *Neurobiol. Dis.* **45**, 469–478 (2012). 21945539 10.1016/j.nbd.2011.08.034 PMC3225697

[379] Boonying, W. et al. Pink1 regulates FKBP5 interaction with AKT/PHLPP and protects neurons from neurotoxin stress induced by MPP. *J. Neurochem.* **150**, 312–329 (2019). 30734931 10.1111/jnc.14683

[380] Morais, V. A. et al. Parkinson’s disease mutations in PINK1 result in decreased complex I activity and deficient synaptic function. *EMBO Mol. Med.* **1**, 99–111 (2009). 20049710 10.1002/emmm.200900006 PMC3378121

[381] Yasuda, T. et al. Parkin-mediated protection of dopaminergic neurons in a chronic MPTP-minipump mouse model of Parkinson's disease. *J. Neuropathol. Exp. Neurol.* **70**, 686–697 (2011). 21760537 10.1097/NEN.0b013e3182269ecd

[382] Chuang, C. L., Lu, Y. N., Wang, H. C. & Chang, H. Y. Genetic dissection reveals that Akt is the critical kinase downstream of LRRK2 to phosphorylate and inhibit FOXO1, and promotes neuron survival. *Hum. Mol. Genet* **23**, 5649–5658 (2014). 24916379 10.1093/hmg/ddu281

[383] Neves, M., Graos, M., Anjo, S. I. & Manadas, B. Modulation of signaling pathways by DJ-1: An updated overview. *Redox Biol.* **51**, 102283 (2022). 35303520 10.1016/j.redox.2022.102283 PMC8928136

[384] van Duijn, C. M. et al. Park7, a novel locus for autosomal recessive early-onset parkinsonism, on chromosome 1p36. *Am. J. Hum. Genet.* **69**, 629–634 (2001). 11462174 10.1086/322996 PMC1235491

[385] Oh, S. E. & Mouradian, M. M. Cytoprotective mechanisms of DJ-1 against oxidative stress through modulating ERK1/2 and ASK1 signal transduction. *Redox Biol.* **14**, 211–217 (2018). 28954246 10.1016/j.redox.2017.09.008 PMC5614756

[386] Takahashi-Niki, K. et al. Epidermal growth factor-dependent activation of the extracellular signal-regulated kinase pathway by DJ-1 protein through its direct binding to c-Raf protein. *J. Biol. Chem.* **290**, 17838–17847 (2015). 26048984 10.1074/jbc.M115.666271 PMC4505034

[387] Kim, R. H. et al. DJ-1, a novel regulator of the tumor suppressor PTEN. *Cancer Cell* **7**, 263–273 (2005). 15766664 10.1016/j.ccr.2005.02.010

[388] Xia, X. G. et al. Gene transfer of the JNK interacting protein-1 protects dopaminergic neurons in the MPTP model of Parkinson’s disease. *Proc. Natl. Acad. Sci. USA* **98**, 10433–10438 (2001). 11504916 10.1073/pnas.181182298 PMC56978

[389] Pan, J. et al. Expression of FasL and its interaction with Fas are mediated by c-Jun N-terminal kinase (JNK) pathway in 6-OHDA-induced rat model of Parkinson disease. *Neurosci. Lett.* **428**, 82–87 (2007). 17959308 10.1016/j.neulet.2007.09.032

[390] Saporito, M. S., Brown, E. M., Miller, M. S. & Carswell, S. CEP-1347/KT-7515, an inhibitor of c-jun N-terminal kinase activation, attenuates the 1-methyl-4-phenyl tetrahydropyridine-mediated loss of nigrostriatal dopaminergic neurons In vivo. *J. Pharm. Exp. Ther.* **288**, 421–427 (1999). 9918541

[391] Chen, J. et al. Phosphorylation of Parkin at serine 131 by p38 MAPK promotes mitochondrial dysfunction and neuronal death in mutant A53T alpha-synuclein model of Parkinson’s disease. *Cell Death Dis.* **9**, 700 (2018). 29899409 10.1038/s41419-018-0722-7 PMC5999948

[392] Tu, H. Y. et al. alpha-synuclein suppresses microglial autophagy and promotes neurodegeneration in a mouse model of Parkinson’s disease. *Aging Cell* **20**, e13522 (2021). 34811872 10.1111/acel.13522 PMC8672776

[393] Hunot, S. et al. JNK-mediated induction of cyclooxygenase 2 is required for neurodegeneration in a mouse model of Parkinson’s disease. *Proc. Natl. Acad. Sci. USA* **101**, 665–670 (2004). 14704277 10.1073/pnas.0307453101 PMC327205

[394] Teismann, P. et al. Cyclooxygenase-2 is instrumental in Parkinson’s disease neurodegeneration. *Proc. Natl. Acad. Sci. USA* **100**, 5473–5478 (2003). 12702778 10.1073/pnas.0837397100 PMC154369

[395] Gao, F., Chen, D., Hu, Q. & Wang, G. Rotenone directly induces BV2 cell activation via the p38 MAPK pathway. *PLoS One* **8**, e72046 (2013). 23977201 10.1371/journal.pone.0072046 PMC3748029

[396] Gloeckner, C. J., Schumacher, A., Boldt, K. & Ueffing, M. The Parkinson disease-associated protein kinase LRRK2 exhibits MAPKKK activity and phosphorylates MKK3/6 and MKK4/7, in vitro. *J. Neurochem.* **109**, 959–968 (2009). 19302196 10.1111/j.1471-4159.2009.06024.x

[397] Hsu, C. H., Chan, D. & Wolozin, B. LRRK2 and the stress response: interaction with MKKs and JNK-interacting proteins. *Neurodegener. Dis.* **7**, 68–75 (2010). 20173330 10.1159/000285509 PMC2859233

[398] Iba, M. et al. Inhibition of p38alpha MAPK restores neuronal p38gamma MAPK and ameliorates synaptic degeneration in a mouse model of DLB/PD. *Sci. Transl. Med.* **15**, eabq6089 (2023). 37163617 10.1126/scitranslmed.abq6089 PMC12168722

[399] Chen, J. et al. p38-TFEB pathways promote microglia activation through inhibiting CMA-mediated NLRP3 degradation in Parkinson’s disease. *J. Neuroinflamm.* **18**, 295 (2021). 10.1186/s12974-021-02349-y PMC8686293 34930303

[400] Zawada, W. M. et al. Inhibitors of p38 MAP kinase increase the survival of transplanted dopamine neurons. *Brain Res.* **891**, 185–196 (2001). 11164822 10.1016/s0006-8993(00)02965-6

[401] Parkinson Study Group, P. I. Mixed lineage kinase inhibitor CEP-1347 fails to delay disability in early Parkinson disease. *Neurology* **69**, 1480–1490 (2007). 17881719 10.1212/01.wnl.0000277648.63931.c0

[402] Ichijo, H. et al. Induction of apoptosis by ASK1, a mammalian MAPKKK that activates SAPK/JNK and p38 signaling pathways. *Science* **275**, 90–94 (1997). 8974401 10.1126/science.275.5296.90

[403] Ray, A. et al. MPTP activates ASK1-p38 MAPK signaling pathway through TNF-dependent Trx1 oxidation in Parkinsonism mouse model. *Free Radic. Biol. Med.* **87**, 312–325 (2015). 26164633 10.1016/j.freeradbiomed.2015.06.041

[404] Lee, K. W. et al. Apoptosis signal-regulating kinase 1 mediates MPTP toxicity and regulates glial activation. *PLoS One* **7**, e29935 (2012). 22253830 10.1371/journal.pone.0029935 PMC3254627

[405] Ortner, E. & Moelling, K. Heteromeric complex formation of ASK2 and ASK1 regulates stress-induced signaling. *Biochem. Biophys. Res. Commun.* **362**, 454–459 (2007). 17714688 10.1016/j.bbrc.2007.08.006

[406] Mansour, H. M., Mohamed, A. F., El-Khatib, A. S. & Khattab, M. M. Kinases control of regulated cell death revealing druggable targets for Parkinson’s disease. *Ageing Res. Rev.* **85**, 101841 (2023). 36608709 10.1016/j.arr.2022.101841

[407] Zhu, J. et al. Apelin-36 mediates neuroprotective effects by regulating oxidative stress, autophagy and apoptosis in MPTP-induced Parkinson’s disease model mice. *Brain Res.* **1726**, 146493 (2020). 31586624 10.1016/j.brainres.2019.146493

[408] Lee, K. W. et al. Apoptosis signal-regulating kinase 1 modulates the phenotype of alpha-synuclein transgenic mice. *Neurobiol. Aging* **36**, 519–526 (2015). 25219466 10.1016/j.neurobiolaging.2014.07.034 PMC4268347

[409] Zhang, J. et al. Apoptosis signal-regulating kinase 1 deletion mitigates alpha-synuclein pre-formed fibril propagation in mice. *Neurobiol. Aging* **85**, 49–57 (2020). 31734439 10.1016/j.neurobiolaging.2019.09.012 PMC7064162

[410] Yoon, J. H. et al. LRRK2 functions as a scaffolding kinase of ASK1-mediated neuronal cell death. *Biochim. Biophys. Acta Mol. Cell Res.* **1864**, 2356–2368 (2017). 28888991 10.1016/j.bbamcr.2017.09.001

[411] Junn, E. et al. Interaction of DJ-1 with Daxx inhibits apoptosis signal-regulating kinase 1 activity and cell death. *Proc. Natl. Acad. Sci. USA* **102**, 9691–9696 (2005). 15983381 10.1073/pnas.0409635102 PMC1172235

[412] Chertow, G. M. et al. Effects of selonsertib in patients with diabetic kidney disease. *J. Am. Soc. Nephrol.* **30**, 1980–1990 (2019). 31506292 10.1681/ASN.2018121231 PMC6779369

[413] Hou, S. et al. Structure-based discovery of 1H-indole-2-carboxamide derivatives as potent ASK1 inhibitors for potential treatment of ulcerative colitis. *Eur. J. Med. Chem.* **211**, 113114 (2021). 33360793 10.1016/j.ejmech.2020.113114

[414] Liles, J. T. et al. ASK1 contributes to fibrosis and dysfunction in models of kidney disease. *J. Clin. Investig.* **128**, 4485–4500 (2018). 30024858 10.1172/JCI99768 PMC6159961

[415] Bowles, K. R. & Jones, L. Kinase signalling in Huntington’s disease. *J. Huntingt. Dis.* **3**, 89–123 (2014). 10.3233/JHD-140106 25062854

[416] Andrew, S. E. et al. The relationship between trinucleotide (CAG) repeat length and clinical features of Huntington’s disease. *Nat. Genet.* **4**, 398–403 (1993). 8401589 10.1038/ng0893-398

[417] Squitieri, F. et al. Homozygosity for CAG mutation in Huntington disease is associated with a more severe clinical course. *Brain* **126**, 946–955 (2003). 12615650 10.1093/brain/awg077

[418] Tabrizi, S. J., Flower, M. D., Ross, C. A. & Wild, E. J. Huntington disease: new insights into molecular pathogenesis and therapeutic opportunities. *Nat. Rev. Neurol.* **16**, 529–546 (2020). 32796930 10.1038/s41582-020-0389-4

[419] Roos, R. A. Huntington’s disease: a clinical review. *Orphanet J. Rare Dis.* **5**, 40 (2010). 21171977 10.1186/1750-1172-5-40 PMC3022767

[420] Leavitt, B. R., Kordasiewicz, H. B. & Schobel, S. A. Huntingtin-lowering therapies for Huntington disease: a review of the evidence of potential benefits and risks. *JAMA Neurol.* **77**, 764–772 (2020). 32202594 10.1001/jamaneurol.2020.0299

[421] Caron, N. S., Dorsey, E. R. & Hayden, M. R. Therapeutic approaches to Huntington disease: from the bench to the clinic. *Nat. Rev. Drug Discov.* **17**, 729–750 (2018). 30237454 10.1038/nrd.2018.133

[422] Evans, S. J. et al. Prevalence of adult Huntington’s disease in the UK based on diagnoses recorded in general practice records. *J. Neurol. Neurosurg. Psychiatry* **84**, 1156–1160 (2013). 23482661 10.1136/jnnp-2012-304636 PMC3786631

[423] Arrasate, M. & Finkbeiner, S. Protein aggregates in Huntington’s disease. *Exp. Neurol.* **238**, 1–11 (2012). 22200539 10.1016/j.expneurol.2011.12.013 PMC3909772

[424] Schilling, B. et al. Huntingtin phosphorylation sites mapped by mass spectrometry. Modulation of cleavage and toxicity. *J. Biol. Chem.* **281**, 23686–23697 (2006). 16782707 10.1074/jbc.M513507200

[425] Gu, X. et al. Serines 13 and 16 are critical determinants of full-length human mutant Huntingtin-induced disease pathogenesis in HD mice. *Neuron* **64**, 828–840 (2009). 20064390 10.1016/j.neuron.2009.11.020 PMC2807408

[426] Luo, S., Vacher, C., Davies, J. E. & Rubinsztein, D. C. Cdk5 phosphorylation of huntingtin reduces its cleavage by caspases: implications for mutant huntingtin toxicity. *J. Cell Biol.* **169**, 647–656 (2005). 15911879 10.1083/jcb.200412071 PMC2171695

[427] Thompson, L. M. et al. IKK phosphorylates Huntingtin and targets it for degradation by the proteasome and lysosome. *J. Cell Biol.* **187**, 1083–1099 (2009). 20026656 10.1083/jcb.200909067 PMC2806289

[428] Hegde, R. N. et al. TBK1 phosphorylates mutant Huntingtin and suppresses its aggregation and toxicity in Huntington’s disease models. *EMBO J.* **39**, e104671 (2020). 32757223 10.15252/embj.2020104671 PMC7459410

[429] Humbert, S. et al. The IGF-1/Akt pathway is neuroprotective in Huntington’s disease and involves Huntingtin phosphorylation by Akt. *Dev. Cell* **2**, 831–837 (2002). 12062094 10.1016/s1534-5807(02)00188-0

[430] Apostol, B. L. et al. Mutant huntingtin alters MAPK signaling pathways in PC12 and striatal cells: ERK1/2 protects against mutant huntingtin-associated toxicity. *Hum. Mol. Genet.* **15**, 273–285 (2006). 16330479 10.1093/hmg/ddi443

[431] Merienne, K. et al. Polyglutamine expansion induces a protein-damaging stress connecting heat shock protein 70 to the JNK pathway. *J. Biol. Chem.* **278**, 16957–16967 (2003). 12598532 10.1074/jbc.M212049200

[432] Saavedra, A. et al. Striatal-enriched protein tyrosine phosphatase expression and activity in Huntington’s disease: a STEP in the resistance to excitotoxicity. *J. Neurosci.* **31**, 8150–8162 (2011). 21632937 10.1523/JNEUROSCI.3446-10.2011 PMC3472648

[433] Lievens, J. C., Woodman, B., Mahal, A. & Bates, G. P. Abnormal phosphorylation of synapsin I predicts a neuronal transmission impairment in the R6/2 Huntington’s disease transgenic mice. *Mol. Cell Neurosci.* **20**, 638–648 (2002). 12213445 10.1006/mcne.2002.1152

[434] Liot, G. et al. Mutant Huntingtin alters retrograde transport of TrkB receptors in striatal dendrites. *J. Neurosci.* **33**, 6298–6309 (2013). 23575829 10.1523/JNEUROSCI.2033-12.2013 PMC6619069

[435] Bodai, L. & Marsh, J. L. A novel target for Huntington’s disease: ERK at the crossroads of signaling. The ERK signaling pathway is implicated in Huntington’s disease and its upregulation ameliorates pathology. *Bioessays* **34**, 142–148 (2012). 22334892 10.1002/bies.201100116 PMC3711381

[436] Roze, E. et al. Mitogen- and stress-activated protein kinase-1 deficiency is involved in expanded-huntingtin-induced transcriptional dysregulation and striatal death. *FASEB J.* **22**, 1083–1093 (2008). 18029446 10.1096/fj.07-9814

[437] Wang, P. et al. The KDEL receptor induces autophagy to promote the clearance of neurodegenerative disease-related proteins. *Neuroscience* **190**, 43–55 (2011). 21684323 10.1016/j.neuroscience.2011.06.008

[438] Hacker, H. & Karin, M. Regulation and function of IKK and IKK-related kinases. *Sci. STKE* **2006**, re13 (2006). 17047224 10.1126/stke.3572006re13

[439] Khoshnan, A. et al. Activation of the IkappaB kinase complex and nuclear factor-kappaB contributes to mutant huntingtin neurotoxicity. *J. Neurosci.* **24**, 7999–8008 (2004). 15371500 10.1523/JNEUROSCI.2675-04.2004 PMC6729796

[440] Trager, U. et al. HTT-lowering reverses Huntington’s disease immune dysfunction caused by NFkappaB pathway dysregulation. *Brain* **137**, 819–833 (2014). 24459107 10.1093/brain/awt355 PMC3983408

[441] Marcora, E. & Kennedy, M. B. The Huntington’s disease mutation impairs Huntingtin’s role in the transport of NF-kappaB from the synapse to the nucleus. *Hum. Mol. Genet* **19**, 4373–4384 (2010). 20739295 10.1093/hmg/ddq358 PMC2957321

[442] Singh, S. & Singh, T. G. Role of nuclear factor Kappa B (NF-kappaB) signalling in neurodegenerative diseases: an mechanistic approach. *Curr. Neuropharmacol.* **18**, 918–935 (2020). 32031074 10.2174/1570159X18666200207120949 PMC7709146

[443] Bjorkqvist, M. et al. A novel pathogenic pathway of immune activation detectable before clinical onset in Huntington’s disease. *J. Exp. Med.* **205**, 1869–1877 (2008). 18625748 10.1084/jem.20080178 PMC2525598

[444] Zuccato, C., Valenza, M. & Cattaneo, E. Molecular mechanisms and potential therapeutical targets in Huntington’s disease. *Physiol. Rev.* **90**, 905–981 (2010). 20664076 10.1152/physrev.00041.2009

[445] Landles, C. et al. Proteolysis of mutant huntingtin produces an exon 1 fragment that accumulates as an aggregated protein in neuronal nuclei in Huntington disease. *J. Biol. Chem.* **285**, 8808–8823 (2010). 20086007 10.1074/jbc.M109.075028 PMC2838303

[446] Ochaba, J. et al. IKKbeta slows Huntington’s disease progression in R6/1 mice. *Proc. Natl Acad. Sci. USA* **116**, 10952–10961 (2019). 31088970 10.1073/pnas.1814246116 PMC6561205

[447] Kane, L. P., Shapiro, V. S., Stokoe, D. & Weiss, A. Induction of NF-kappaB by the Akt/PKB kinase. *Curr. Biol.* **9**, 601–604 (1999). 10359702 10.1016/s0960-9822(99)80265-6

[448] Meng, F., Liu, L., Chin, P. C. & D’Mello, S. R. Akt is a downstream target of NF-kappa B. *J. Biol. Chem.* **277**, 29674–29680 (2002). 12052823 10.1074/jbc.M112464200

[449] Hsiao, H. Y. et al. A critical role of astrocyte-mediated nuclear factor-kappaB-dependent inflammation in Huntington’s disease. *Hum. Mol. Genet.* **22**, 1826–1842 (2013). 23372043 10.1093/hmg/ddt036

[450] Anne, S. L., Saudou, F. & Humbert, S. Phosphorylation of huntingtin by cyclin-dependent kinase 5 is induced by DNA damage and regulates wild-type and mutant huntingtin toxicity in neurons. *J. Neurosci.* **27**, 7318–7328 (2007). 17611284 10.1523/JNEUROSCI.1831-07.2007 PMC6794597

[451] Kaminosono, S. et al. Suppression of mutant Huntingtin aggregate formation by Cdk5/p35 through the effect on microtubule stability. *J. Neurosci.* **28**, 8747–8755 (2008). 18753376 10.1523/JNEUROSCI.0973-08.2008 PMC6670830

[452] Muchowski, P. J., Ning, K., D’Souza-Schorey, C. & Fields, S. Requirement of an intact microtubule cytoskeleton for aggregation and inclusion body formation by a mutant huntingtin fragment. *Proc. Natl Acad. Sci. USA* **99**, 727–732 (2002). 11792857 10.1073/pnas.022628699 PMC117373

[453] Taylor, J. P. et al. Aggresomes protect cells by enhancing the degradation of toxic polyglutamine-containing protein. *Hum. Mol. Genet.* **12**, 749–757 (2003). 12651870 10.1093/hmg/ddg074

[454] Paoletti, P. et al. Dopaminergic and glutamatergic signaling crosstalk in Huntington’s disease neurodegeneration: the role of p25/cyclin-dependent kinase 5. *J. Neurosci.* **28**, 10090–10101 (2008). 18829967 10.1523/JNEUROSCI.3237-08.2008 PMC6671267

[455] Pulverer, B. J. et al. Phosphorylation of c-jun mediated by MAP kinases. *Nature* **353**, 670–674 (1991). 1922387 10.1038/353670a0

[456] Weston, C. R. & Davis, R. J. The JNK signal transduction pathway. *Curr. Opin. Cell Biol.* **19**, 142–149 (2007). 17303404 10.1016/j.ceb.2007.02.001

[457] Jebelli, J. D., Hooper, C., Garden, G. A. & Pocock, J. M. Emerging roles of p53 in glial cell function in health and disease. *Glia* **60**, 515–525 (2012). 22105777 10.1002/glia.22268 PMC4195591

[458] Junyent, F. et al. Gene expression profile in JNK3 null mice: a novel specific activation of the PI3K/AKT pathway. *J. Neurochem.* **117**, 244–252 (2011). 21255018 10.1111/j.1471-4159.2011.07195.x

[459] Song, G., Ouyang, G. & Bao, S. The activation of Akt/PKB signaling pathway and cell survival. *J. Cell Mol. Med.* **9**, 59–71 (2005). 15784165 10.1111/j.1582-4934.2005.tb00337.x PMC6741304

[460] Meriin, A. B. et al. Intracellular aggregation of polypeptides with expanded polyglutamine domain is stimulated by stress-activated kinase MEKK1. *J. Cell Biol.* **153**, 851–864 (2001). 11352944 10.1083/jcb.153.4.851 PMC2192371

[461] Liu, Y. F. Expression of polyglutamine-expanded Huntingtin activates the SEK1-JNK pathway and induces apoptosis in a hippocampal neuronal cell line. *J. Biol. Chem.* **273**, 28873–28877 (1998). 9786889 10.1074/jbc.273.44.28873

[462] Perrin, V. et al. Implication of the JNK pathway in a rat model of Huntington’s disease. *Exp. Neurol.* **215**, 191–200 (2009). 19022249 10.1016/j.expneurol.2008.10.008

[463] Guo, X. et al. ASK1 in neurodegeneration. *Adv. Biol. Regul.* **66**, 63–71 (2017). 28882588 10.1016/j.jbior.2017.08.003

[464] Nishitoh, H. et al. ASK1 is essential for endoplasmic reticulum stress-induced neuronal cell death triggered by expanded polyglutamine repeats. *Genes Dev.* **16**, 1345–1355 (2002). 12050113 10.1101/gad.992302 PMC186318

[465] Fan, J. et al. P38 MAPK is involved in enhanced NMDA receptor-dependent excitotoxicity in YAC transgenic mouse model of Huntington disease. *Neurobiol. Dis.* **45**, 999–1009 (2012). 22198502 10.1016/j.nbd.2011.12.019

[466] Huang, Z. N. et al. Inhibition of p38 mitogen-activated protein kinase ameliorates HAP40 depletion-induced toxicity and proteasomal defect in Huntington’s Disease model. *Mol. Neurobiol.* **58**, 2704–2723 (2021). 33492644 10.1007/s12035-020-02280-y

[467] Patterson, K. I., Brummer, T., O’Brien, P. M. & Daly, R. J. Dual-specificity phosphatases: critical regulators with diverse cellular targets. *Biochem. J.* **418**, 475–489 (2009). 19228121 10.1042/bj20082234

[468] Gladding, C. M. et al. Alterations in STriatal-enriched protein tyrosine phosphatase expression, activation, and downstream signaling in early and late stages of the YAC128 Huntington’s disease mouse model. *J. Neurochem.* **130**, 145–159 (2014). 24588402 10.1111/jnc.12700 PMC4065618

[469] Legos, J. J. et al. The selective p38 inhibitor SB-239063 protects primary neurons from mild to moderate excitotoxic injury. *Eur. J. Pharm.* **447**, 37–42 (2002). 10.1016/s0014-2999(02)01890-3 12106800

[470] Gines, S. et al. Enhanced Akt signaling is an early pro-survival response that reflects N-methyl-D-aspartate receptor activation in Huntington’s disease knock-in striatal cells. *J. Biol. Chem.* **278**, 50514–50522 (2003). 14522959 10.1074/jbc.M309348200

[471] Warby, S. C. et al. Huntingtin phosphorylation on serine 421 is significantly reduced in the striatum and by polyglutamine expansion in vivo. *Hum. Mol. Genet.* **14**, 1569–1577 (2005). 15843398 10.1093/hmg/ddi165

[472] Bryan, M. R. et al. Manganese acts upon Insulin/IGF receptors to phosphorylate AKT and increase glucose uptake in Huntington’s disease cells. *Mol. Neurobiol.* **57**, 1570–1593 (2020). 31797328 10.1007/s12035-019-01824-1 PMC7062569

[473] Ribeiro, M. et al. Insulin and IGF-1 improve mitochondrial function in a PI-3K/Akt-dependent manner and reduce mitochondrial generation of reactive oxygen species in Huntington’s disease knock-in striatal cells. *Free Radic. Biol. Med.* **74**, 129–144 (2014). 24992836 10.1016/j.freeradbiomed.2014.06.023

[474] Senousy, M. A., Hanafy, M. E., Shehata, N. & Rizk, S. M. Erythropoietin and Bacillus calmette-guerin vaccination mitigate 3-nitropropionic acid-induced Huntington-like disease in rats by modulating the PI3K/Akt/mTOR/P70S6K pathway and enhancing the autophagy. *ACS Chem. Neurosci.* **13**, 721–732 (2022). 35226456 10.1021/acschemneuro.1c00523

[475] Li, L. et al. Mutant Huntingtin impairs pancreatic beta-cells by recruiting IRS-2 and disturbing the PI3K/AKT/FoxO1 signaling pathway in Huntington’s disease. *J. Mol. Neurosci.* **71**, 2646–2658 (2021). 34331233 10.1007/s12031-021-01869-9

[476] Somvanshi, R. K., Jhajj, A., Heer, M. & Kumar, U. Characterization of somatostatin receptors and associated signaling pathways in pancreas of R6/2 transgenic mice. *Biochim. Biophys. Acta Mol. Basis Dis.* **1864**, 359–373 (2018). 29104117 10.1016/j.bbadis.2017.11.002

[477] Goutman, S. A. et al. Emerging insights into the complex genetics and pathophysiology of amyotrophic lateral sclerosis. *Lancet Neurol.* **21**, 465–479 (2022). 35334234 10.1016/S1474-4422(21)00414-2 PMC9513754

[478] Chia, R., Chio, A. & Traynor, B. J. Novel genes associated with amyotrophic lateral sclerosis: diagnostic and clinical implications. *Lancet Neurol.* **17**, 94–102 (2018). 29154141 10.1016/S1474-4422(17)30401-5 PMC5901717

[479] Renton, A. E., Chio, A. & Traynor, B. J. State of play in amyotrophic lateral sclerosis genetics. *Nat. Neurosci.* **17**, 17–23 (2014). 24369373 10.1038/nn.3584 PMC4544832

[480] Zarei, S. et al. A comprehensive review of amyotrophic lateral sclerosis. *Surg. Neurol. Int.* **6**, 171 (2015). 26629397 10.4103/2152-7806.169561 PMC4653353

[481] Renton, A. E., Chiò, A. & Traynor, B. J. State of play in amyotrophic lateral sclerosis genetics. *Nat. Neurosci.* **17**, 17–23 (2014). 24369373 10.1038/nn.3584 PMC4544832

[482] Johnson, B. S. et al. TDP-43 is intrinsically aggregation-prone, and amyotrophic lateral sclerosis-linked mutations accelerate aggregation and increase toxicity. *J. Biol. Chem.* **284**, 20329–20339 (2009). 19465477 10.1074/jbc.M109.010264 PMC2740458

[483] Peters, O. M., Ghasemi, M. & Brown, R. H. Jr Emerging mechanisms of molecular pathology in ALS. *J. Clin. Investig.* **125**, 2548 (2015). 26030230 10.1172/JCI82693 PMC4518693

[484] Neumann, M. et al. Ubiquitinated TDP-43 in frontotemporal lobar degeneration and amyotrophic lateral sclerosis. *Science* **314**, 130–133 (2006). 17023659 10.1126/science.1134108

[485] Cohen, T. J. et al. An acetylation switch controls TDP-43 function and aggregation propensity. *Nat. Commun.* **6**, 5845 (2015). 25556531 10.1038/ncomms6845 PMC4407365

[486] Hasegawa, M. et al. Phosphorylated TDP-43 in frontotemporal lobar degeneration and amyotrophic lateral sclerosis. *Ann. Neurol.* **64**, 60–70 (2008). 18546284 10.1002/ana.21425 PMC2674108

[487] Taylor, L. M., McMillan, P. J., Kraemer, B. C. & Liachko, N. F. Tau tubulin kinases in proteinopathy. *FEBS J.* **286**, 2434–2446 (2019). 31034749 10.1111/febs.14866 PMC6936727

[488] Wilson, R. S. et al. TDP-43 pathology, cognitive decline, and dementia in old age. *JAMA Neurol.* **70**, 1418–1424 (2013). 24080705 10.1001/jamaneurol.2013.3961 PMC3830649

[489] Lee, E. B., Lee, V. M. & Trojanowski, J. Q. Gains or losses: molecular mechanisms of TDP43-mediated neurodegeneration. *Nat. Rev. Neurosci.* **13**, 38–50 (2011). 22127299 10.1038/nrn3121 PMC3285250

[490] Deng, H., Gao, K. & Jankovic, J. The role of FUS gene variants in neurodegenerative diseases. *Nat. Rev. Neurol.* **10**, 337–348 (2014). 24840975 10.1038/nrneurol.2014.78

[491] Kim, G. et al. ALS genetics: gains, losses, and implications for future therapies. *Neuron* **108**, 822–842 (2020). 32931756 10.1016/j.neuron.2020.08.022 PMC7736125

[492] Mitchell, J. C. et al. Overexpression of human wild-type FUS causes progressive motor neuron degeneration in an age- and dose-dependent fashion. *Acta Neuropathol.* **125**, 273–288 (2013). 22961620 10.1007/s00401-012-1043-z PMC3549237

[493] Sharma, A. et al. ALS-associated mutant FUS induces selective motor neuron degeneration through toxic gain of function. *Nat. Commun.* **7**, 10465 (2016). 26842965 10.1038/ncomms10465 PMC4742863

[494] Shiihashi, G. et al. Mislocated FUS is sufficient for gain-of-toxic-function amyotrophic lateral sclerosis phenotypes in mice. *Brain* **139**, 2380–2394 (2016). 27368346 10.1093/brain/aww161

[495] Lagier-Tourenne, C. et al. Divergent roles of ALS-linked proteins FUS/TLS and TDP-43 intersect in processing long pre-mRNAs. *Nat. Neurosci.* **15**, 1488–1497 (2012). 23023293 10.1038/nn.3230 PMC3586380

[496] Zou, Z. Y. et al. Genetic epidemiology of amyotrophic lateral sclerosis: a systematic review and meta-analysis. *J. Neurol. Neurosurg. Psychiatry* **88**, 540–549 (2017). 28057713 10.1136/jnnp-2016-315018

[497] Lindberg, M. J. et al. Systematically perturbed folding patterns of amyotrophic lateral sclerosis (ALS)-associated SOD1 mutants. *Proc. Natl. Acad. Sci. USA* **102**, 9754–9759 (2005). 15987780 10.1073/pnas.0501957102 PMC1174986

[498] Shi, P. et al. Mitochondrial dysfunction in amyotrophic lateral sclerosis. *Biochim. Biophys. Acta* **1802**, 45–51 (2010). 19715760 10.1016/j.bbadis.2009.08.012 PMC2790551

[499] Ratovitski, T. et al. Variation in the biochemical/biophysical properties of mutant superoxide dismutase 1 enzymes and the rate of disease progression in familial amyotrophic lateral sclerosis kindreds. *Hum. Mol. Genet.* **8**, 1451–1460 (1999). 10400992 10.1093/hmg/8.8.1451

[500] Reaume, A. G. et al. Motor neurons in Cu/Zn superoxide dismutase-deficient mice develop normally but exhibit enhanced cell death after axonal injury. *Nat. Genet.* **13**, 43–47 (1996). 8673102 10.1038/ng0596-43

[501] Saccon, R. A., Bunton-Stasyshyn, R. K., Fisher, E. M. & Fratta, P. Is SOD1 loss of function involved in amyotrophic lateral sclerosis? *Brain* **136**, 2342–2358 (2013). 23687121 10.1093/brain/awt097 PMC3722346

[502] Wang, T. et al. C9orf72 regulates energy homeostasis by stabilizing mitochondrial complex I assembly. *Cell Metab.* **33**, 531–546.e539 (2021). 33545050 10.1016/j.cmet.2021.01.005 PMC8579819

[503] Sakae, N. et al. Poly-GR dipeptide repeat polymers correlate with neurodegeneration and clinicopathological subtypes in C9ORF72-related brain disease. *Acta Neuropathol. Commun.* **6**, 63 (2018). 30029693 10.1186/s40478-018-0564-7 PMC6054740

[504] Renton, A. E. et al. A hexanucleotide repeat expansion in C9ORF72 is the cause of chromosome 9p21-linked ALS-FTD. *Neuron* **72**, 257–268 (2011). 21944779 10.1016/j.neuron.2011.09.010 PMC3200438

[505] DeJesus-Hernandez, M. et al. Expanded GGGGCC hexanucleotide repeat in noncoding region of C9ORF72 causes chromosome 9p-linked FTD and ALS. *Neuron* **72**, 245–256 (2011). 21944778 10.1016/j.neuron.2011.09.011 PMC3202986

[506] Beckers, J., Tharkeshwar, A. K. & Van Damme, P. C9orf72 ALS-FTD: recent evidence for dysregulation of the autophagy-lysosome pathway at multiple levels. *Autophagy* **17**, 3306–3322 (2021). 33632058 10.1080/15548627.2021.1872189 PMC8632097

[507] Mejzini, R. et al. ALS genetics, mechanisms, and therapeutics: where are we now? *Front. Neurosci.* **13**, 1310 (2019). 31866818 10.3389/fnins.2019.01310 PMC6909825

[508] Cirulli, E. T. et al. Exome sequencing in amyotrophic lateral sclerosis identifies risk genes and pathways. *Science* **347**, 1436–1441 (2015). 25700176 10.1126/science.aaa3650 PMC4437632

[509] Freischmidt, A. et al. Haploinsufficiency of TBK1 causes familial ALS and fronto-temporal dementia. *Nat. Neurosci.* **18**, 631–636 (2015). 25803835 10.1038/nn.4000

[510] Williams, K. L. et al. Novel TBK1 truncating mutation in a familial amyotrophic lateral sclerosis patient of Chinese origin. *Neurobiol. Aging* **36**, 3334 e3331–3334 e3335 (2015). 10.1016/j.neurobiolaging.2015.08.013 26350399

[511] Tsai, P. C. et al. Mutational analysis of TBK1 in Taiwanese patients with amyotrophic lateral sclerosis. *Neurobiol. Aging* **40**, 191.e111–116 (2016). 10.1016/j.neurobiolaging.2015.12.022 26804609

[512] Oakes, J. A., Davies, M. C. & Collins, M. O. TBK1: a new player in ALS linking autophagy and neuroinflammation. *Mol. Brain* **10**, 5 (2017). 28148298 10.1186/s13041-017-0287-x PMC5288885

[513] Freischmidt, A. et al. Association of mutations in TBK1 with sporadic and familial amyotrophic lateral sclerosis and frontotemporal dementia. *JAMA Neurol.* **74**, 110–113 (2017). 27892983 10.1001/jamaneurol.2016.3712

[514] Larabi, A. et al. Crystal structure and mechanism of activation of TANK-binding kinase 1. *Cell Rep.* **3**, 734–746 (2013). 23453971 10.1016/j.celrep.2013.01.034

[515] Goncalves, A. et al. Functional dissection of the TBK1 molecular network. *PLoS One* **6**, e23971 (2011). 21931631 10.1371/journal.pone.0023971 PMC3169550

[516] Herhaus, L. et al. TBK1-mediated phosphorylation of LC3C and GABARAP-L2 controls autophagosome shedding by ATG4 protease. *EMBO Rep.* **21**, e48317 (2020). 31709703 10.15252/embr.201948317 PMC6945063

[517] Sullivan, P. M. et al. The ALS/FTLD-associated protein C9orf72 associates with SMCR8 and WDR41 to regulate the autophagy-lysosome pathway. *Acta Neuropathol. Commun.* **4**, 51 (2016). 27193190 10.1186/s40478-016-0324-5 PMC4870812

[518] Matsumoto, G., Shimogori, T., Hattori, N. & Nukina, N. TBK1 controls autophagosomal engulfment of polyubiquitinated mitochondria through p62/SQSTM1 phosphorylation. *Hum. Mol. Genet.* **24**, 4429–4442 (2015). 25972374 10.1093/hmg/ddv179

[519] Moore, A. S. & Holzbaur, E. L. Dynamic recruitment and activation of ALS-associated TBK1 with its target optineurin are required for efficient mitophagy. *Proc. Natl. Acad. Sci. USA* **113**, E3349–E3358 (2016). 27247382 10.1073/pnas.1523810113 PMC4914160

[520] Brenner, D. et al. Heterozygous Tbk1 loss has opposing effects in early and late stages of ALS in mice. *J. Exp. Med.* **216**, 267–278 (2019). 30635357 10.1084/jem.20180729 PMC6363427

[521] Brenner, D. et al. NEK1 mutations in familial amyotrophic lateral sclerosis. *Brain* **139**, e28 (2016). 26945885 10.1093/brain/aww033

[522] Kenna, K. P. et al. NEK1 variants confer susceptibility to amyotrophic lateral sclerosis. *Nat. Genet.* **48**, 1037 (2016). 27455347 10.1038/ng.3626 PMC5560030

[523] Nguyen, H. P. et al. NEK1 genetic variability in a Belgian cohort of ALS and ALS-FTD patients. *Neurobiol. Aging* **61**, e1–255.e7 (2018). 10.1016/j.neurobiolaging.2017.08.021 28935222

[524] Riva, N. et al. NEK1 Variants in a cohort of Italian patients with amyotrophic lateral sclerosis. *Front. Neurosci*. **16**, 833051 (2022). 10.3389/fnins.2022.833051 PMC9048593 35495032

[525] Higelin, J. et al. NEK1 loss-of-function mutation induces DNA damage accumulation in ALS patient-derived motoneurons. *Stem Cell Res* **30**, 150–162 (2018). 29929116 10.1016/j.scr.2018.06.005

[526] Wang, H. B. et al. NEK1-mediated retromer trafficking promotes blood-brain barrier integrity by regulating glucose metabolism and RIPK1 activation. *Nat Commun*. **12**, 4826 (2021). 10.1038/s41467-021-25157-7 PMC8355301 34376696

[527] Asghari Adib, E., Smithson, L. J. & Collins, C. A. An axonal stress response pathway: degenerative and regenerative signaling by DLK. *Curr. Opin. Neurobiol.* **53**, 110–119 (2018). 30053694 10.1016/j.conb.2018.07.002 PMC6536440

[528] Ghosh, A. S. et al. DLK induces developmental neuronal degeneration via selective regulation of proapoptotic JNK activity. *J. Cell Biol.* **194**, 751–764 (2011). 21893599 10.1083/jcb.201103153 PMC3171129

[529] Xiong, H. et al. Neural circuit changes in neurological disorders: evidence from in vivo two-photon imaging. *Ageing Res Rev.* **87**, 101933 (2023). 37061201 10.1016/j.arr.2023.101933

[530] Le Pichon, C. E. et al. Loss of dual leucine zipper kinase signaling is protective in animal models of neurodegenerative disease. *Sci. Transl. Med*. **9**, eaag0394 (2017). 10.1126/scitranslmed.aag0394 28814543

[531] Wlaschin, J. J. et al. Promoting regeneration while blocking cell death preserves motor neuron function in a model of ALS. *Brain* **146**, 2016–2028 (2022). 10.1093/brain/awac415 PMC10411937 36342754

[532] Xu, D. et al. TBK1 suppresses RIPK1-driven apoptosis and inflammation during development and in aging. *Cell* **174**, 1477–1491.e1419 (2018). 30146158 10.1016/j.cell.2018.07.041 PMC6128749

[533] Geng, J. et al. Regulation of RIPK1 activation by TAK1-mediated phosphorylation dictates apoptosis and necroptosis. *Nat. Commun.* **8**, 359 (2017). 28842570 10.1038/s41467-017-00406-w PMC5572456

[534] Wang, H. et al. NEK1-mediated retromer trafficking promotes blood-brain barrier integrity by regulating glucose metabolism and RIPK1 activation. *Nat. Commun.* **12**, 4826 (2021). 34376696 10.1038/s41467-021-25157-7 PMC8355301

[535] Du, B. et al. Iron promotes both ferroptosis and necroptosis in the early stage of reperfusion in ischemic stroke. *Genes Dis.* **11**, 101262 (2024). 39286656 10.1016/j.gendis.2024.101262 PMC11402992

[536] Li, W. et al. Nuclear RIPK1 promotes chromatin remodeling to mediate inflammatory response. *Cell Res.* **32**, 621–637 (2022). 35661830 10.1038/s41422-022-00673-3 PMC9253060

[537] Mifflin, L., Ofengeim, D. & Yuan, J. Receptor-interacting protein kinase 1 (RIPK1) as a therapeutic target. *Nat. Rev. Drug Discov.* **19**, 553–571 (2020). 32669658 10.1038/s41573-020-0071-y PMC7362612

[538] Liu, Y. J., Lee, L. M., Lai, H. L. & Chern, Y. Aberrant activation of AMP-activated protein kinase contributes to the abnormal distribution of HuR in amyotrophic lateral sclerosis. *FEBS Lett.* **589**, 432–439 (2015). 25592834 10.1016/j.febslet.2014.12.029

[539] Scarmeas, N. et al. Premorbid weight, body mass, and varsity athletics in ALS. *Neurology* **59**, 773–775 (2002). 12221178 10.1212/wnl.59.5.773

[540] Browne, S. E. et al. Bioenergetic abnormalities in discrete cerebral motor pathways presage spinal cord pathology in the G93A SOD1 mouse model of ALS. *Neurobiol. Dis.* **22**, 599–610 (2006). 16616851 10.1016/j.nbd.2006.01.001

[541] Sui, Y. et al. Adenosine monophosphate-activated protein kinase activation enhances embryonic neural stem cell apoptosis in a mouse model of amyotrophic lateral sclerosis. *Neural Regen. Res.* **9**, 1770–1778 (2014). 25422638 10.4103/1673-5374.143421 PMC4238165

[542] Lim, M. A. et al. Reduced activity of AMP-activated protein kinase protects against genetic models of motor neuron disease. *J. Neurosci.* **32**, 1123–1141 (2012). 22262909 10.1523/JNEUROSCI.6554-10.2012 PMC3742882

[543] Liu, Y. J. et al. Activation of AMP-activated protein kinase alpha1 mediates mislocalization of TDP-43 in amyotrophic lateral sclerosis. *Hum. Mol. Genet.* **24**, 787–801 (2015). 25256353 10.1093/hmg/ddu497

[544] Coughlan, K. S., Mitchem, M. R., Hogg, M. C. & Prehn, J. H. Preconditioning” with latrepirdine, an adenosine 5’-monophosphate-activated protein kinase activator, delays amyotrophic lateral sclerosis progression in SOD1(G93A) mice. *Neurobiol. Aging* **36**, 1140–1150 (2015). 25443289 10.1016/j.neurobiolaging.2014.09.022

[545] Warita, H. et al. Early decrease of survival signal-related proteins in spinal motor neurons of presymptomatic transgenic mice with a mutant SOD1 gene. *Apoptosis* **6**, 345–352 (2001). 11483858 10.1023/a:1011334018804

[546] Manning, B. D. & Toker, A. AKT/PKB signaling: navigating the network. *Cell* **169**, 381–405 (2017). 28431241 10.1016/j.cell.2017.04.001 PMC5546324

[547] Hu, J. H. et al. Protein kinase and protein phosphatase expression in amyotrophic lateral sclerosis spinal cord. *J. Neurochem.* **85**, 432–442 (2003). 12675919 10.1046/j.1471-4159.2003.01670.x

[548] Yang, W., Leystra-Lantz, C. & Strong, M. J. Upregulation of GSK3beta expression in frontal and temporal cortex in ALS with cognitive impairment (ALSci). *Brain Res.* **1196**, 131–139 (2008). 18221734 10.1016/j.brainres.2007.12.031

[549] Choi, H. J. et al. Recent advances on the role of GSK3beta in the pathogenesis of amyotrophic lateral sclerosis. *Brain Sci*. **10**, 675 (2020). 10.3390/brainsci10100675 PMC7600609 32993098

[550] Sreedharan, J., Neukomm, L. J., Brown, R. H. Jr. & Freeman, M. R. Age-dependent TDP-43-mediated motor neuron degeneration requires GSK3, hat-trick, and xmas-2. *Curr. Biol.* **25**, 2130–2136 (2015). 26234214 10.1016/j.cub.2015.06.045 PMC4546534

[551] Yang, Y. M. et al. A small molecule screen in stem-cell-derived motor neurons identifies a kinase inhibitor as a candidate therapeutic for ALS. *Cell Stem Cell* **12**, 713–726 (2013). 23602540 10.1016/j.stem.2013.04.003 PMC3707511

[552] Nakagawa, O. et al. ROCK-I and ROCK-II, two isoforms of Rho-associated coiled-coil forming protein serine/threonine kinase in mice. *FEBS Lett.* **392**, 189–193 (1996). 8772201 10.1016/0014-5793(96)00811-3

[553] Jacobs, M. et al. The structure of dimeric ROCK I reveals the mechanism for ligand selectivity. *J. Biol. Chem.* **281**, 260–268 (2006). 16249185 10.1074/jbc.M508847200

[554] Iizuka, M. et al. Distinct distribution and localization of Rho-kinase in mouse epithelial, muscle and neural tissues. *Cell Struct. Funct.* **37**, 155–175 (2012). 22986902 10.1247/csf.12018

[555] Conti, A. et al. Increased expression of Myosin binding protein H in the skeletal muscle of amyotrophic lateral sclerosis patients. *Biochim Biophys. Acta* **1842**, 99–106 (2014). 24184715 10.1016/j.bbadis.2013.10.013

[556] Takata, M. et al. Fasudil, a rho kinase inhibitor, limits motor neuron loss in experimental models of amyotrophic lateral sclerosis. *Br. J. Pharm.* **170**, 341–351 (2013). 10.1111/bph.12277 PMC3834758 23763343

[557] Tonges, L. et al. Rho kinase inhibition modulates microglia activation and improves survival in a model of amyotrophic lateral sclerosis. *Glia* **62**, 217–232 (2014). 24311453 10.1002/glia.22601

[558] Preyer, M., Shu, C. W. & Wang, J. Y. J. Delayed activation of Bax by DNA damage in embryonic stem cells with knock-in mutations of the AbI nuclear localization signals. *Cell Death Differ.* **14**, 1139–1148 (2007). 17363963 10.1038/sj.cdd.4402119

[559] Gutierrez, D. A. et al. c-Abl kinase at the crossroads of healthy synaptic remodeling and synaptic dysfunction in neurodegenerative diseases. *Neural Regen. Res.* **18**, 237–243 (2023). 35900397 10.4103/1673-5374.346540 PMC9396477

[560] Schlatterer, S. D., Acker, C. M. & Davies, P. c-Abl in neurodegenerative disease. *J. Mol. Neurosci.* **45**, 445–452 (2011). 21728062 10.1007/s12031-011-9588-1 PMC3329755

[561] Katsumata, R. et al. c-Abl inhibition delays motor neuron degeneration in the G93A mouse, an animal model of amyotrophic lateral sclerosis. *PLoS One* **7**, e46185 (2012). 23049975 10.1371/journal.pone.0046185 PMC3458026

[562] Imamura, K. et al. The Src/c-Abl pathway is a potential therapeutic target in amyotrophic lateral sclerosis. *Sci. Transl. Med*. **9**, eaaf3962 (2017). 10.1126/scitranslmed.aaf3962 28539470

[563] Lee, S. B. et al. c-Abl regulates the pathological deposition of TDP-43 via tyrosine 43 phosphorylation. *Cells*. **11**, 3972 (2022). 10.3390/cells11243972 PMC9776721 36552734

[564] Motaln, H. et al. Abl kinase-mediated FUS Tyr526 phosphorylation alters nucleocytoplasmic FUS localization in FTLD-FUS. *Brain* **146**, 4088-4104 (2023). 10.1093/brain/awad130 PMC10545532 37071594

[565] Iguchi, Y. et al. IkappaB kinase phosphorylates cytoplasmic TDP-43 and promotes its proteasome degradation. *J. Cell Biol*. **223**, e202302048 (2024). 10.1083/jcb.202302048 PMC10783433 38197897

[566] Ho, D. M. et al. cAMP/PKA signaling regulates TDP-43 aggregation and mislocalization. *Proc. Natl. Acad. Sci. USA* **121**, e2400732121 (2024). 38838021 10.1073/pnas.2400732121 PMC11181030

[567] Kametani, F. et al. Identification of casein kinase-1 phosphorylation sites on TDP-43. *Biochem. Biophys. Res. Commun.* **382**, 405–409 (2009). 19285963 10.1016/j.bbrc.2009.03.038

[568] Liachko, N. F. et al. CDC7 inhibition blocks pathological TDP-43 phosphorylation and neurodegeneration. *Ann. Neurol.* **74**, 39–52 (2013). 23424178 10.1002/ana.23870 PMC3775949

[569] Taylor, L. M. et al. Pathological phosphorylation of tau and TDP-43 by TTBK1 and TTBK2 drives neurodegeneration. *Mol. Neurodegener.* **13**, 7 (2018). 29409526 10.1186/s13024-018-0237-9 PMC5802059

[570] Liachko, N. F. et al. The tau tubulin kinases TTBK1/2 promote accumulation of pathological TDP-43. *PLoS Genet* **10**, e1004803 (2014). 25473830 10.1371/journal.pgen.1004803 PMC4256087

[571] Pilo, C. A. et al. Mutations in protein kinase C gamma promote spinocerebellar ataxia type 14 by impairing kinase autoinhibition. *Sci. Signal* **15**, eabk1147 (2022). 36166510 10.1126/scisignal.abk1147 PMC9810342

[572] Klebe, S. et al. New mutations in protein kinase C gamma associated with spinocerebellar ataxia type 14. *Ann. Neurol.* **58**, 720–729 (2005). 16193476 10.1002/ana.20628

[573] Lin, C. W., Fan, C. H., Chang, Y. C. & Hsieh-Li, H. M. ERK activation precedes Purkinje cell loss in mice with Spinocerebellar ataxia type 17. *Neurosci. Lett.* **738**, 135337 (2020). 32877710 10.1016/j.neulet.2020.135337

[574] Goetz, S. C., Liem, K. F. Jr. & Anderson, K. V. The spinocerebellar ataxia-associated gene Tau tubulin kinase 2 controls the initiation of ciliogenesis. *Cell* **151**, 847–858 (2012). 23141541 10.1016/j.cell.2012.10.010 PMC3496184

[575] Glover, R. T. et al. Interaction of the N-methyl-D-aspartic acid receptor NR2D subunit with the c-Abl tyrosine kinase. *J. Biol. Chem.* **275**, 12725–12729 (2000). 10777567 10.1074/jbc.275.17.12725

[576] Smith, D. H., Johnson, V. E., Trojanowski, J. Q. & Stewart, W. Chronic traumatic encephalopathy—confusion and controversies. *Nat. Rev. Neurol.* **15**, 179–183 (2019). 30664683 10.1038/s41582-018-0114-8 PMC6532781

[577] McKee, A. C. et al. Chronic traumatic encephalopathy (CTE): criteria for neuropathological diagnosis and relationship to repetitive head impacts. *Acta Neuropathol.* **145**, 371–394 (2023). 36759368 10.1007/s00401-023-02540-w PMC10020327

[578] Kim, N. et al. Inhibition of death-associated protein kinase 1 attenuates cis P-tau and neurodegeneration in traumatic brain injury. *Prog. Neurobiol.* **203**, 102072 (2021). 33979671 10.1016/j.pneurobio.2021.102072 PMC8217320

[579] Braun, N. J., Yao, K. R., Alford, P. W. & Liao, D. Mechanical injuries of neurons induce tau mislocalization to dendritic spines and tau-dependent synaptic dysfunction. *Proc. Natl. Acad. Sci. USA* **117**, 29069–29079 (2020). 33139536 10.1073/pnas.2008306117 PMC7682580

[580] Meng, S. et al. ASK1-K716R reduces neuroinflammation and white matter injury via preserving blood-brain barrier integrity after traumatic brain injury. *J. Neuroinflamm.* **20**, 244 (2023). 10.1186/s12974-023-02923-6 PMC10594934 37875988

[581] Oliva, A. A. Jr. et al. STAT3 signaling after traumatic brain injury. *J. Neurochem.* **120**, 710–720 (2012). 22145815 10.1111/j.1471-4159.2011.07610.x

[582] Ding, X. et al. DNALI1 promotes neurodegeneration after traumatic brain injury via inhibition of autophagosome-lysosome fusion. *Adv. Sci.* **11**, e2306399 (2024). 10.1002/advs.202306399 PMC11022701 38348540

[583] Roy, S. M. et al. Targeting human central nervous system protein kinases: an isoform selective p38alphaMAPK inhibitor that attenuates disease progression in Alzheimer’s disease mouse models. *ACS Chem. Neurosci.* **6**, 666–680 (2015). 25676389 10.1021/acschemneuro.5b00002 PMC4404319

[584] Zhou, Z. et al. Retention of normal glia function by an isoform-selective protein kinase inhibitor drug candidate that modulates cytokine production and cognitive outcomes. *J. Neuroinflamm.* **14**, 75 (2017). 10.1186/s12974-017-0845-2 PMC5382362 28381303

[585] Rutigliano, G. et al. An isoform-selective p38alpha mitogen-activated protein kinase inhibitor rescues early entorhinal cortex dysfunctions in a mouse model of Alzheimer’s disease. *Neurobiol. Aging* **70**, 86–91 (2018). 30007168 10.1016/j.neurobiolaging.2018.06.006 PMC6119125

[586] Duffy, J. P. et al. The discovery of VX-745: a novel and selective p38alpha kinase inhibitor. *ACS Med. Chem. Lett.* **2**, 758–763 (2011). 24900264 10.1021/ml2001455 PMC4018046

[587] Alam, J., Blackburn, K. & Patrick, D. Neflamapimod: clinical phase 2b-ready oral small molecule inhibitor of p38alpha to reverse synaptic dysfunction in early Alzheimer’s Disease. *J. Prev. Alzheimers Dis.* **4**, 273–278 (2017). 29181493 10.14283/jpad.2017.41

[588] Scheltens, P. et al. An exploratory clinical study of p38alpha kinase inhibition in Alzheimer’s disease. *Ann. Clin. Transl. Neurol.* **5**, 464–473 (2018). 29687023 10.1002/acn3.549 PMC5899915

[589] Prins, N. D. et al. A phase 2 double-blind placebo-controlled 24-week treatment clinical study of the p38 alpha kinase inhibitor neflamapimod in mild Alzheimer’s disease. *Alzheimers Res. Ther.* **13**, 106 (2021). 34044875 10.1186/s13195-021-00843-2 PMC8157623

[590] Martinez, A. et al. First non-ATP competitive glycogen synthase kinase 3 beta (GSK-3beta) inhibitors: thiadiazolidinones (TDZD) as potential drugs for the treatment of Alzheimer’s disease. *J. Med. Chem.* **45**, 1292–1299 (2002). 11881998 10.1021/jm011020u

[591] Martinez, A., Castro, A., Dorronsoro, I. & Alonso, M. Glycogen synthase kinase 3 (GSK-3) inhibitors as new promising drugs for diabetes, neurodegeneration, cancer, and inflammation. *Med. Res. Rev.* **22**, 373–384 (2002). 12111750 10.1002/med.10011

[592] Luna-Medina, R. et al. NP031112, a thiadiazolidinone compound, prevents inflammation and neurodegeneration under excitotoxic conditions: potential therapeutic role in brain disorders. *J. Neurosci.* **27**, 5766–5776 (2007). 17522320 10.1523/JNEUROSCI.1004-07.2007 PMC6672766

[593] del Ser, T. et al. Treatment of Alzheimer’s disease with the GSK-3 inhibitor tideglusib: a pilot study. *J. Alzheimers Dis.* **33**, 205–215 (2013). 22936007 10.3233/JAD-2012-120805

[594] Lovestone, S. et al. A phase II trial of tideglusib in Alzheimer’s disease. *J. Alzheimers Dis.* **45**, 75–88 (2015). 25537011 10.3233/JAD-141959

[595] Cortes-Gomez, M. A. et al. Tau phosphorylation by glycogen synthase kinase 3beta modulates enzyme acetylcholinesterase expression. *J. Neurochem.* **157**, 2091–2105 (2021). 32955735 10.1111/jnc.15189 PMC8359467

[596] Su, Y. et al. Lithium, a common drug for bipolar disorder treatment, regulates amyloid-beta precursor protein processing. *Biochemistry* **43**, 6899–6908 (2004). 15170327 10.1021/bi035627j

[597] Du, B., Chen, K., Wang, W. & Lei, P. Targeting metals in Alzheimer’s disease: an update. *J. Alzheimers Dis.* **101**, S141–S154 (2024). 39422951 10.3233/JAD-240140

[598] Leroy, K. et al. Lithium treatment arrests the development of neurofibrillary tangles in mutant tau transgenic mice with advanced neurofibrillary pathology. *J. Alzheimers Dis.* **19**, 705–719 (2010). 20110614 10.3233/JAD-2010-1276

[599] Alvarez-Ruiz, Y. & Carrillo-Mora, P. Amyloid beta 25-35 impairs reconsolidation of object recognition memory in rats and this effect is prevented by lithium carbonate. *Neurosci. Lett.* **548**, 79–83 (2013). 23774478 10.1016/j.neulet.2013.06.003

[600] Cardillo, G. M. et al. Chronic lithium treatment increases telomere length in parietal cortex and hippocampus of triple-transgenic Alzheimer’s disease mice. *J. Alzheimers Dis.* **63**, 93–101 (2018). 29614649 10.3233/JAD-170838

[601] Habib, A. et al. Comparing the effect of the novel ionic cocrystal of lithium salicylate proline (LISPRO) with lithium carbonate and lithium salicylate on memory and behavior in female APPswe/PS1dE9 Alzheimer’s mice. *J. Neurosci. Res.* **97**, 1066–1080 (2019). 31102295 10.1002/jnr.24438 PMC6625860

[602] Yeh, H. L. & Tsai, S. J. Lithium may be useful in the prevention of Alzheimer’s disease in individuals at risk of presenile familial Alzheimer’s disease. *Med. Hypotheses* **71**, 948–951 (2008). 18760542 10.1016/j.mehy.2008.03.049

[603] Forlenza, O. V. et al. Long-term lithium treatment reduces glucose metabolism in the cerebellum and hippocampus of nondemented older adults: an [(1)(8)F]FDG-PET study. *ACS Chem. Neurosci.* **5**, 484–489 (2014). 24730717 10.1021/cn5000315 PMC4063507

[604] Hampel, H. et al. Lithium trial in Alzheimer’s disease: a randomized, single-blind, placebo-controlled, multicenter 10-week study. *J. Clin. Psychiatry* **70**, 922–931 (2009). 19573486

[605] Feyt, C. et al. Lithium chloride increases the production of amyloid-beta peptide independently from its inhibition of glycogen synthase kinase 3. *J. Biol. Chem.* **280**, 33220–33227 (2005). 16014628 10.1074/jbc.M501610200

[606] Lei, P., Ayton, S., Bush, A. I. & Adlard, P. A. GSK-3 in neurodegenerative diseases. *Int. J. Alzheimers Dis.* **2011**, 189246 (2011). 21629738 10.4061/2011/189246 PMC3100544

[607] Nakashima, H. et al. Chronic lithium treatment decreases tau lesions by promoting ubiquitination in a mouse model of tauopathies. *Acta Neuropathol.* **110**, 547–556 (2005). 16228182 10.1007/s00401-005-1087-4

[608] Lei, P. et al. Lithium suppression of tau induces brain iron accumulation and neurodegeneration. *Mol. Psychiatr.* **22**, 396–406 (2017). 10.1038/mp.2016.96 27400857

[609] Folch, J. et al. Masitinib for the treatment of mild to moderate Alzheimer’s disease. *Expert Rev. Neurother.* **15**, 587–596 (2015). 25961655 10.1586/14737175.2015.1045419

[610] Lonskaya, I. et al. Nilotinib and bosutinib modulate pre-plaque alterations of blood immune markers and neuro-inflammation in Alzheimer’s disease models. *Neuroscience* **304**, 316–327 (2015). 26235435 10.1016/j.neuroscience.2015.07.070

[611] Piette, F. et al. Masitinib as an adjunct therapy for mild-to-moderate Alzheimer’s disease: a randomised, placebo-controlled phase 2 trial. *Alzheimers Res. Ther.* **3**, 16 (2011). 21504563 10.1186/alzrt75 PMC3226277

[612] Dubois, B. et al. Masitinib for mild-to-moderate Alzheimer’s disease: results from a randomized, placebo-controlled, phase 3, clinical trial. *Alzheimers Res. Ther.* **15**, 39 (2023). 36849969 10.1186/s13195-023-01169-x PMC9972756

[613] Turner, R. S. et al. Nilotinib effects on safety, tolerability, and biomarkers in Alzheimer’s disease. *Ann. Neurol.* **88**, 183–194 (2020). 32468646 10.1002/ana.25775 PMC7383852

[614] Tang, S. J. et al. Fyn kinase inhibition reduces protein aggregation, increases synapse density and improves memory in transgenic and traumatic Tauopathy. *Acta Neuropathol. Commun.* **8**, 96 (2020). 32611392 10.1186/s40478-020-00976-9 PMC7329553

[615] Dinda, B. et al. Therapeutic potentials of plant iridoids in Alzheimer’s and Parkinson’s diseases: a review. *Eur. J. Med. Chem.* **169**, 185–199 (2019). 30877973 10.1016/j.ejmech.2019.03.009

[616] Ly, C. et al. Bryostatin 1 promotes synaptogenesis and reduces dendritic spine density in cortical cultures through a PKC-dependent mechanism. *ACS Chem. Neurosci.* **11**, 1545–1554 (2020). 32437156 10.1021/acschemneuro.0c00175 PMC7332236

[617] Nelson, T. J. et al. Bryostatin effects on cognitive function and PKCvarepsilon in Alzheimer’s disease phase IIa and expanded access trials. *J. Alzheimers Dis.* **58**, 521–535 (2017). 28482641 10.3233/JAD-170161 PMC5438479

[618] Costa, A. C. & Scott-McKean, J. J. Prospects for improving brain function in individuals with Down syndrome. *CNS Drugs* **27**, 679–702 (2013). 23821040 10.1007/s40263-013-0089-3

[619] de la Torre, R. et al. Safety and efficacy of cognitive training plus epigallocatechin-3-gallate in young adults with Down’s syndrome (TESDAD): a double-blind, randomised, placebo-controlled, phase 2 trial. *Lancet Neurol.* **15**, 801–810 (2016). 27302362 10.1016/S1474-4422(16)30034-5

[620] Sharman, M. J. et al. Assessment of diets containing curcumin, epigallocatechin-3-gallate, docosahexaenoic acid and alpha-lipoic acid on amyloid load and inflammation in a male transgenic mouse model of Alzheimer’s disease: are combinations more effective? *Neurobiol. Dis.* **124**, 505–519 (2019). 30610916 10.1016/j.nbd.2018.11.026

[621] Fridman, J. S. et al. Selective inhibition of JAK1 and JAK2 is efficacious in rodent models of arthritis: preclinical characterization of INCB028050. *J. Immunol.* **184**, 5298–5307 (2010). 20363976 10.4049/jimmunol.0902819

[622] Simpson, E. L. et al. Efficacy and safety of abrocitinib in adults and adolescents with moderate-to-severe atopic dermatitis (JADE MONO-1): a multicentre, double-blind, randomised, placebo-controlled, phase 3 trial. *Lancet* **396**, 255–266 (2020). 32711801 10.1016/S0140-6736(20)30732-7

[623] Samuel, C., Cornman, H., Kambala, A. & Kwatra, S. G. A review on the safety of using JAK inhibitors in dermatology: clinical and laboratory monitoring. *Dermatol. Ther.* **13**, 729–749 (2023). 10.1007/s13555-023-00892-5 PMC9930707 36790724

[624] Walsh, R. R. et al. Plasma and cerebrospinal fluid pharmacokinetics of vodobatinib, a neuroprotective c-Abl tyrosine kinase inhibitor for the treatment of Parkinson’s disease. *Parkinsonism Relat. Disord.* **108**, 105281 (2023). 36717298 10.1016/j.parkreldis.2023.105281

[625] Lee, W. J. et al. Nilotinib treatment outcomes in autosomal dominant spinocerebellar ataxia over one year. *Sci. Rep.* **14**, 16303 (2024). 39009709 10.1038/s41598-024-67072-z PMC11251258

[626] Virlogeux, A. et al. Increasing brain palmitoylation rescues behavior and neuropathology in Huntington disease mice. *Sci. Adv*. **7**, eabb0799 (2021). 10.1126/sciadv.abb0799 PMC8011966 33789888

[627] Einat, H. et al. The role of the extracellular signal-regulated kinase signaling pathway in mood modulation. *J. Neurosci.* **23**, 7311–7316 (2003). 12917364 10.1523/JNEUROSCI.23-19-07311.2003 PMC6740453

[628] Fusco, F. R. & Paldino, E. Role of phosphodiesterases in Huntington’s disease. *Adv. Neurobiol.* **17**, 285–304 (2017). 28956337 10.1007/978-3-319-58811-7_11

[629] Gunther, R. et al. Rho kinase inhibition with Fasudil in the SOD1(G93A) mouse model of amyotrophic lateral sclerosis-symptomatic treatment potential after disease onset. *Front. Pharm.* **8**, 17 (2017). 10.3389/fphar.2017.00017 PMC5281550 28197100

[630] Lingor, P. et al. ROCK-ALS: protocol for a randomized, placebo-controlled, double-blind phase IIa Trial of safety, tolerability and efficacy of the Rho Kinase (ROCK) inhibitor Fasudil in amyotrophic lateral sclerosis. *Front. Neurol*. **10**, 293 (2019). 10.3389/fneur.2019.00293 PMC6446974 30972018

[631] Koch, J. C. et al. Safety, tolerability, and efficacy of fasudil in amyotrophic lateral sclerosis (ROCK-ALS): a phase 2, randomised, double-blind, placebo-controlled trial. *Lancet Neurol.* **23**, 1133–1146 (2024). 39424560 10.1016/S1474-4422(24)00373-9 PMC12741558

[632] Koch, J. C. et al. Compassionate use of the ROCK inhibitor Fasudil in three patients with amyotrophic lateral sclerosis. *Front. Neurol*. **11**, 173 (2020). 10.3389/fneur.2020.00173 PMC7083210 32231638

[633] Weisel, K. et al. Randomized clinical study of safety, pharmacokinetics, and pharmacodynamics of RIPK1 inhibitor GSK2982772 in healthy volunteers. *Pharmacol. Res. Perspect*. **5**, e00365 (2017). 10.1002/prp2.365 PMC5723699 29226626

[634] Vissers, M. et al. Safety, pharmacokinetics and target engagement of novel RIPK1 inhibitor SAR443060 (DNL747) for neurodegenerative disorders: randomized, placebo-controlled, double-blind phase I/Ib studies in healthy subjects and patients. *Clin. Transl. Sci.* **15**, 2010–2023 (2022). 35649245 10.1111/cts.13317 PMC9372423

[635] Trias, E. et al. Post-paralysis tyrosine kinase inhibition with masitinib abrogates neuroinflammation and slows disease progression in inherited amyotrophic lateral sclerosis. *J. Neuroinflamm.* **13**, 177 (2016). 10.1186/s12974-016-0620-9 PMC4940876 27400786

[636] Ketabforoush, A. et al. Masitinib: the promising actor in the next season of the Amyotrophic Lateral Sclerosis treatment series. *Biomed. Pharmacother.* **160**, 114378 (2023). 36774721 10.1016/j.biopha.2023.114378

[637] Mora, J. S. et al. Masitinib as an add-on therapy to riluzole in patients with amyotrophic lateral sclerosis: a randomized clinical trial. *Amyotroph. Lat. Scl FR* **21**, 5–14 (2020). 10.1080/21678421.2019.1632346 31280619

[638] Mora, J. S. et al. Long-term survival analysis of masitinib in amyotrophic lateral sclerosis. *Ther. Adv. Neurol. Disord*. **14**, 17562864211030365(2021). 10.1177/17562864211030365 PMC8388186 34457038

[639] Zeiser, R., Andrlova, H. & Meiss, F. Trametinib (GSK1120212). *Recent Results Cancer Res.* **211**, 91–100 (2018). 30069762 10.1007/978-3-319-91442-8_7

[640] Sun, C. et al. Reversible and adaptive resistance to BRAF(V600E) inhibition in melanoma. *Nature* **508**, 118–122 (2014). 24670642 10.1038/nature13121

[641] Nance, E., Pun, S. H., Saigal, R. & Sellers, D. L. Drug delivery to the central nervous system. *Nat. Rev. Mater.* **7**, 314–331 (2021). 38464996 10.1038/s41578-021-00394-w PMC10923597

[642] Ng, A. S. L. et al. Case-control analysis of leucine-rich repeat kinase 2 protective variants in Alzheimer’s disease. *Neurobiol. Aging* **64**, 157.e157–159 (2018). 10.1016/j.neurobiolaging.2017.11.012 29241968

[643] Lattante, S. et al. Novel variants and cellular studies on patients’ primary fibroblasts support a role for NEK1 missense variants in ALS pathogenesis. *Hum. Mol. Genet.* **30**, 65–71 (2021). 33445179 10.1093/hmg/ddab015

[644] Shao, W. et al. Two FTD-ALS genes converge on the endosomal pathway to induce TDP-43 pathology and degeneration. *Science* **378**, 94–99 (2022). 36201573 10.1126/science.abq7860 PMC9942492

[645] Yabe, I. et al. Spinocerebellar ataxia type 14 caused by a mutation in protein kinase C gamma. *Arch. Neurol.* **60**, 1749–1751 (2003). 14676051 10.1001/archneur.60.12.1749

[646] Martinez-Gonzalez, L. et al. Tideglusib, a non-ATP competitive inhibitor of GSK-3beta as a drug candidate for the treatment of amyotrophic lateral sclerosis. *Int. J. Mol. Sci*. **22**, 8975 (2021). 10.3390/ijms22168975 PMC8396476 34445680

